# Importance of biotic predictors in estimation of potential invasive areas: the example of the tortoise beetle *Eurypedus nigrosignatus*, in Hispaniola

**DOI:** 10.7717/peerj.6052

**Published:** 2018-12-05

**Authors:** Marianna V.P. Simões, A. Townsend Peterson

**Affiliations:** 1 Biodiversity Institute, University of Kansas, Lawrence, KS, USA; 2 Department of Marine Zoology, Senckenberg Research Institute and Goethe University, Frankfurt am Main, Hessen, Germany

**Keywords:** Akaike information criterion, Bayesian information criterion, Eltonian noise hypothesis, Ecological niche modeling, Model complexity, Invasive species, Hispaniola, Tortoise beetles

## Abstract

Climatic variables have been the main predictors employed in ecological niche modeling and species distribution modeling, although biotic interactions are known to affect species’ spatial distributions via mechanisms such as predation, competition, and mutualism. Biotic interactions can affect species’ responses to abiotic environmental changes differently along environmental gradients, and abiotic environmental changes can likewise influence the nature of biotic interactions. Understanding whether and how to integrate variables at different scales in ecological niche models is essential to better estimate spatial distributions of species on macroecological scales and their responses to change. We report the leaf beetle *Eurypedus nigrosignatus* as an alien species in the Dominican Republic and investigate whether biotic factors played a meaningful role in the distributional expansion of the species into the Caribbean. We evaluate ecological niche models built with an additive gradient of unlinked biotic predictors—host plants, using likelihood-based model evaluation criteria (Akaike information criterion and Bayesian information criterion) within a range of regularization multiplier parameter values. Our results support the argument that ecological niche models should be more inclusive, as selected biotic predictors can improve the performance of models, despite the increased model complexity, and show that biotic interactions matter at macroecological scales. Moreover, we provide an alternative approach to select optimal combination of relevant variables, to improve estimation of potential invasive areas using global minimum model likelihood scores.

## Introduction

Species distributions can be conceived as the intersection of three limiting factors: movement capacities, abiotic conditions, and biotic interactions. The latter comprise positive or negative interactions with other organisms, potentially influencing the persistence and growth of populations. Movement capacities define to which regions the species has had access, either at present or historically; finally, abiotic conditions define the areas in which the species will be able to maintain populations (Soberón & Peterson, 2005).

Ecological theory (Hutchinson, 1957) recognizes two main contrasting subsets (corresponding to two of the three constraints just listed) that may have dominant roles at distinct spatial scales. The first is defined by ecological interactions and resource consumption (“Eltonian niche”; Elton, 1927), whereas the second is defined by the abiotic environment, imposing physiological restrictions on the establishment, survivorship, and reproduction of individuals (“Grinnellian niche” *sensu* Soberón, 2007). The relative importance of biotic vs. abiotic factors is influenced by spatial scale and resolution: fine-scale studies generally indicate greater importance of biotic interactors, whereas coarse-scale studies emphasize abiotic factors (Pearson & Dawson, 2003; Soberón, 2007; Soberón & Nakamura, 2009). Climatic variables have been the main focus in ecological niche modeling and species distributional modeling, whereas biotic interactions are usually not considered (Pearson & Dawson, 2003; Soberón, 2007; Soberón & Nakamura, 2009).

Biotic interactions, however, may affect species’ distributions via mechanisms such as predation, competition, parasitism, and mutualism (Bascompte, 2009; Van Dam, 2009; Wisz et al., 2013). Addition of this sort of information to species distribution modeling (SDM) estimations has shown contrasting results, affecting predictive power of models positively (Heikkinen et al., 2007; Araújo & Luoto, 2007; Giannini et al., 2013; Fraterrigo, Wagner & Warren, 2014; D’Amen et al., 2017; Atauchi, Peterson & Flanagan, 2018), negatively (Pearson & Dawson, 2003; Araújo & Luoto, 2007; Pellissier et al., 2010; Silva et al., 2014), or not at all (Pellissier et al., 2010; Araújo & Luoto, 2007). Cases in which models are based solely on abiotic factors at coarse spatial scales often show good predictive power, emphasizing the importance of abiotic factors (Pellissier et al., 2010). The idea that distributions of species on geographic extents and coarse resolutions are rarely affected significantly by biotic factors has been termed the “Eltonian noise hypothesis” (ENH; Soberón & Nakamura, 2009).

On the other hand, Anderson (2017) highlighted the importance of biotic interactions in shaping species’ distributions. He contended that, in many interactions, one of the species is little affected by the population of the other species (e.g., a host plant has its distribution independent of specific phytophagous insect distributions), and thus obey climatic predictors closely. Thus, he advocated addition of such biotic variables as unlinked predictors to SDMs to increase predictive power of models.

**Figure 1:**
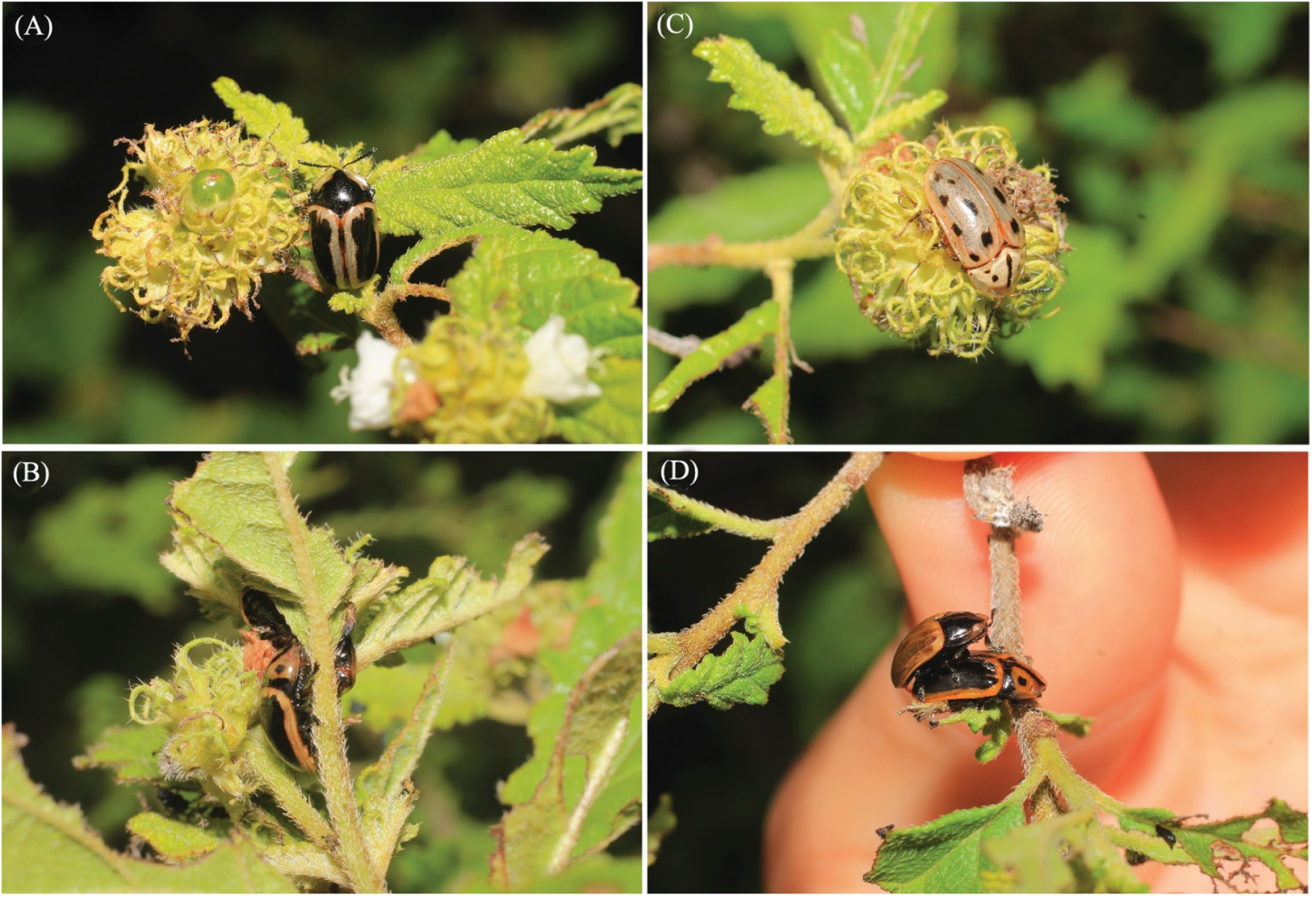
Individuals of *Eurypedus nigrosignatus* found feeding and copulating on the native plant species *Cordia curassavica* (Jacq.) Roem. & Schult. (Boraginaceae), in the Parque Nacional del Este, in the eastern part of the Dominican Republic. (A–C) distinct morphotypes of *E. nigrosignatus*, feeding on the host plant, *Cordia curassavica*; (D) individuals of *E. nigrosignatus* copulating on top of host plant. Photo credit: Marianna Simões.

The Neotropical tortoise beetle genus *Eurypedus* Gistel includes two species with disjunct distributions, north and south of the Amazon Basin: *Eurypedus nigrosignatus* (Boheman) and *E. peltoides* (Boheman), respectively (Shin, 2016). During fieldwork by the first author (MS) in June 2015, the three morphotypes of *E. nigrosignatus* (Boheman) were found feeding on the native plant species *Cordia curassavica* (Jacq.) Roem. & Schult. (Boraginaceae), in the Parque Nacional del Este, in the eastern part of the Dominican Republic (Fig. 1). Despite extensive sampling in previous studies in that area (Blake, 1937, 1938, 1939; Peck, 2006; Peck & Perez-Gelabert, 2012), this record is the first of this tortoise beetle species to Dominican Republic. Thus, we report it as an alien species—species establishing viable populations in areas beyond their normal biogeographic barriers to spread (Blackburn et al., 2011).

The purpose of this study was to investigate the relative importance of biotic and abiotic factors in delimiting the distribution of *E. nigrosignatus*, particularly regarding its expansion into the Caribbean. We used likelihood-based model evaluation criteria (Akaike information criterion (AICc) and Bayesian information criterion (BIC); Warren & Seifert, 2011), evaluating an additive gradient of biotic predictors and different algorithm parameters in ecological niche estimation. Our goal was to understand whether inclusion of different numbers and combinations of biotic predictors affected models positively or negatively. We interpret our results in terms of whether adding known biotic interactors can improve predictions of suitable invasive habitats for this particular insect species.

## Materials and Methods

### Distributional data

We compiled 33 occurrence records (Supporting Information, Table S1) for *E. nigrosignatus* from Shin (2016), which presents a detailed taxonomic revision of the genus *Eurypedus* with a thorough evaluation of its geographic distribution. The study counted on the examination of 220 specimens from 12 museums (American Museum of Natural History, New York, NY, USA; Brigham Young University, Monte L. Bean Life Science Museum, Provo, UT, USA; Collection of Edward G. Riley, TX, USA; Division of Plant Industry, Florida State Collection of Arthropods, Gainesville, FL, USA; Hungarian Natural History Museum, Budapest, Hungary; National Museum of Natural History, Washington, D.C., USA; Museum of Comparative Zoology, Harvard University, Cambridge, MA, USA; Naturhistoriska Riksmuseet, Stockholm, Sweden; Natural History Museum, Texas A&M University, College Station, TX, USA; Universidade Federal do Paraná, Museu de Entomologia Pe. Jesus Santiago Moure, Curitiba Paraná, Brazil; Universidade Federal do Rio de Janeiro, Museu Nacional, Sao Cristóvão, Rio de Janeiro, Brazil; University of Kansas, Lawrence, KS, USA). Thus, the small amount of occurrence points is mostly due to the scarcity of the species in natural environments.

Historical records lacking geographic coordinates were georeferenced via consultation of Global Gazetteer (http://www.fallingrain.com/world/) and Google Earth (https://www.google.com/earth/). For confirmed host plants of the tortoise beetle species (Shin, 2016), georeferenced distributional records were obtained from the Global biodiversity information facility (GBIF, www.gbif.org) and *speciesLink* (http://www.splink.cria.org.br/). In all, we found 1,959 records of *C. curassavica* (Jacq.), 296 of *C. inermis* (Mill.), 1,753 of *C. spinescens* L. (Boraginaceae) and 5,566 of *Melanthera nivea* L. (Compositae). After a series of steps of cleaning and error detection (e.g., removal of duplicate records, numerical sign confusion), we had 587 records of *C. curassavica*, 282 of *C. inermis*, 1,027 of *C. spinescens*, and 2,274 of *M. nivea* (Table S2).

Despite previous sampling efforts (Blake, 1937, 1938, 1939; Perez-Gelabert, 2008; Peck & Perez-Gelabert, 2012), and geographic proximity between the species native range and invaded area, there are no previous records of the species to Hispaniola. Here we report *E. nigrosignatus* as an alien species to the Dominican Republic fauna. About 50 individuals were encountered feeding on the plant species *C. curassavica* (Jacq.) Roem. & Schult. (Boraginaceae), in the Parque Nacional del Este, in the eastern part of the Dominican Republic (Fig. 1), during field work performed by the first author (MS), in June 2015. The fieldwork was performed with collecting permit granted by the ministry of environment and natural resources of the Dominican Republic. The host plant was identified on field by Dr. Francisco Jiménez (Jardin Botánico Nacional, Dominican Republic).

### Environmental data

Environmental data were obtained at 5 arc-minute (∼10 km) spatial resolution for this study from WorldClim (version 1.3, http://www.worldclim.org; Hijmans et al., 2005). WorldClim is based on interpolations of weather station data (i.e., monthly precipitation and minimum and maximum temperatures) over the period 1950–2000. Of the 19 available bioclimatic variables, we excluded four (mean temperature of wettest quarter, mean temperature of driest quarter, precipitation of warmest quarter, precipitation of coldest quarter), owing to known spatial artifacts (Campbell et al., 2015). To avoid overfitting and inflation of model accuracy with overly dimensional environmental spaces and collinearity among variables, we performed a principal component analysis (PCA) on the correlation matrix of the remaining 15 environmental variables using the *PCARaster* function in the package “ENMGadgets” (Barve & Barve, 2013) in R 3.3.1 (R Core Team, 2018) software. We retained the first five components, which explained cumulatively >95% of the total variance in the dataset, for model calibration.

### Ecological niche modeling

To generate models, we used MaxEnt version 3.3.3.k (Phillips, Anderson & Schapire, 2006), implemented in the R package “dismo” (Hijmans et al., 2016), known to perform well with small sample sizes, as well as when using small predictor variable datasets (Jackson & Robertson, 2011; Bean, Stafford & Brashares, 2012; Platts et al., 2014; Van Proosdij et al., 2016). The parameters in Maxent were kept at default settings and in model calibration, we used 10 replicate bootstrap runs using 20% of calibration data (i.e., total number of occurrence records). Because the total number of training sites was low, the bootstrap replication technique, which involves sampling from the original occurrence locations with replacement, helped to avoid losing valuable training data for model development. We used individual calibration areas to model *E. nigrosignatus* and each of the host plant species (Barve et al., 2011). For *E. nigrosignatus*, calibration area was defined as the known area of distribution delineated by Shin (2016), plus 10 km buffer around them as a proxy of the species’ very limited dispersal capacity; for each host plant we took the area in which the species occurrence points fell, plus 50 km buffer around them.

Models were generated in raw output for model selection, and in logistic output format for model evaluation (Phillips & Dudík, 2008). In model calibration, we used 100 replicate bootstrap analyses, which allows testing the model with occurrences that may have been used for training, and default regularization multiplier parameter (β = 1). Then, we created additive models, based on the environmental layers, in addition to the median raster of each host plant species model, totaling 16 models of four complexity levels (one, two, three, or four biotic interactors). All models were run with five different β values (0.1, 0.5, 1, 2, and 5), to avoid overfitting with the increase in complexity (Phillips, 2005), totaling 80 models. Binary maps were derived from continuous median models from Maxent by applying the 10-percentile training presence value as threshold for visualization and comparison the extent of areas estimated to be of potential distribution to *E. nigrosignatus*.

### Model selection

Model selection was performed using ENMTools following Warren & Seifert (2011). We applied the BIC and sample-size-corrected AICc for Maxent models (Akaike, 1974; Burnham & Anderson, 2002). These metrics count all parameters with nonzero weights in the lambda file produced by Maxent (Warren, Glor & Turelli, 2010) and penalize likelihood values based on the increase in model complexity.

### Model evaluation

Owing to the poor reflection of model accuracy by the receiver operating characteristic (ROC) area under the curve (AUC) approach (Lobo, Jiménez-Valverde & Real, 2008), we used partial receiver operating characteristic (Partial ROC) approaches (Peterson, Papeş & Soberón, 2008). This method allows assessment of predictive ability of niche models, considering only omission error and proportional area predicted as suitable, but only over a range of omission error deemed acceptable considering the error characteristics of the input data. We used a Visual Basic routine developed by N. Barve (University of Kansas, Lawrence, KS; available via http://hdl.handle.net/1808/10059), using an acceptable omission error threshold of *E* = 5% (Peterson, Papeş & Soberón, 2008), with 100 replicates, each based on resampling 50% of test points with replacement, which maximizes replicate-to-replicate variation and maintains sample sizes relatively high (Peterson, Papeş & Soberón, 2008).

### Biotic interactor relevance

To estimate the relevance and contribution of each host plant to the model likelihood, within the same category of predictor richness, we summed the average of model likelihoods when including a host plant and subtracted the average of model likelihoods excluding such host plant. This step allowed distinguishing the largest negative difference among all the models that include a given host plant and identifying the most relevant biotic interactor(s) to calibrate ecological niche models for *E. nigrosignatus*.

## Results

**Figure 2:**
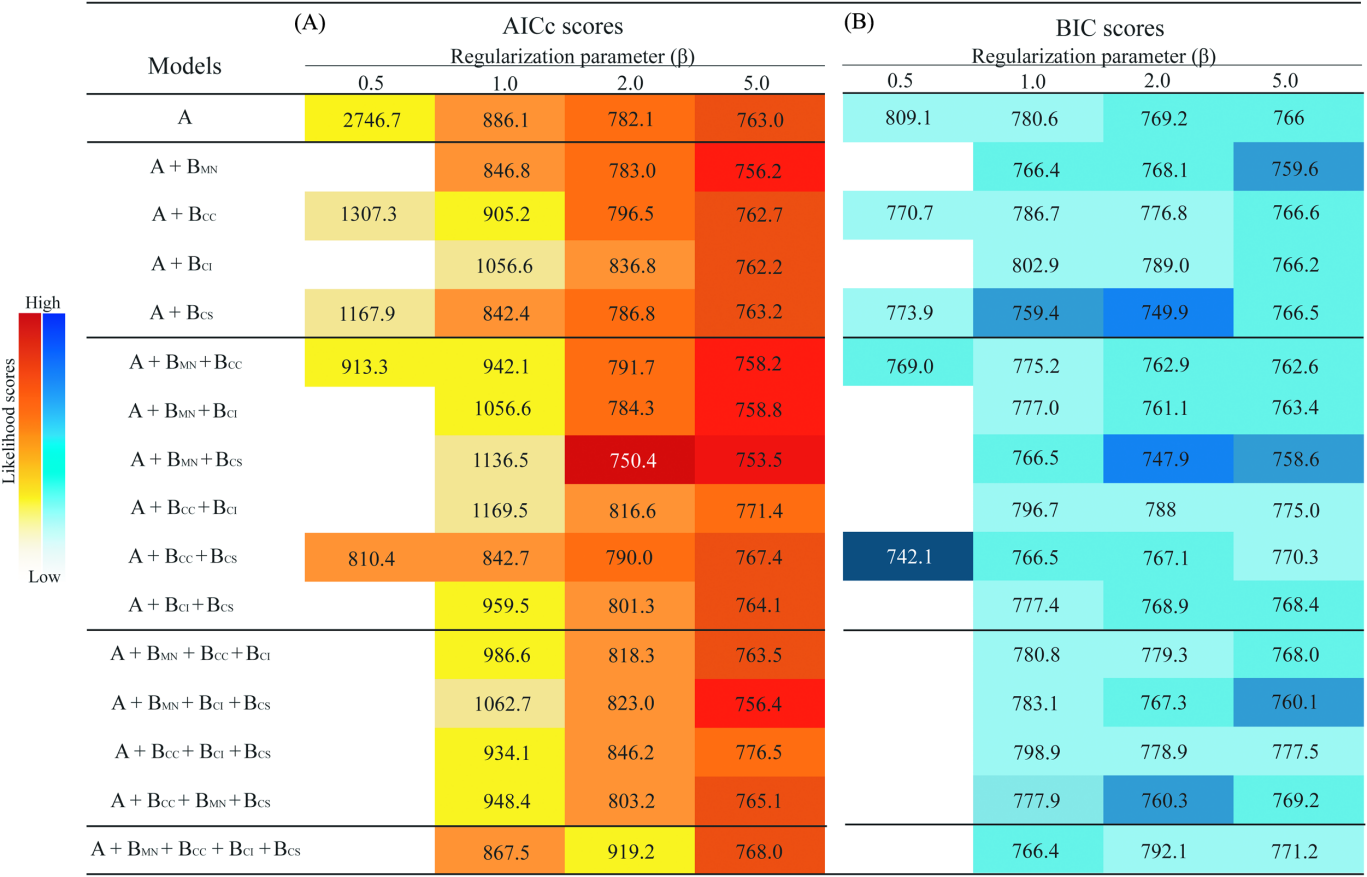
Summary of information criteria for different models of the ecological niche of *Eurypedus nigrosignatus*. (A) AICc values. (B) BIC values resulting from models with different levels of complexity. Darker colors indicate lower likelihood and lighter color indicates higher likelihood values. Predictor richness is indicated on the vertical left side of table, where, A, abiotic variables; B_MN_, biotic interactor, *M. nivea*; B_CC_, biotic interactor, *C. curassavica*; B_CI_, biotic interactor, *C. inermis*; B_CS_, biotic interactor, *C. spinescens*.

The models generated with β = 0.1 and some models generated with β = 0.5, given the elevated model complexity and low number of occurrence points (*n* = 33), did not allow recovery of AICc or BIC values because the number of parameters exceeded the sample size, so they were excluded from analysis. Models generated using β values of 1, 2, or 5 produced more generalized predictions, allowing estimation of AICc and BIC for all models and complexity levels (i.e., one, two, three, or four biotic interactors) (Fig. 2).

Models including biotic interactors yielded higher likelihood scores (i.e., lower AICc values) than models generated using environmental variables only and the global minimum included two host plants, *M. nivea* and *C. spinescens* (Fig. 2). The minimum AICc and BIC with β = 1 was from a model including one host plant, *C. spinescens* (AICc = 842.4, BIC = 759.4); minimum AICc and BIC with β of two and five were from models including two host plants, *M. nivea* and *C. spinescens* (β = 2, AICc = 750.4, BIC = 747.9; β = 5, AICc = 753.5, BIC = 758.6). Measurement of host plant relevance revealed *M. nivea* as the most relevant biotic interactor, showing the largest negative difference of likelihood when included as predictor in combination with abiotic variables. *Cordia spinescens* was the second most relevant biotic interactor, and in combination with *M. nivea* yield the model with the largest negative difference of likelihood. The average model likelihood excluding *M. nivea* was −39.4 (AICc), and the average including *M. nivea* from model estimation was −235.8 (AICc) (Table 1).

**Table 1:** Relative relevance of each host plant in different levels of model complexity.

Predictor richness	Regularization parameter (β)			
	0.5	1	2	5
A	53.3	59.4	93.3	116.7
A + B_MN_	29.8	105.6	124.5	109.3
A + B_VC_	81.1	102.6	101.1	97.9
A + B_VI_	77.4	183.0	110.1	116.1
A + B_VS_	57.4	98.5	77.7	91.9
A + B_MN_ + B_VC_	61.8	102.6	126.9	122.9
A + B_MN_ +B_VI_	40.7	99.6	132.6	120.1
A + B_MN_ + B_VS_	85.3	114.1	115.1	138.9
A + B_VC_ + B_VI_	92.7	129.4	85.3	77.3
A + B_VC_ + B_VS_	75.4	58.4	74.1	112.9
A + B_VI_ + B_VS_	97.6	65.9	91.1	80.4
A + B_MN_ + B_VC_ + B_VI_	44.1	66.6	121.7	117.2
A + B_MN_ + B_VI_ + B_VS_	84.9	95.9	93.5	136.4
A + B_VC_ + B_VI_ + B_VS_	46.1	79.7	78.2	97.2
A + B_VC_ + B_MN_ + B_VS_	74.2	108.2	126.6	126.2
A + B_MN_ + B_VC_ + B_VI_ + B_VS_	67.6	84.2	128.2	135.4

**Figure 3:**
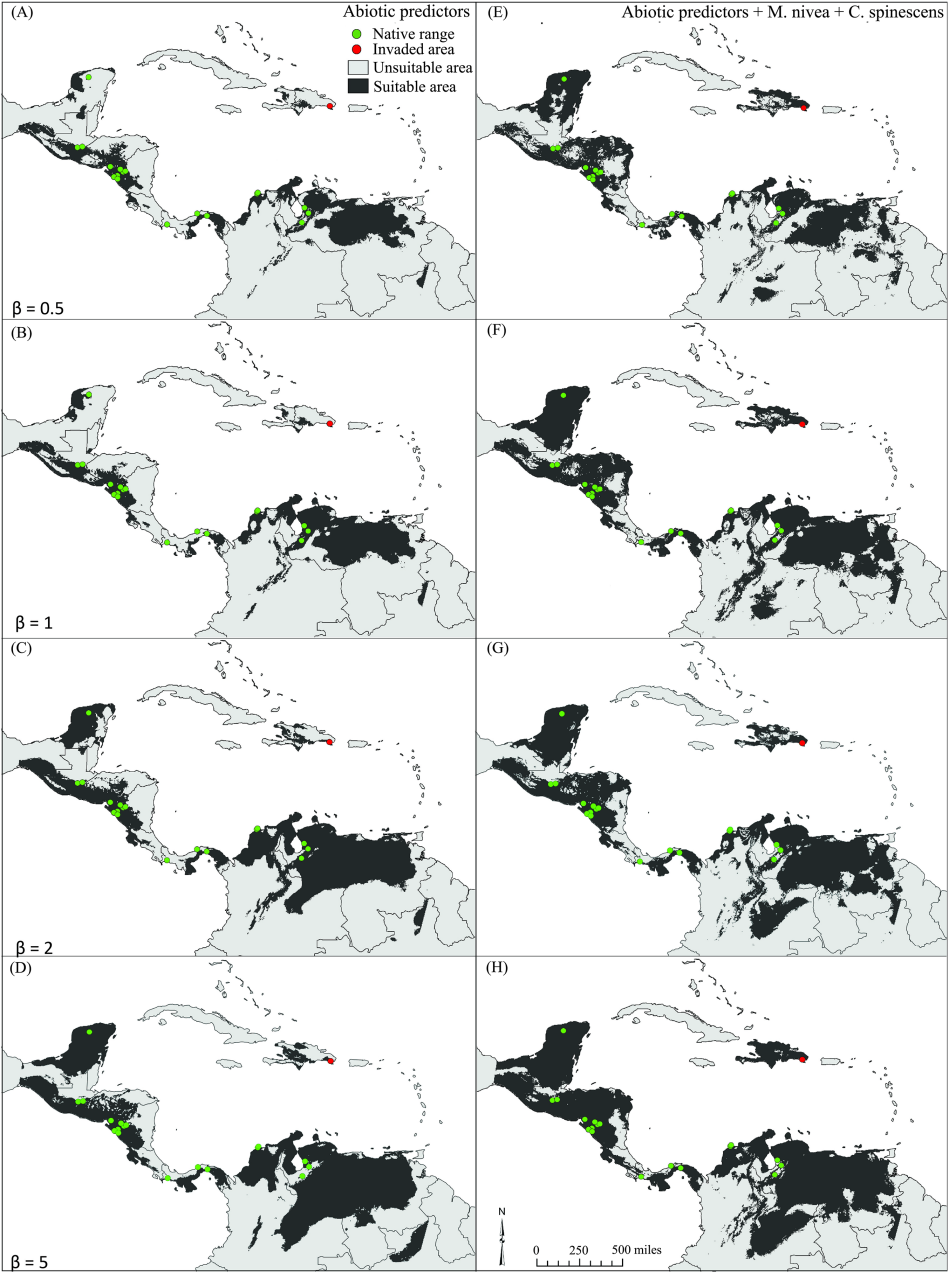
Binary maps showing potential distribution of *Eurypedus nigrosignatus*. (A–D) models estimated using only abiotic predictors. (E–H) Models estimated using abiotic predictors along with optimal combination of biotic predictors, under four regularization parameter values (0.5, 1, 2, and 5).

All model predictions reflected well the known distribution of *E. nigrosignatus*, with partial ROC and AUC values significantly better than random (i.e., partial ROC all AUC > 1; *P* < 0.01; Table S3). Binary maps of models including biotic interactors yielded larger areas of suitability (Table 2), and the invasive area in the Dominican Republic was detected conspicuously only in models including biotic interactors (Fig. 3).

**Table 2:** Area (in km^2^) of suitable habitat after model thresholding (*E* = 10%) in relation to different regularization parameter values and predictor richness.

	Including β = 0.5		Excluding β = 0.5	
	AICc	BIC	AICc	BIC
B_MN_	**−235.8**	**−135.5**	**−39.4**	−9.1
B_MN_ + 1	13.1	−6.4	5.5	−11.5
B_MN_ + 2	−9.8	−8.1	13.2	−13.3
B_CC_	226.1	133.4	104.6	134.9
B_CC_ + 1	134.3	132.2	−12.8	8.3
B_CC_ + 2	−38.8	7.8	−26.5	6.6
B_CI_	−145.9	−114.2	80.4	19.4
B_CI_ + 1	−114.8	−117.7	38.8	10.9
B_CI_ + 2	76.1	6.0	6.3	8.0
B_CS_	155.6	116.3	−36.5	**−17.2**
B_CS_ + 1	−32.6	−8.1	−31.5	−7.8
B_CS_ + 2	−13.7	−0.9	7.9	−1.2

**Notes:**

Numbers in bold indicate largest difference among models including or excluding the host plant. Differences were calculated by summing average of model likelihoods within the same category of model complexity, including a host plant and subtracted the average of model likelihoods excluding such host plant. As a result, the host plant generating the largest negative difference is *Melanthera nivea*, and second largest, *Cordia spinescens*.

B_MN_, biotic interactor, *M. nivea*; B_CC_, biotic interactor, *C. curassavica*; B_CI_, biotic interactor, *C. inermis*; B_CS_, biotic interactor, *C. spinescens*).

## Discussion

### Integration of unlinked biotic interactors to model estimations

As species’ geographic distributions are understood to coincide in large part with the intersection of their movement capacities, abiotic conditions, and biotic interactions (Hutchinson, 1957; Soberón & Peterson, 2005), understanding how to integrate these three sets of variables in ecological niche models is essential. Previous studies investigating implications of biotic interactions in niche modeling have offered evidence that the explanatory power of models at scales could be improved with addition of information on biotic interactors (Leathwick, 2002; Meier et al., 2010; Jaeschke et al., 2012; Giannini et al., 2013; Lira-Noriega, Soberón & Miller, 2013; Mpakairi et al., 2017; Atauchi, Peterson & Flanagan, 2018).

Our results support the argument that integration of selected, unlinked biotic interactors in ecological niche models can improve model performance, despite the increase in model complexity. As such, we partially reject the predictions of the ENH, showing that biotic interactions matter at macroecological scales. However, it is important to note that some models including biotic interactors performed worse than models including only abiotic variables, which reinforces results of previous studies showing that inclusion of biotic can affect the predictive power of models positively, negatively, or neutrally (Araújo & Luoto, 2007). Here, we identified *M. nivea* and *C. spinescens* as best model contributors amongst biotic interactors, yielding the largest difference of likelihood when added as predictors to model estimations. They also, in combination with abiotic variables, yielded the global minimum in likelihood and the best model predictions. Models based solely on abiotic variables performed poorly and did not detect the invaded area in the Dominican Republic, except at β = 5, the most permissive level tested in our study, whereas models including biotic predictors detected the invasive area at all levels of β.

We further speculate that the role of the ENH idea may be contingent to the specific type of interaction and depth of knowledge regarding biotic interactors included in the model. Plants comprise important dimensions of the ecological niches of herbivorous insects, serving as hosts and substrates for important parts of the insect’s life cycle (Kergoat, Meseguer & Jousselin, 2017). Geographic turnover in host plant suitability and availability often results in populations of herbivorous insects that are locally adapted to different host plants across the insect’s geographic range (Thompson, 2005; Pelini et al., 2010). Therefore, species such as *E. nigrosignatus* may require a certain host for successful development of its larvae, but the particular species of plant does not matter for other aspects of its life cycle (e.g., shelter for hibernation or against potential predators) (Pelini et al., 2010), creating a geographic mosaic of host plant degree of interaction (Thompson, 2005; Bascompte & Jordano, 2007).

Anderson (2017) suggested that creating a response surface to represent statistically the relationship between biotic interactors and abiotic variables could mirror estimates of the response of the focal species to biotic factors. However, estimation of such surfaces has limited practicality, as it requires broad knowledge regarding biotic interactions and population-level effects, detailed and specific information that is rare or limited in most taxa (Peterson et al., 2011; Fordham et al., 2012). As an alternative, we propose further exploration of complexity-penalized likelihood metrics to evaluate combinations of abiotic variables and biotic interactors, allowing researchers to identify best combinations among different biotic interactors and abiotic variables.

We understand that the integrative approach suggested here is not only theoretical but also a practical challenge in the construction of SDMs (De Araújo, Marcondes-Machado & Costa, 2014), as the sort and level of the interaction between the biotic interactors and target species must be well known. The genus *Eurypedus* has been through a detailed taxonomic revision and thorough evaluation of its geographic distribution (Shin, 2016), so host plants associated with its species are well studied. However, despite great economical relevance and prominence in ecology and evolutionary literature, identification of biotic interactors of herbivorous insects, and the essence of their interaction (e.g., host plants as food source, oviposition sites or shelter for hibernation) remain poorly known (Kergoat, Meseguer & Jousselin, 2017). The lack of such information hinders the development of targeted monitoring programs for species at high risk of being invasive pests and the preservation of areas that could represent areas of potential distribution for pest species.

### *Eurypedus nigrosignatus* in the Dominican Republic

Based in the field observations in Panama, adults of *E. nigrosignatus* appear to bury themselves around their host plants during dry season (January–April; Gómez, 2004). Then, over the first three subsequent months of the rain season, ovipositing adults and immature stages become abundant and conspicuous on the host plant. Egg masses of 5–29 eggs are deposited on leaves, with average survival to adult eclosion of only 2% (Gómez, 2004; Świętojańska et al., 2018). Thus, populations of this species are usually low in number of individuals and not conspicuous on field.

The population of *E. nigrosignatus* found in June 2015—wet season in the Dominican Republic (Latta & Faaborg, 2001)—was large, with ca. 40–50 adults and no immatures. The composition and abundance of individuals suggest that the population was at the beginning of its seasonal life cycle, with adults emerging from the surrounding soil for oviposition, characterizing a well-established population. The absence of previous record of this species to Hispaniola could be due to a lack of target sampling or incongruency between species biology and field surveys. However, extensive sampling efforts throughout different seasons of the year (Blake, 1937, 1938, 1939), museum collection material (220 specimens from 12 museums) and literature review (Perez-Gelabert, 2008; Peck & Perez-Gelabert, 2012), suggest that this is not the case.

Our results show that, the presence of *E. nigrosignatus* on the east side of the Dominican Republic is best explained by the presence of biotic interactors. We further speculate that the invasion of the species to the Dominican Republic is recent, and possibly associated with the increased exchange of agricultural goods. However, as in many cases of arthropod species, the time of arrival cannot be ascertained (Serra et al., 2003).

## Conclusions

Our results support the argument that relevant unlinked interactors should be included to SDM estimation, as selected biotic predictors can improve the performance of models, despite increasing its complexity. We provide evidence that likelihood-based model evaluation criteria is useful for selection of optimal combination of variables, while taking into account model complexity, providing more accurate estimations of invaded or potentially invasible areas.

## Supplemental Information

10.7717/peerj.6052/supp-1 Supplemental Information 1 Occurrence data for *Eurypedus nigrosignatus*.

10.7717/peerj.6052/supp-2 Supplemental Information 2 Occurrence data for host plants: *Cordia curassavica*, *C. inermis*, *C. spinenscens* and *Melanthera nivea*. These occurrence data were used as predictor variables in our efforts to estimate the ecological niches for the insect species.

10.7717/peerj.6052/supp-3 Supplemental Information 3 Mean AUC ratios (AUC), and partial ROC analyses for different candidate models of the study on different levels of regularization parameter (*β*).

## Additional Information and Declarations

### Field Study Permissions

The following information was supplied relating to field study approvals (i.e., approving body and any reference numbers): Field work was approved by the Ministerio de Medio Ambiente y Recursos Naturales Vice-ministerio de Areas Protegidas y Biodeiversidad República Dominicana

### Data Availability

The following information was supplied regarding data availability: Raw data are provided in the Materials and Methods of the article as well as in Tables S1–S3.
