# Supplementary material for: Importance of biotic predictors in estimation of potential invasive areas: the example of the tortoise beetle Eurypedus nigrosignatus, in Hispaniola
Source: PeerJ. 2018 Dec 5;6:e6052. doi: 10.7717/peerj.6052 (PMC6286658; doi:10.7717/peerj.6052)
Supplement: Supplemental Information 1 [file peerj-06-6052-s001.docx]

**Importance of biotic predictors in estimation of potential invasive areas: the example of the tortoise beetle *Eurypedus nigrosignatus,* in Hispaniola**

Marianna V. P. Simões & A. Townsend Peterson

**Supporting information**

**Supporting information, Table S1.** Occurrence data for *Eurypedus nigrosignatus.*

| **Species** | **Longitude** | **Latitude** |
| --- | --- | --- |
| *E. nigrosignatus* | -86.60 | 12.56 |
| *E. nigrosignatus* | -86.89 | 12.45 |
| *E. nigrosignatus* | -70.59 | 9.39 |
| *E. nigrosignatus* | -86.89 | 12.45 |
| *E. nigrosignatus* | -86.88 | 12.44 |
| *E. nigrosignatus* | -86.89 | 12.45 |
| *E. nigrosignatus* | -86.89 | 12.45 |
| *E. nigrosignatus* | -70.93 | 9.85 |
| *E. nigrosignatus* | -86.89 | 12.45 |
| *E. nigrosignatus* | -86.89 | 12.45 |
| *E. nigrosignatus* | -86.88 | 12.44 |
| *E. nigrosignatus* | -86.89 | 12.45 |
| *E. nigrosignatus* | -86.10 | 12.85 |
| *E. nigrosignatus* | -86.89 | 12.45 |
| *E. nigrosignatus* | -70.93 | 9.85 |
| *E. nigrosignatus* | -86.89 | 12.45 |
| *E. nigrosignatus* | -86.89 | 12.45 |
| *E. nigrosignatus* | -86.89 | 12.45 |
| *E. nigrosignatus* | -86.89 | 12.45 |
| *E. nigrosignatus* | -89.94 | 14.92 |
| *E. nigrosignatus* | -74.88 | 11.02 |
| *E. nigrosignatus* | -74.83 | 11.11 |
| *E. nigrosignatus* | -68.79 | 18.38 |
| *E. nigrosignatus* | -89.53 | 14.97 |
| *E. nigrosignatus* | -87.18 | 13.30 |
| *E. nigrosignatus* | -89.00 | 20.83 |
| *E. nigrosignatus* | -86.35 | 13.08 |
| *E. nigrosignatus* | -86.57 | 12.27 |
| *E. nigrosignatus* | -85.92 | 12.92 |
| *E. nigrosignatus* | -82.43 | 8.43 |
| *E. nigrosignatus* | -79.90 | 9.36 |
| *E. nigrosignatus* | -79.10 | 9.17 |
| *E. nigrosignatus* | -71.15 | 8.60 |
