# Supplementary material for: Importance of biotic predictors in estimation of potential invasive areas: the example of the tortoise beetle Eurypedus nigrosignatus, in Hispaniola
Source: PeerJ. 2018 Dec 5;6:e6052. doi: 10.7717/peerj.6052 (PMC6286658; doi:10.7717/peerj.6052)
Supplement: Supplemental Information 2 — These occurrence data were used as predictor variables in our efforts to estimate the ecological niches for the insect species. [file peerj-06-6052-s002.docx]

**Importance of biotic predictors in estimation of potential invasive areas: the example of the tortoise beetle *Eurypedus nigrosignatus,* in Hispaniola**

Marianna V. P. Simões & A. Townsend Peterson

**Supporting information**

**Supporting information, Table S2.** Occurrence data for host plants: *Cordia curassavica*, *C. inermis*, *C. spinenscens* and *Melanthera nivea*. These occurrence data were used as predictor variables in our efforts to estimate the ecological niches for the insect species.

| **Species** | **Longitude** | **Latitude** |
| --- | --- | --- |
| *Cordia curassavica* | -40.50 | -11.16 |
| *Cordia curassavica* | -63.45 | -18.12 |
| *Cordia curassavica* | -48.52 | -22.96 |
| *Cordia curassavica* | -48.43 | -22.84 |
| *Cordia curassavica* | -97.26 | 18.19 |
| *Cordia curassavica* | -39.86 | -18.67 |
| *Cordia curassavica* | -48.42 | -22.83 |
| *Cordia curassavica* | -43.00 | -22.00 |
| *Cordia curassavica* | -38.88 | -12.37 |
| *Cordia curassavica* | -42.84 | -22.96 |
| *Cordia curassavica* | -73.02 | 6.72 |
| *Cordia curassavica* | -39.70 | -12.75 |
| *Cordia curassavica* | -37.14 | -10.84 |
| *Cordia curassavica* | -35.68 | -8.40 |
| *Cordia curassavica* | -35.68 | -8.40 |
| *Cordia curassavica* | -76.51 | 2.41 |
| *Cordia curassavica* | -48.59 | -25.93 |
| *Cordia curassavica* | -88.41 | 17.52 |
| *Cordia curassavica* | -40.97 | -9.18 |
| *Cordia curassavica* | -71.30 | 6.01 |
| *Cordia curassavica* | -40.07 | -19.39 |
| *Cordia curassavica* | -41.85 | -11.18 |
| *Cordia curassavica* | -42.29 | -22.94 |
| *Cordia curassavica* | -42.22 | -22.94 |
| *Cordia curassavica* | -40.73 | -20.86 |
| *Cordia curassavica* | -40.50 | -11.18 |
| *Cordia curassavica* | -48.35 | -25.55 |
| *Cordia curassavica* | -40.52 | -18.95 |
| *Cordia curassavica* | -48.49 | -27.15 |
| *Cordia curassavica* | -40.14 | -18.90 |
| *Cordia curassavica* | -40.13 | -19.87 |
| *Cordia curassavica* | -73.07 | 7.01 |
| *Cordia curassavica* | -73.03 | 7.02 |
| *Cordia curassavica* | -40.26 | -20.02 |
| *Cordia curassavica* | -40.88 | -19.23 |
| *Cordia curassavica* | -40.54 | -20.06 |
| *Cordia curassavica* | -44.24 | -20.14 |
| *Cordia curassavica* | -40.54 | -20.35 |
| *Cordia curassavica* | -42.29 | -22.94 |
| *Cordia curassavica* | -37.32 | -10.76 |
| *Cordia curassavica* | -48.51 | -25.67 |
| *Cordia curassavica* | -40.40 | -8.26 |
| *Cordia curassavica* | -39.77 | -19.57 |
| *Cordia curassavica* | -47.08 | -24.44 |
| *Cordia curassavica* | -43.96 | -19.46 |
| *Cordia curassavica* | -46.80 | -24.18 |
| *Cordia curassavica* | -46.80 | -24.17 |
| *Cordia curassavica* | -49.93 | 1.67 |
| *Cordia curassavica* | -36.37 | -9.84 |
| *Cordia curassavica* | -54.14 | 5.41 |
| *Cordia curassavica* | -73.45 | 7.82 |
| *Cordia curassavica* | -73.18 | 6.90 |
| *Cordia curassavica* | -58.04 | -28.03 |
| *Cordia curassavica* | -88.19 | 21.57 |
| *Cordia curassavica* | -110.85 | 27.90 |
| *Cordia curassavica* | -46.36 | -12.78 |
| *Cordia curassavica* | -79.93 | 8.68 |
| *Cordia curassavica* | -41.19 | -11.58 |
| *Cordia curassavica* | -60.65 | -16.88 |
| *Cordia curassavica* | -39.06 | -11.63 |
| *Cordia curassavica* | -39.54 | -18.07 |
| *Cordia curassavica* | -39.54 | -18.70 |
| *Cordia curassavica* | -96.15 | 15.75 |
| *Cordia curassavica* | -43.34 | -22.87 |
| *Cordia curassavica* | -40.44 | -10.57 |
| *Cordia curassavica* | -63.67 | -18.31 |
| *Cordia curassavica* | -38.96 | -16.11 |
| *Cordia curassavica* | -38.96 | -16.11 |
| *Cordia curassavica* | -41.77 | -14.19 |
| *Cordia curassavica* | -88.76 | 19.24 |
| *Cordia curassavica* | -97.01 | 17.96 |
| *Cordia curassavica* | -93.85 | 16.26 |
| *Cordia curassavica* | -37.21 | -11.01 |
| *Cordia curassavica* | -101.90 | 19.33 |
| *Cordia curassavica* | -89.22 | 19.22 |
| *Cordia curassavica* | -96.19 | 15.77 |
| *Cordia curassavica* | -110.72 | 24.78 |
| *Cordia curassavica* | -79.39 | 9.20 |
| *Cordia curassavica* | -43.84 | -20.21 |
| *Cordia curassavica* | -40.30 | -13.93 |
| *Cordia curassavica* | -49.24 | -16.68 |
| *Cordia curassavica* | -97.01 | 17.95 |
| *Cordia curassavica* | -40.73 | -20.07 |
| *Cordia curassavica* | -39.54 | -15.16 |
| *Cordia curassavica* | -73.07 | 7.04 |
| *Cordia curassavica* | -96.81 | 15.74 |
| *Cordia curassavica* | -96.81 | 15.74 |
| *Cordia curassavica* | -89.53 | 14.94 |
| *Cordia curassavica* | -66.52 | 17.58 |
| *Cordia curassavica* | -41.06 | -13.94 |
| *Cordia curassavica* | -40.74 | -19.76 |
| *Cordia curassavica* | -110.98 | 23.48 |
| *Cordia curassavica* | -52.30 | 4.94 |
| *Cordia curassavica* | -95.97 | 15.85 |
| *Cordia curassavica* | -37.54 | -9.70 |
| *Cordia curassavica* | -37.56 | -9.70 |
| *Cordia curassavica* | -96.95 | 17.73 |
| *Cordia curassavica* | -88.70 | 17.60 |
| *Cordia curassavica* | -95.97 | 15.84 |
| *Cordia curassavica* | -96.94 | 17.72 |
| *Cordia curassavica* | -37.51 | -9.72 |
| *Cordia curassavica* | -95.99 | 15.85 |
| *Cordia curassavica* | -100.93 | 18.71 |
| *Cordia curassavica* | -100.92 | 18.70 |
| *Cordia curassavica* | -96.95 | 17.71 |
| *Cordia curassavica* | -96.97 | 17.68 |
| *Cordia curassavica* | -96.96 | 17.69 |
| *Cordia curassavica* | -96.96 | 17.69 |
| *Cordia curassavica* | -96.99 | 17.79 |
| *Cordia curassavica* | -52.27 | 4.91 |
| *Cordia curassavica* | -43.27 | -11.55 |
| *Cordia curassavica* | -97.03 | 17.95 |
| *Cordia curassavica* | -95.98 | 15.84 |
| *Cordia curassavica* | -46.73 | -23.98 |
| *Cordia curassavica* | -40.38 | -10.69 |
| *Cordia curassavica* | -95.95 | 15.83 |
| *Cordia curassavica* | -84.13 | 9.40 |
| *Cordia curassavica* | -90.51 | 19.79 |
| *Cordia curassavica* | -95.84 | 15.87 |
| *Cordia curassavica* | -95.84 | 15.87 |
| *Cordia curassavica* | -96.97 | 17.68 |
| *Cordia curassavica* | -96.95 | 17.71 |
| *Cordia curassavica* | -96.21 | 17.25 |
| *Cordia curassavica* | -77.14 | 8.40 |
| *Cordia curassavica* | -45.27 | -11.04 |
| *Cordia curassavica* | -95.92 | 16.51 |
| *Cordia curassavica* | -82.81 | 9.73 |
| *Cordia curassavica* | -96.02 | 15.90 |
| *Cordia curassavica* | -95.95 | 15.83 |
| *Cordia curassavica* | -95.98 | 15.84 |
| *Cordia curassavica* | -90.49 | 19.79 |
| *Cordia curassavica* | -95.98 | 15.85 |
| *Cordia curassavica* | -95.98 | 15.84 |
| *Cordia curassavica* | -39.51 | -18.02 |
| *Cordia curassavica* | -95.95 | 15.84 |
| *Cordia curassavica* | -95.95 | 15.83 |
| *Cordia curassavica* | -39.10 | -12.67 |
| *Cordia curassavica* | -95.97 | 15.84 |
| *Cordia curassavica* | -40.89 | -18.76 |
| *Cordia curassavica* | -82.62 | 9.62 |
| *Cordia curassavica* | -111.03 | 25.45 |
| *Cordia curassavica* | -36.25 | -9.83 |
| *Cordia curassavica* | -110.93 | 24.83 |
| *Cordia curassavica* | -111.06 | 27.96 |
| *Cordia curassavica* | -88.87 | 13.74 |
| *Cordia curassavica* | -41.33 | -12.75 |
| *Cordia curassavica* | -35.81 | -9.25 |
| *Cordia curassavica* | -82.60 | 9.60 |
| *Cordia curassavica* | -41.32 | -10.37 |
| *Cordia curassavica* | -41.32 | -10.37 |
| *Cordia curassavica* | -41.46 | -16.00 |
| *Cordia curassavica* | -101.15 | 18.24 |
| *Cordia curassavica* | -53.80 | 5.68 |
| *Cordia curassavica* | -101.91 | 18.25 |
| *Cordia curassavica* | -101.81 | 18.51 |
| *Cordia curassavica* | -101.82 | 18.51 |
| *Cordia curassavica* | -101.68 | 18.33 |
| *Cordia curassavica* | -101.84 | 18.54 |
| *Cordia curassavica* | -101.83 | 18.52 |
| *Cordia curassavica* | -101.72 | 18.40 |
| *Cordia curassavica* | -111.14 | 28.03 |
| *Cordia curassavica* | -101.13 | 18.26 |
| *Cordia curassavica* | -101.15 | 18.25 |
| *Cordia curassavica* | -101.62 | 18.43 |
| *Cordia curassavica* | -101.65 | 18.42 |
| *Cordia curassavica* | -101.82 | 18.52 |
| *Cordia curassavica* | -101.62 | 18.43 |
| *Cordia curassavica* | -95.97 | 15.82 |
| *Cordia curassavica* | -63.47 | -18.14 |
| *Cordia curassavica* | -56.72 | 5.80 |
| *Cordia curassavica* | -55.23 | 5.80 |
| *Cordia curassavica* | -99.49 | 17.63 |
| *Cordia curassavica* | -108.04 | 24.77 |
| *Cordia curassavica* | -51.31 | -20.93 |
| *Cordia curassavica* | -71.71 | 6.46 |
| *Cordia curassavica* | -100.96 | 18.27 |
| *Cordia curassavica* | -51.76 | -31.23 |
| *Cordia curassavica* | -96.30 | 16.30 |
| *Cordia curassavica* | -42.87 | -15.16 |
| *Cordia curassavica* | -75.13 | 6.12 |
| *Cordia curassavica* | -39.86 | -12.76 |
| *Cordia curassavica* | -48.33 | -25.31 |
| *Cordia curassavica* | -47.92 | -24.88 |
| *Cordia curassavica* | -89.43 | 18.08 |
| *Cordia curassavica* | -105.04 | 19.49 |
| *Cordia curassavica* | -53.91 | 5.73 |
| *Cordia curassavica* | -38.98 | -12.21 |
| *Cordia curassavica* | -40.08 | -18.91 |
| *Cordia curassavica* | -91.14 | 18.01 |
| *Cordia curassavica* | -90.82 | 17.90 |
| *Cordia curassavica* | -90.74 | 17.96 |
| *Cordia curassavica* | -59.70 | 7.37 |
| *Cordia curassavica* | -110.38 | 24.57 |
| *Cordia curassavica* | -55.67 | -27.47 |
| *Cordia curassavica* | -88.13 | 17.78 |
| *Cordia curassavica* | -111.21 | 28.04 |
| *Cordia curassavica* | -52.81 | 5.32 |
| *Cordia curassavica* | -52.82 | 5.25 |
| *Cordia curassavica* | -37.82 | -8.91 |
| *Cordia curassavica* | -64.29 | -20.78 |
| *Cordia curassavica* | -99.07 | 18.40 |
| *Cordia curassavica* | -99.07 | 18.40 |
| *Cordia curassavica* | -99.12 | 18.44 |
| *Cordia curassavica* | -90.38 | 19.94 |
| *Cordia curassavica* | -112.05 | 25.85 |
| *Cordia curassavica* | -99.05 | 18.38 |
| *Cordia curassavica* | -111.52 | 25.93 |
| *Cordia curassavica* | -98.96 | 18.56 |
| *Cordia curassavica* | -99.06 | 18.38 |
| *Cordia curassavica* | -86.90 | 20.80 |
| *Cordia curassavica* | -37.82 | -8.90 |
| *Cordia curassavica* | -57.87 | -21.70 |
| *Cordia curassavica* | -52.30 | 4.93 |
| *Cordia curassavica* | -89.07 | 21.23 |
| *Cordia curassavica* | -38.70 | -10.93 |
| *Cordia curassavica* | -53.81 | 5.59 |
| *Cordia curassavica* | -99.77 | 17.87 |
| *Cordia curassavica* | -47.54 | -25.01 |
| *Cordia curassavica* | -82.75 | 9.65 |
| *Cordia curassavica* | -82.77 | 9.64 |
| *Cordia curassavica* | -36.42 | -9.25 |
| *Cordia curassavica* | -88.95 | 16.10 |
| *Cordia curassavica* | -39.86 | -18.72 |
| *Cordia curassavica* | -47.25 | -24.55 |
| *Cordia curassavica* | -83.42 | 14.17 |
| *Cordia curassavica* | -83.78 | 12.47 |
| *Cordia curassavica* | -101.80 | 17.98 |
| *Cordia curassavica* | -43.33 | -23.00 |
| *Cordia curassavica* | -53.95 | 5.74 |
| *Cordia curassavica* | -83.67 | 12.25 |
| *Cordia curassavica* | -96.23 | 15.69 |
| *Cordia curassavica* | -88.32 | 17.88 |
| *Cordia curassavica* | -52.32 | 4.94 |
| *Cordia curassavica* | -96.17 | 15.75 |
| *Cordia curassavica* | -96.24 | 15.69 |
| *Cordia curassavica* | -83.17 | 12.20 |
| *Cordia curassavica* | -110.95 | 18.77 |
| *Cordia curassavica* | -97.67 | 18.10 |
| *Cordia curassavica* | -40.23 | -14.13 |
| *Cordia curassavica* | -97.63 | 18.32 |
| *Cordia curassavica* | -97.45 | 18.33 |
| *Cordia curassavica* | -110.92 | 24.75 |
| *Cordia curassavica* | -98.84 | 18.32 |
| *Cordia curassavica* | -92.20 | 16.10 |
| *Cordia curassavica* | -83.53 | 10.50 |
| *Cordia curassavica* | -109.60 | 26.72 |
| *Cordia curassavica* | -109.60 | 26.72 |
| *Cordia curassavica* | -97.30 | 18.52 |
| *Cordia curassavica* | -83.50 | 10.53 |
| *Cordia curassavica* | -83.51 | 10.53 |
| *Cordia curassavica* | -96.95 | 17.82 |
| *Cordia curassavica* | -83.53 | 10.59 |
| *Cordia curassavica* | -97.22 | 18.23 |
| *Cordia curassavica* | -97.53 | 18.15 |
| *Cordia curassavica* | -57.67 | -21.70 |
| *Cordia curassavica* | -83.52 | 10.52 |
| *Cordia curassavica* | -83.51 | 10.51 |
| *Cordia curassavica* | -99.84 | 20.82 |
| *Cordia curassavica* | -99.12 | 21.13 |
| *Cordia curassavica* | -88.37 | 17.40 |
| *Cordia curassavica* | -88.12 | 20.52 |
| *Cordia curassavica* | -105.18 | 22.43 |
| *Cordia curassavica* | -88.05 | 20.45 |
| *Cordia curassavica* | -56.78 | -18.82 |
| *Cordia curassavica* | -109.58 | 26.12 |
| *Cordia curassavica* | -39.80 | -19.58 |
| *Cordia curassavica* | -93.69 | 16.87 |
| *Cordia curassavica* | -97.47 | 18.33 |
| *Cordia curassavica* | -40.35 | -14.52 |
| *Cordia curassavica* | -110.92 | 18.75 |
| *Cordia curassavica* | -58.42 | 6.83 |
| *Cordia curassavica* | -96.35 | 16.56 |
| *Cordia curassavica* | -88.03 | 20.42 |
| *Cordia curassavica* | -52.47 | 4.30 |
| *Cordia curassavica* | -97.88 | 17.33 |
| *Cordia curassavica* | -102.54 | 20.13 |
| *Cordia curassavica* | -102.50 | 19.38 |
| *Cordia curassavica* | -95.50 | 16.32 |
| *Cordia curassavica* | -95.43 | 16.07 |
| *Cordia curassavica* | -95.43 | 16.03 |
| *Cordia curassavica* | -95.48 | 16.34 |
| *Cordia curassavica* | -90.08 | 20.48 |
| *Cordia curassavica* | -88.03 | 20.42 |
| *Cordia curassavica* | -48.61 | -27.09 |
| *Cordia curassavica* | -84.16 | 9.40 |
| *Cordia curassavica* | -84.17 | 9.40 |
| *Cordia curassavica* | -97.23 | 18.30 |
| *Cordia curassavica* | -97.18 | 18.15 |
| *Cordia curassavica* | -52.26 | 4.91 |
| *Cordia curassavica* | -56.65 | -18.98 |
| *Cordia curassavica* | -41.28 | -13.81 |
| *Cordia curassavica* | -97.43 | 18.47 |
| *Cordia curassavica* | -98.22 | 16.38 |
| *Cordia curassavica* | -95.92 | 16.50 |
| *Cordia curassavica* | -76.94 | 8.01 |
| *Cordia curassavica* | -90.24 | 20.51 |
| *Cordia curassavica* | -104.68 | 21.58 |
| *Cordia curassavica* | -83.50 | 10.51 |
| *Cordia curassavica* | -52.33 | 4.93 |
| *Cordia curassavica* | -83.50 | 10.52 |
| *Cordia curassavica* | -56.57 | -18.07 |
| *Cordia curassavica* | -83.58 | 10.78 |
| *Cordia curassavica* | -97.68 | 18.08 |
| *Cordia curassavica* | -97.72 | 17.72 |
| *Cordia curassavica* | -87.60 | 19.78 |
| *Cordia curassavica* | -95.23 | 16.61 |
| *Cordia curassavica* | -99.61 | 16.97 |
| *Cordia curassavica* | -84.16 | 9.39 |
| *Cordia curassavica* | -95.45 | 16.31 |
| *Cordia curassavica* | -95.18 | 16.55 |
| *Cordia curassavica* | -95.21 | 16.58 |
| *Cordia curassavica* | -83.59 | 10.79 |
| *Cordia curassavica* | -97.27 | 18.73 |
| *Cordia curassavica* | -97.27 | 18.73 |
| *Cordia curassavica* | -58.15 | 6.78 |
| *Cordia curassavica* | -90.20 | 20.77 |
| *Cordia curassavica* | -94.93 | 16.55 |
| *Cordia curassavica* | -96.62 | 16.88 |
| *Cordia curassavica* | -94.95 | 16.55 |
| *Cordia curassavica* | -56.78 | -19.07 |
| *Cordia curassavica* | -57.92 | 6.63 |
| *Cordia curassavica* | -58.18 | 6.67 |
| *Cordia curassavica* | -52.90 | 5.40 |
| *Cordia curassavica* | -40.34 | -20.32 |
| *Cordia curassavica* | -40.30 | -20.28 |
| *Cordia curassavica* | -97.20 | 18.22 |
| *Cordia curassavica* | -53.67 | 5.63 |
| *Cordia curassavica* | -95.45 | 16.31 |
| *Cordia curassavica* | -110.24 | 23.45 |
| *Cordia curassavica* | -111.05 | 27.97 |
| *Cordia curassavica* | -111.90 | 26.33 |
| *Cordia curassavica* | -87.60 | 19.78 |
| *Cordia curassavica* | -107.99 | 24.72 |
| *Cordia curassavica* | -111.05 | 27.97 |
| *Cordia curassavica* | -40.68 | -19.81 |
| *Cordia curassavica* | -86.75 | 21.26 |
| *Cordia curassavica* | -40.60 | -19.91 |
| *Cordia curassavica* | -86.98 | 20.28 |
| *Cordia curassavica* | -52.59 | 5.28 |
| *Cordia curassavica* | -91.54 | 17.80 |
| *Cordia curassavica* | -40.12 | -12.72 |
| *Cordia curassavica* | -39.93 | -12.70 |
| *Cordia curassavica* | -40.62 | -19.94 |
| *Cordia curassavica* | -40.62 | -19.94 |
| *Cordia curassavica* | -40.64 | -19.93 |
| *Cordia curassavica* | -40.51 | -19.94 |
| *Cordia curassavica* | -38.97 | -12.25 |
| *Cordia curassavica* | -40.45 | -19.93 |
| *Cordia curassavica* | -95.32 | 16.12 |
| *Cordia curassavica* | -95.36 | 16.34 |
| *Cordia curassavica* | -39.65 | -17.52 |
| *Cordia curassavica* | -69.95 | 9.60 |
| *Cordia curassavica* | -99.63 | 17.81 |
| *Cordia curassavica* | -39.65 | -17.52 |
| *Cordia curassavica* | -93.18 | 18.41 |
| *Cordia curassavica* | -101.32 | 17.92 |
| *Cordia curassavica* | -88.25 | 20.70 |
| *Cordia curassavica* | -92.42 | 14.75 |
| *Cordia curassavica* | -82.70 | 9.63 |
| *Cordia curassavica* | -100.67 | 18.36 |
| *Cordia curassavica* | -96.63 | 18.62 |
| *Cordia curassavica* | -40.28 | -20.28 |
| *Cordia curassavica* | -87.08 | 19.80 |
| *Cordia curassavica* | -56.65 | -19.98 |
| *Cordia curassavica* | -53.00 | 5.42 |
| *Cordia curassavica* | -96.28 | 16.68 |
| *Cordia curassavica* | -102.25 | 18.36 |
| *Cordia curassavica* | -73.00 | 8.00 |
| *Cordia curassavica* | -101.80 | 18.76 |
| *Cordia curassavica* | -96.39 | 16.87 |
| *Cordia curassavica* | -96.73 | 16.42 |
| *Cordia curassavica* | -97.05 | 18.08 |
| *Cordia curassavica* | -96.52 | 17.03 |
| *Cordia curassavica* | -46.12 | -23.75 |
| *Cordia curassavica* | -97.53 | 18.07 |
| *Cordia curassavica* | -87.70 | 20.50 |
| *Cordia curassavica* | -87.97 | 19.70 |
| *Cordia curassavica* | -88.00 | 19.58 |
| *Cordia curassavica* | -105.04 | 19.50 |
| *Cordia curassavica* | -79.55 | 8.98 |
| *Cordia curassavica* | -105.05 | 19.50 |
| *Cordia curassavica* | -92.16 | 18.83 |
| *Cordia curassavica* | -97.00 | 17.75 |
| *Cordia curassavica* | -90.30 | 18.62 |
| *Cordia curassavica* | -88.15 | 21.12 |
| *Cordia curassavica* | -88.22 | 20.70 |
| *Cordia curassavica* | -88.58 | 20.30 |
| *Cordia curassavica* | -88.23 | 20.70 |
| *Cordia curassavica* | -88.83 | 17.97 |
| *Cordia curassavica* | -91.62 | 17.93 |
| *Cordia curassavica* | -71.16 | 6.91 |
| *Cordia curassavica* | -42.82 | -22.92 |
| *Cordia curassavica* | -40.33 | -20.43 |
| *Cordia curassavica* | -52.28 | 4.93 |
| *Cordia curassavica* | -88.50 | 19.67 |
| *Cordia curassavica* | -88.23 | 18.68 |
| *Cordia curassavica* | -98.47 | 17.95 |
| *Cordia curassavica* | -97.72 | 17.85 |
| *Cordia curassavica* | -99.79 | 18.50 |
| *Cordia curassavica* | -90.38 | 19.92 |
| *Cordia curassavica* | -98.66 | 17.74 |
| *Cordia curassavica* | -101.18 | 18.83 |
| *Cordia curassavica* | -89.07 | 20.48 |
| *Cordia curassavica* | -101.97 | 18.60 |
| *Cordia curassavica* | -102.06 | 18.79 |
| *Cordia curassavica* | -98.58 | 17.55 |
| *Cordia curassavica* | -98.57 | 17.53 |
| *Cordia curassavica* | -68.58 | -15.38 |
| *Cordia curassavica* | -67.66 | 9.82 |
| *Cordia curassavica* | -88.15 | 18.95 |
| *Cordia curassavica* | -88.52 | 18.42 |
| *Cordia curassavica* | -88.52 | 18.48 |
| *Cordia curassavica* | -97.60 | 17.70 |
| *Cordia curassavica* | -72.10 | 12.17 |
| *Cordia curassavica* | -63.69 | 10.64 |
| *Cordia curassavica* | -40.51 | -20.66 |
| *Cordia curassavica* | -88.92 | 20.50 |
| *Cordia curassavica* | -63.95 | 10.62 |
| *Cordia curassavica* | -88.90 | 20.45 |
| *Cordia curassavica* | -89.52 | 20.42 |
| *Cordia curassavica* | -68.90 | 18.42 |
| *Cordia curassavica* | -100.94 | 18.67 |
| *Cordia curassavica* | -97.40 | 18.70 |
| *Cordia curassavica* | -83.87 | 10.00 |
| *Cordia curassavica* | -68.58 | -15.38 |
| *Cordia curassavica* | -43.10 | -22.89 |
| *Cordia curassavica* | -70.90 | 18.30 |
| *Cordia curassavica* | -39.08 | -12.53 |
| *Cordia curassavica* | -88.92 | 20.49 |
| *Cordia curassavica* | -87.73 | 20.55 |
| *Cordia curassavica* | -40.26 | -11.23 |
| *Cordia curassavica* | -88.38 | 20.78 |
| *Cordia curassavica* | -88.38 | 18.75 |
| *Cordia curassavica* | -88.30 | 18.50 |
| *Cordia curassavica* | -72.41 | 11.42 |
| *Cordia curassavica* | -89.07 | 18.03 |
| *Cordia curassavica* | -40.29 | -20.33 |
| *Cordia curassavica* | -86.93 | 20.82 |
| *Cordia curassavica* | -40.08 | -13.86 |
| *Cordia curassavica* | -97.40 | 18.47 |
| *Cordia curassavica* | -99.81 | 16.83 |
| *Cordia curassavica* | -39.55 | -17.02 |
| *Cordia curassavica* | -95.23 | 16.30 |
| *Cordia curassavica* | -73.91 | 7.01 |
| *Cordia curassavica* | -86.05 | 11.48 |
| *Cordia curassavica* | -86.05 | 11.48 |
| *Cordia curassavica* | -72.44 | 7.90 |
| *Cordia curassavica* | -53.95 | 5.74 |
| *Cordia curassavica* | -83.33 | 14.08 |
| *Cordia curassavica* | -66.88 | 10.50 |
| *Cordia curassavica* | -97.43 | 18.37 |
| *Cordia curassavica* | -89.60 | 21.10 |
| *Cordia curassavica* | -39.63 | -14.70 |
| *Cordia curassavica* | -69.27 | 11.32 |
| *Cordia curassavica* | -82.98 | 9.88 |
| *Cordia curassavica* | -40.43 | -13.44 |
| *Cordia curassavica* | -57.00 | -27.00 |
| *Cordia curassavica* | -39.18 | -17.10 |
| *Cordia curassavica* | -39.42 | -16.88 |
| *Cordia curassavica* | -39.18 | -17.10 |
| *Cordia curassavica* | -52.95 | 5.39 |
| *Cordia curassavica* | -39.42 | -16.88 |
| *Cordia curassavica* | -41.20 | -12.85 |
| *Cordia curassavica* | -46.79 | -24.18 |
| *Cordia curassavica* | -47.92 | -25.01 |
| *Cordia curassavica* | -82.91 | 9.85 |
| *Cordia curassavica* | -97.15 | 18.27 |
| *Cordia curassavica* | -48.67 | -26.67 |
| *Cordia curassavica* | -62.23 | 9.98 |
| *Cordia curassavica* | -73.76 | 7.16 |
| *Cordia curassavica* | -49.00 | -22.00 |
| *Cordia curassavica* | -77.68 | 8.18 |
| *Cordia curassavica* | -97.05 | 18.07 |
| *Cordia curassavica* | -52.35 | 4.92 |
| *Cordia curassavica* | -41.22 | -13.33 |
| *Cordia curassavica* | -39.55 | -10.67 |
| *Cordia curassavica* | -41.22 | -13.33 |
| *Cordia curassavica* | -38.99 | -14.28 |
| *Cordia curassavica* | -38.92 | -15.92 |
| *Cordia curassavica* | -39.08 | -16.43 |
| *Cordia curassavica* | -39.55 | -10.67 |
| *Cordia curassavica* | -38.98 | -14.30 |
| *Cordia curassavica* | -39.08 | -16.43 |
| *Cordia curassavica* | -51.14 | -30.18 |
| *Cordia curassavica* | -37.15 | -10.69 |
| *Cordia curassavica* | -41.11 | -20.85 |
| *Cordia curassavica* | -40.75 | -20.00 |
| *Cordia curassavica* | -46.90 | -13.26 |
| *Cordia curassavica* | -49.36 | -28.93 |
| *Cordia curassavica* | -43.68 | -18.42 |
| *Cordia curassavica* | -39.76 | -18.74 |
| *Cordia curassavica* | -38.77 | -12.05 |
| *Cordia curassavica* | -41.77 | -12.42 |
| *Cordia curassavica* | -96.67 | 19.33 |
| *Cordia curassavica* | -51.02 | -30.28 |
| *Cordia curassavica* | -51.19 | -30.03 |
| *Cordia curassavica* | -82.57 | 8.87 |
| *Cordia curassavica* | -48.60 | -15.57 |
| *Cordia curassavica* | -53.77 | 5.66 |
| *Cordia curassavica* | -40.47 | -11.85 |
| *Cordia curassavica* | -54.26 | -24.08 |
| *Cordia curassavica* | -77.73 | 8.94 |
| *Cordia curassavica* | -79.92 | 8.69 |
| *Cordia curassavica* | -79.31 | 9.24 |
| *Cordia curassavica* | -79.90 | 8.54 |
| *Cordia curassavica* | -80.94 | 8.09 |
| *Cordia curassavica* | -80.70 | 7.78 |
| *Cordia curassavica* | -80.33 | 7.58 |
| *Cordia curassavica* | -48.51 | -25.52 |
| *Cordia curassavica* | -52.65 | 5.15 |
| *Cordia curassavica* | -43.04 | -22.66 |
| *Cordia curassavica* | -52.33 | 4.94 |
| *Cordia curassavica* | -47.88 | -25.33 |
| *Cordia curassavica* | -47.88 | -25.03 |
| *Cordia curassavica* | -39.34 | -14.99 |
| *Cordia curassavica* | -46.40 | -24.00 |
| *Cordia curassavica* | -79.38 | 9.08 |
| *Cordia curassavica* | -43.21 | -22.91 |
| *Cordia curassavica* | -97.12 | 18.20 |
| *Cordia curassavica* | -97.13 | 18.30 |
| *Cordia curassavica* | -74.50 | 10.00 |
| *Cordia curassavica* | -48.60 | -25.85 |
| *Cordia curassavica* | -38.70 | -12.53 |
| *Cordia curassavica* | -111.00 | 18.75 |
| *Cordia curassavica* | -46.78 | -24.18 |
| *Cordia curassavica* | -48.67 | -27.65 |
| *Cordia curassavica* | -111.49 | 25.97 |
| *Cordia curassavica* | -74.97 | 4.20 |
| *Cordia curassavica* | -50.13 | -29.99 |
| *Cordia curassavica* | -63.25 | -17.75 |
| *Cordia curassavica* | -97.37 | 18.47 |
| *Cordia curassavica* | -96.95 | 17.80 |
| *Cordia curassavica* | -43.85 | -19.99 |
| *Cordia curassavica* | -72.87 | 3.88 |
| *Cordia curassavica* | -75.39 | 10.31 |
| *Cordia curassavica* | -72.50 | 5.50 |
| *Cordia curassavica* | -100.90 | 18.23 |
| *Cordia curassavica* | -75.00 | 10.75 |
| *Cordia curassavica* | -42.53 | -22.29 |
| *Cordia curassavica* | -42.10 | -22.84 |
| *Cordia curassavica* | -79.10 | 9.17 |
| *Cordia curassavica* | -102.53 | 23.95 |
| *Cordia curassavica* | -43.01 | -22.53 |
| *Cordia curassavica* | -82.25 | 8.18 |
| *Cordia curassavica* | -56.00 | -27.00 |
| *Cordia curassavica* | -88.68 | 17.22 |
| *Cordia curassavica* | -52.87 | -10.83 |
| *Cordia curassavica* | -65.91 | 7.08 |
| *Cordia curassavica* | -96.93 | 17.68 |
| *Cordia curassavica* | -41.37 | -13.01 |
| *Cordia curassavica* | -55.00 | -10.00 |
| *Cordia curassavica* | -56.48 | -22.10 |
| *Cordia curassavica* | -64.44 | -16.73 |
| *Cordia curassavica* | -72.71 | 6.61 |
| *Cordia curassavica* | -55.79 | -32.87 |
| *Cordia curassavica* | -58.05 | -28.03 |
| *Cordia curassavica* | -64.22 | -24.23 |
| *Cordia curassavica* | -51.75 | -31.22 |
| *Cordia curassavica* | -38.70 | -10.93 |
| *Cordia curassavica* | -78.19 | -1.46 |
| *Cordia curassavica* | -41.85 | -13.23 |
| *Cordia curassavica* | -40.17 | -14.00 |
| *Cordia curassavica* | -58.70 | 4.74 |
| *Cordia curassavica* | -77.50 | 18.25 |
| *Cordia curassavica* | -72.75 | 6.57 |
| *Cordia curassavica* | -73.25 | 6.70 |
| *Cordia curassavica* | -40.11 | -13.94 |
| *Cordia curassavica* | -55.58 | -27.33 |
| *Cordia curassavica* | -52.97 | 3.86 |
| *Cordia curassavica* | -55.63 | 4.10 |
| *Cordia curassavica* | -72.74 | 6.59 |
| *Cordia curassavica* | -38.02 | -9.56 |
| *Cordia curassavica* | -64.92 | -35.39 |
| *Cordia curassavica* | -72.74 | 6.59 |
| *Cordia curassavica* | -41.80 | -13.28 |
| *Cordia curassavica* | -55.57 | -27.50 |
| *Cordia curassavica* | -73.20 | 6.72 |
| *Cordia curassavica* | -64.81 | -23.78 |
| *Cordia curassavica* | -74.14 | -9.18 |
| *Cordia curassavica* | -39.83 | -12.89 |
| *Cordia curassavica* | -41.51 | -12.87 |
| *Cordia curassavica* | -72.74 | 6.59 |
| *Cordia curassavica* | -73.24 | 6.70 |
| *Cordia curassavica* | -73.24 | 6.71 |
| *Cordia curassavica* | -39.54 | -15.16 |
| *Cordia curassavica* | -42.02 | -22.88 |
| *Cordia curassavica* | -73.24 | 6.70 |
| *Cordia inermis* | -106.13 | 23.23 |
| *Cordia inermis* | -96.77 | 19.42 |
| *Cordia inermis* | -96.90 | 19.35 |
| *Cordia inermis* | -96.75 | 19.23 |
| *Cordia inermis* | -96.48 | 19.27 |
| *Cordia inermis* | -96.91 | 19.36 |
| *Cordia inermis* | -96.86 | 19.61 |
| *Cordia inermis* | -96.48 | 19.33 |
| *Cordia inermis* | -86.28 | 13.39 |
| *Cordia inermis* | -99.93 | 16.91 |
| *Cordia inermis* | -95.01 | 16.66 |
| *Cordia inermis* | -95.04 | 16.70 |
| *Cordia inermis* | -95.01 | 16.67 |
| *Cordia inermis* | -95.01 | 16.68 |
| *Cordia inermis* | -105.06 | 19.51 |
| *Cordia inermis* | -105.04 | 19.50 |
| *Cordia inermis* | -105.04 | 19.50 |
| *Cordia inermis* | -105.04 | 19.50 |
| *Cordia inermis* | -96.20 | 15.78 |
| *Cordia inermis* | -106.47 | 23.23 |
| *Cordia inermis* | -106.71 | 24.41 |
| *Cordia inermis* | -106.59 | 24.40 |
| *Cordia inermis* | -100.19 | 19.01 |
| *Cordia inermis* | -96.66 | 19.36 |
| *Cordia inermis* | -95.38 | 16.37 |
| *Cordia inermis* | -102.27 | 18.18 |
| *Cordia inermis* | -104.97 | 19.42 |
| *Cordia inermis* | -95.57 | 16.41 |
| *Cordia inermis* | -93.67 | 16.67 |
| *Cordia inermis* | -100.63 | 17.26 |
| *Cordia inermis* | -95.49 | 16.70 |
| *Cordia inermis* | -100.38 | 19.44 |
| *Cordia inermis* | -102.28 | 18.28 |
| *Cordia inermis* | -93.05 | 17.19 |
| *Cordia inermis* | -96.90 | 18.86 |
| *Cordia inermis* | -104.32 | 19.69 |
| *Cordia inermis* | -104.08 | 19.66 |
| *Cordia inermis* | -104.29 | 19.80 |
| *Cordia inermis* | -104.01 | 19.94 |
| *Cordia inermis* | -104.01 | 19.67 |
| *Cordia inermis* | -104.37 | 19.70 |
| *Cordia inermis* | -103.86 | 19.36 |
| *Cordia inermis* | -95.36 | 16.34 |
| *Cordia inermis* | -95.66 | 16.54 |
| *Cordia inermis* | -95.43 | 16.32 |
| *Cordia inermis* | -95.42 | 16.03 |
| *Cordia inermis* | -104.89 | 21.16 |
| *Cordia inermis* | -96.78 | 19.43 |
| *Cordia inermis* | -96.56 | 19.41 |
| *Cordia inermis* | -95.93 | 15.87 |
| *Cordia inermis* | -96.73 | 19.33 |
| *Cordia inermis* | -103.33 | 20.83 |
| *Cordia inermis* | -103.79 | 19.77 |
| *Cordia inermis* | -95.46 | 15.99 |
| *Cordia inermis* | -99.60 | 16.97 |
| *Cordia inermis* | -100.63 | 17.26 |
| *Cordia inermis* | -95.97 | 15.85 |
| *Cordia inermis* | -100.37 | 18.69 |
| *Cordia inermis* | -94.16 | 16.73 |
| *Cordia inermis* | -96.49 | 19.33 |
| *Cordia inermis* | -95.76 | 16.40 |
| *Cordia inermis* | -95.48 | 16.33 |
| *Cordia inermis* | -106.54 | 21.65 |
| *Cordia inermis* | -95.87 | 15.99 |
| *Cordia inermis* | -95.24 | 16.32 |
| *Cordia inermis* | -90.38 | 19.94 |
| *Cordia inermis* | -96.46 | 19.77 |
| *Cordia inermis* | -99.88 | 18.32 |
| *Cordia inermis* | -96.83 | 19.59 |
| *Cordia inermis* | -94.16 | 16.74 |
| *Cordia inermis* | -96.68 | 19.76 |
| *Cordia inermis* | -100.03 | 16.99 |
| *Cordia inermis* | -100.29 | 19.01 |
| *Cordia inermis* | -103.13 | 18.74 |
| *Cordia inermis* | -95.38 | 16.10 |
| *Cordia inermis* | -99.80 | 16.83 |
| *Cordia inermis* | -103.58 | 18.58 |
| *Cordia inermis* | -94.97 | 16.62 |
| *Cordia inermis* | -96.03 | 15.94 |
| *Cordia inermis* | -104.77 | 20.56 |
| *Cordia inermis* | -106.42 | 23.94 |
| *Cordia inermis* | -96.76 | 19.46 |
| *Cordia inermis* | -103.49 | 20.35 |
| *Cordia inermis* | -101.53 | 17.70 |
| *Cordia inermis* | -101.04 | 17.39 |
| *Cordia inermis* | -102.88 | 18.13 |
| *Cordia inermis* | -96.75 | 19.23 |
| *Cordia inermis* | -105.93 | 23.39 |
| *Cordia inermis* | -99.48 | 17.76 |
| *Cordia inermis* | -99.89 | 16.93 |
| *Cordia inermis* | -96.77 | 19.34 |
| *Cordia inermis* | -99.71 | 16.80 |
| *Cordia inermis* | -95.87 | 15.90 |
| *Cordia inermis* | -96.78 | 19.34 |
| *Cordia inermis* | -103.67 | 20.42 |
| *Cordia inermis* | -107.17 | 24.86 |
| *Cordia inermis* | -101.55 | 17.64 |
| *Cordia inermis* | -100.45 | 18.92 |
| *Cordia inermis* | -93.85 | 16.28 |
| *Cordia inermis* | -100.18 | 18.37 |
| *Cordia inermis* | -95.68 | 16.09 |
| *Cordia inermis* | -101.91 | 18.36 |
| *Cordia inermis* | -91.43 | 17.47 |
| *Cordia inermis* | -95.45 | 16.31 |
| *Cordia inermis* | -95.99 | 15.81 |
| *Cordia inermis* | -103.25 | 19.68 |
| *Cordia inermis* | -96.57 | 19.56 |
| *Cordia inermis* | -95.76 | 15.92 |
| *Cordia inermis* | -96.93 | 18.89 |
| *Cordia inermis* | -96.56 | 19.40 |
| *Cordia inermis* | -103.22 | 18.74 |
| *Cordia inermis* | -100.03 | 16.99 |
| *Cordia inermis* | -96.66 | 19.36 |
| *Cordia inermis* | -95.97 | 15.84 |
| *Cordia inermis* | -99.76 | 17.85 |
| *Cordia inermis* | -96.15 | 18.58 |
| *Cordia inermis* | -95.97 | 15.83 |
| *Cordia inermis* | -95.96 | 15.83 |
| *Cordia inermis* | -95.96 | 15.86 |
| *Cordia inermis* | -96.22 | 15.78 |
| *Cordia inermis* | -95.98 | 15.84 |
| *Cordia inermis* | -99.90 | 16.87 |
| *Cordia inermis* | -92.63 | 15.36 |
| *Cordia inermis* | -106.32 | 23.88 |
| *Cordia inermis* | -99.19 | 21.01 |
| *Cordia inermis* | -103.84 | 20.88 |
| *Cordia inermis* | -106.61 | 24.40 |
| *Cordia inermis* | -105.06 | 19.51 |
| *Cordia inermis* | -95.87 | 15.87 |
| *Cordia inermis* | -96.53 | 19.33 |
| *Cordia inermis* | -99.93 | 16.91 |
| *Cordia inermis* | -97.08 | 20.43 |
| *Cordia inermis* | -95.66 | 16.08 |
| *Cordia inermis* | -104.48 | 21.05 |
| *Cordia inermis* | -103.93 | 20.43 |
| *Cordia inermis* | -105.04 | 19.50 |
| *Cordia inermis* | -98.95 | 16.62 |
| *Cordia inermis* | -99.12 | 16.78 |
| *Cordia inermis* | -99.78 | 16.83 |
| *Cordia inermis* | -103.35 | 20.68 |
| *Cordia inermis* | -107.38 | 24.80 |
| *Cordia inermis* | -96.27 | 15.76 |
| *Cordia inermis* | -96.00 | 15.87 |
| *Cordia inermis* | -105.07 | 19.53 |
| *Cordia inermis* | -96.02 | 15.93 |
| *Cordia inermis* | -104.98 | 19.44 |
| *Cordia inermis* | -102.25 | 18.22 |
| *Cordia inermis* | -96.76 | 19.36 |
| *Cordia inermis* | -102.92 | 20.47 |
| *Cordia inermis* | -100.24 | 16.99 |
| *Cordia inermis* | -101.45 | 17.59 |
| *Cordia inermis* | -99.74 | 16.80 |
| *Cordia inermis* | -95.44 | 16.31 |
| *Cordia inermis* | -90.32 | 19.93 |
| *Cordia inermis* | -103.22 | 18.73 |
| *Cordia inermis* | -90.39 | 19.93 |
| *Cordia inermis* | -91.42 | 17.51 |
| *Cordia inermis* | -95.39 | 16.38 |
| *Cordia inermis* | -97.08 | 19.14 |
| *Cordia inermis* | -96.49 | 19.49 |
| *Cordia inermis* | -96.18 | 19.25 |
| *Cordia inermis* | -95.43 | 16.07 |
| *Cordia inermis* | -96.44 | 19.28 |
| *Cordia inermis* | -101.27 | 17.54 |
| *Cordia inermis* | -96.69 | 19.45 |
| *Cordia inermis* | -105.93 | 23.39 |
| *Cordia inermis* | -96.75 | 19.23 |
| *Cordia inermis* | -96.81 | 19.56 |
| *Cordia inermis* | -96.83 | 19.57 |
| *Cordia inermis* | -96.57 | 19.56 |
| *Cordia inermis* | -96.76 | 19.46 |
| *Cordia inermis* | -97.13 | 18.82 |
| *Cordia inermis* | -96.83 | 19.42 |
| *Cordia inermis* | -96.44 | 19.28 |
| *Cordia inermis* | -96.93 | 19.34 |
| *Cordia inermis* | -96.80 | 19.42 |
| *Cordia inermis* | -96.78 | 19.49 |
| *Cordia inermis* | -96.69 | 19.79 |
| *Cordia inermis* | -104.10 | 20.28 |
| *Cordia inermis* | -96.77 | 19.36 |
| *Cordia inermis* | -96.56 | 19.40 |
| *Cordia inermis* | -96.49 | 19.28 |
| *Cordia inermis* | -97.11 | 19.70 |
| *Cordia inermis* | -96.53 | 19.76 |
| *Cordia inermis* | -83.43 | 10.08 |
| *Cordia inermis* | -72.25 | 7.95 |
| *Cordia inermis* | -86.79 | 13.91 |
| *Cordia inermis* | -88.93 | 13.60 |
| *Cordia inermis* | -85.78 | 10.92 |
| *Cordia inermis* | -85.64 | 10.65 |
| *Cordia inermis* | -88.93 | 13.88 |
| *Cordia inermis* | -85.58 | 10.88 |
| *Cordia inermis* | -96.81 | 15.74 |
| *Cordia inermis* | -96.27 | 15.76 |
| *Cordia inermis* | -84.90 | 9.76 |
| *Cordia inermis* | -84.90 | 9.78 |
| *Cordia inermis* | -85.35 | 9.85 |
| *Cordia inermis* | -84.86 | 10.10 |
| *Cordia inermis* | -106.55 | 21.63 |
| *Cordia inermis* | -84.71 | 9.90 |
| *Cordia inermis* | -85.32 | 10.38 |
| *Cordia inermis* | -85.11 | 10.08 |
| *Cordia inermis* | -83.00 | 9.00 |
| *Cordia inermis* | -83.18 | 9.05 |
| *Cordia inermis* | -83.34 | 8.40 |
| *Cordia inermis* | -96.80 | 19.42 |
| *Cordia inermis* | -96.67 | 19.33 |
| *Cordia inermis* | -97.04 | 19.60 |
| *Cordia inermis* | -96.96 | 19.17 |
| *Cordia inermis* | -96.77 | 19.33 |
| *Cordia inermis* | -85.75 | 10.27 |
| *Cordia inermis* | -85.73 | 10.90 |
| *Cordia inermis* | -86.72 | 12.08 |
| *Cordia inermis* | -86.33 | 13.02 |
| *Cordia inermis* | -86.32 | 12.10 |
| *Cordia inermis* | -86.40 | 13.68 |
| *Cordia inermis* | -86.52 | 11.97 |
| *Cordia inermis* | -86.32 | 13.68 |
| *Cordia inermis* | -86.27 | 13.38 |
| *Cordia inermis* | 0.00 | 0.00 |
| *Cordia inermis* | -86.62 | 14.82 |
| *Cordia inermis* | -84.75 | 13.25 |
| *Cordia inermis* | -106.59 | 24.40 |
| *Cordia inermis* | -106.71 | 24.41 |
| *Cordia inermis* | -106.47 | 23.24 |
| *Cordia inermis* | -87.94 | 19.61 |
| *Cordia inermis* | -96.20 | 15.78 |
| *Cordia inermis* | -105.04 | 19.50 |
| *Cordia inermis* | -105.05 | 19.50 |
| *Cordia inermis* | -105.04 | 19.50 |
| *Cordia inermis* | -105.04 | 19.50 |
| *Cordia inermis* | -95.66 | 16.54 |
| *Cordia inermis* | -95.43 | 16.03 |
| *Cordia inermis* | -95.46 | 16.29 |
| *Cordia inermis* | -95.36 | 16.34 |
| *Cordia inermis* | -101.27 | 17.54 |
| *Cordia inermis* | -96.48 | 19.48 |
| *Cordia inermis* | -95.43 | 16.07 |
| *Cordia inermis* | -95.38 | 16.37 |
| *Cordia inermis* | -96.44 | 19.28 |
| *Cordia inermis* | -96.18 | 19.25 |
| *Cordia inermis* | -97.07 | 19.13 |
| *Cordia inermis* | -86.27 | 13.38 |
| *Cordia inermis* | -94.87 | 18.24 |
| *Cordia inermis* | -96.76 | 19.36 |
| *Cordia inermis* | -99.81 | 16.83 |
| *Cordia inermis* | -104.10 | 20.28 |
| *Cordia inermis* | -105.92 | 23.38 |
| *Cordia inermis* | -96.48 | 19.27 |
| *Cordia inermis* | -97.07 | 18.87 |
| *Cordia inermis* | -96.55 | 19.58 |
| *Cordia inermis* | -96.56 | 19.40 |
| *Cordia inermis* | -96.82 | 19.58 |
| *Cordia inermis* | -96.70 | 19.43 |
| *Cordia inermis* | -96.90 | 19.50 |
| *Cordia inermis* | -96.77 | 19.42 |
| *Cordia inermis* | -96.45 | 19.29 |
| *Cordia inermis* | -97.11 | 19.70 |
| *Cordia inermis* | -96.76 | 19.46 |
| *Cordia inermis* | -96.77 | 19.48 |
| *Cordia inermis* | -96.75 | 19.23 |
| *Cordia inermis* | -96.68 | 19.78 |
| *Cordia inermis* | -96.83 | 19.42 |
| *Cordia inermis* | -85.33 | 10.35 |
| *Cordia inermis* | -84.73 | 10.90 |
| *Cordia inermis* | -84.70 | 9.85 |
| *Cordia inermis* | -85.72 | 10.90 |
| *Cordia inermis* | -85.36 | 10.16 |
| *Cordia inermis* | -83.73 | 10.64 |
| *Cordia inermis* | -85.37 | 10.17 |
| *Cordia inermis* | -83.33 | 8.40 |
| *Cordia inermis* | -85.78 | 10.49 |
| *Cordia inermis* | -84.34 | 9.98 |
| *Cordia inermis* | -85.35 | 10.35 |
| *Cordia inermis* | -84.66 | 10.71 |
| *Cordia inermis* | -84.73 | 9.96 |
| *Cordia inermis* | -84.90 | 9.95 |
| *Cordia inermis* | -84.96 | 10.06 |
| *Cordia inermis* | -84.73 | 9.94 |
| *Cordia inermis* | -84.73 | 9.98 |
| *Cordia inermis* | -85.61 | 10.83 |
| *Cordia inermis* | -85.66 | 10.53 |
| *Cordia spinescens* | -58.60 | 6.52 |
| *Cordia spinescens* | -56.87 | 4.75 |
| *Cordia spinescens* | -56.82 | 4.77 |
| *Cordia spinescens* | -104.91 | 21.42 |
| *Cordia spinescens* | -96.56 | 17.74 |
| *Cordia spinescens* | -96.96 | 19.51 |
| *Cordia spinescens* | -97.03 | 19.20 |
| *Cordia spinescens* | -97.44 | 20.40 |
| *Cordia spinescens* | -96.86 | 19.22 |
| *Cordia spinescens* | -96.42 | 19.76 |
| *Cordia spinescens* | -96.78 | 18.70 |
| *Cordia spinescens* | -95.21 | 18.46 |
| *Cordia spinescens* | -95.52 | 18.07 |
| *Cordia spinescens* | -96.77 | 19.48 |
| *Cordia spinescens* | -96.67 | 18.67 |
| *Cordia spinescens* | -96.30 | 19.18 |
| *Cordia spinescens* | -97.02 | 19.08 |
| *Cordia spinescens* | -94.05 | 18.27 |
| *Cordia spinescens* | -96.90 | 20.35 |
| *Cordia spinescens* | -18.28 | 18.28 |
| *Cordia spinescens* | -96.75 | 18.87 |
| *Cordia spinescens* | -97.20 | 19.96 |
| *Cordia spinescens* | -94.88 | 18.27 |
| *Cordia spinescens* | -91.10 | 16.73 |
| *Cordia spinescens* | -96.96 | 17.83 |
| *Cordia spinescens* | -75.61 | 4.71 |
| *Cordia spinescens* | -93.07 | 16.82 |
| *Cordia spinescens* | -92.61 | 15.55 |
| *Cordia spinescens* | -93.05 | 15.80 |
| *Cordia spinescens* | -91.25 | 17.03 |
| *Cordia spinescens* | -91.60 | 16.80 |
| *Cordia spinescens* | -91.32 | 17.01 |
| *Cordia spinescens* | -91.25 | 16.93 |
| *Cordia spinescens* | -91.25 | 16.93 |
| *Cordia spinescens* | -91.30 | 17.10 |
| *Cordia spinescens* | -91.29 | 16.98 |
| *Cordia spinescens* | -105.75 | 22.88 |
| *Cordia spinescens* | -97.45 | 19.88 |
| *Cordia spinescens* | -97.71 | 20.47 |
| *Cordia spinescens* | -97.62 | 19.96 |
| *Cordia spinescens* | -97.83 | 20.51 |
| *Cordia spinescens* | -97.96 | 20.28 |
| *Cordia spinescens* | -97.79 | 20.07 |
| *Cordia spinescens* | -97.93 | 20.08 |
| *Cordia spinescens* | -97.44 | 19.92 |
| *Cordia spinescens* | -97.75 | 20.00 |
| *Cordia spinescens* | -97.94 | 20.19 |
| *Cordia spinescens* | -97.34 | 19.92 |
| *Cordia spinescens* | -97.73 | 20.48 |
| *Cordia spinescens* | -97.97 | 20.24 |
| *Cordia spinescens* | -97.46 | 19.92 |
| *Cordia spinescens* | -97.65 | 19.92 |
| *Cordia spinescens* | -97.08 | 19.06 |
| *Cordia spinescens* | -94.10 | 17.79 |
| *Cordia spinescens* | -99.05 | 21.32 |
| *Cordia spinescens* | -96.94 | 19.62 |
| *Cordia spinescens* | -92.72 | 16.79 |
| *Cordia spinescens* | -92.93 | 16.65 |
| *Cordia spinescens* | -92.60 | 16.97 |
| *Cordia spinescens* | -92.48 | 17.04 |
| *Cordia spinescens* | -92.29 | 16.84 |
| *Cordia spinescens* | -96.30 | 19.20 |
| *Cordia spinescens* | -92.47 | 16.90 |
| *Cordia spinescens* | -92.43 | 16.63 |
| *Cordia spinescens* | -96.32 | 17.70 |
| *Cordia spinescens* | -93.01 | 17.20 |
| *Cordia spinescens* | -92.34 | 17.17 |
| *Cordia spinescens* | -93.09 | 16.79 |
| *Cordia spinescens* | -92.90 | 16.85 |
| *Cordia spinescens* | -92.60 | 17.76 |
| *Cordia spinescens* | -96.17 | 17.95 |
| *Cordia spinescens* | -93.49 | 18.17 |
| *Cordia spinescens* | -92.51 | 16.89 |
| *Cordia spinescens* | -93.40 | 16.87 |
| *Cordia spinescens* | -97.92 | 17.02 |
| *Cordia spinescens* | -92.89 | 16.99 |
| *Cordia spinescens* | -92.83 | 15.38 |
| *Cordia spinescens* | -92.14 | 17.11 |
| *Cordia spinescens* | -96.73 | 16.56 |
| *Cordia spinescens* | -91.83 | 17.21 |
| *Cordia spinescens* | -93.13 | 16.84 |
| *Cordia spinescens* | -98.89 | 21.63 |
| *Cordia spinescens* | -94.61 | 17.87 |
| *Cordia spinescens* | -92.29 | 16.85 |
| *Cordia spinescens* | -92.97 | 15.73 |
| *Cordia spinescens* | -96.88 | 19.52 |
| *Cordia spinescens* | -92.94 | 17.21 |
| *Cordia spinescens* | -101.00 | 18.25 |
| *Cordia spinescens* | -91.03 | 16.76 |
| *Cordia spinescens* | -91.97 | 17.45 |
| *Cordia spinescens* | -91.79 | 16.11 |
| *Cordia spinescens* | -103.16 | 18.78 |
| *Cordia spinescens* | -90.72 | 16.62 |
| *Cordia spinescens* | -95.94 | 17.26 |
| *Cordia spinescens* | -91.13 | 16.76 |
| *Cordia spinescens* | -92.76 | 16.94 |
| *Cordia spinescens* | -92.02 | 17.84 |
| *Cordia spinescens* | -97.86 | 17.04 |
| *Cordia spinescens* | -98.73 | 21.24 |
| *Cordia spinescens* | -92.94 | 17.55 |
| *Cordia spinescens* | -91.59 | 16.97 |
| *Cordia spinescens* | -92.71 | 16.41 |
| *Cordia spinescens* | -92.60 | 17.36 |
| *Cordia spinescens* | -92.12 | 16.89 |
| *Cordia spinescens* | -92.48 | 16.87 |
| *Cordia spinescens* | -92.05 | 17.70 |
| *Cordia spinescens* | -95.21 | 18.47 |
| *Cordia spinescens* | -93.64 | 17.12 |
| *Cordia spinescens* | -104.13 | 19.48 |
| *Cordia spinescens* | -104.09 | 19.64 |
| *Cordia spinescens* | -104.28 | 19.53 |
| *Cordia spinescens* | -104.27 | 19.52 |
| *Cordia spinescens* | -104.34 | 19.56 |
| *Cordia spinescens* | -104.24 | 19.66 |
| *Cordia spinescens* | -104.18 | 19.48 |
| *Cordia spinescens* | -104.23 | 19.49 |
| *Cordia spinescens* | -104.26 | 19.53 |
| *Cordia spinescens* | -104.14 | 19.43 |
| *Cordia spinescens* | -104.33 | 19.67 |
| *Cordia spinescens* | -104.21 | 19.62 |
| *Cordia spinescens* | -104.12 | 19.48 |
| *Cordia spinescens* | -104.25 | 19.44 |
| *Cordia spinescens* | -104.13 | 19.50 |
| *Cordia spinescens* | -104.35 | 19.58 |
| *Cordia spinescens* | -93.83 | 17.39 |
| *Cordia spinescens* | -97.71 | 18.17 |
| *Cordia spinescens* | -91.98 | 17.46 |
| *Cordia spinescens* | -95.21 | 18.46 |
| *Cordia spinescens* | -97.94 | 20.31 |
| *Cordia spinescens* | -92.94 | 17.21 |
| *Cordia spinescens* | -92.88 | 16.99 |
| *Cordia spinescens* | -92.29 | 16.84 |
| *Cordia spinescens* | -96.21 | 17.87 |
| *Cordia spinescens* | -92.00 | 17.85 |
| *Cordia spinescens* | -100.26 | 17.35 |
| *Cordia spinescens* | -97.15 | 18.83 |
| *Cordia spinescens* | -92.90 | 17.16 |
| *Cordia spinescens* | -94.91 | 17.95 |
| *Cordia spinescens* | -104.89 | 21.52 |
| *Cordia spinescens* | -90.71 | 16.58 |
| *Cordia spinescens* | -93.03 | 17.43 |
| *Cordia spinescens* | -98.35 | 17.09 |
| *Cordia spinescens* | -96.31 | 17.69 |
| *Cordia spinescens* | -97.07 | 18.93 |
| *Cordia spinescens* | -93.11 | 16.94 |
| *Cordia spinescens* | -104.98 | 21.36 |
| *Cordia spinescens* | -100.14 | 17.38 |
| *Cordia spinescens* | -92.65 | 18.53 |
| *Cordia spinescens* | -92.89 | 16.99 |
| *Cordia spinescens* | -96.91 | 19.14 |
| *Cordia spinescens* | -97.00 | 18.66 |
| *Cordia spinescens* | -95.21 | 18.45 |
| *Cordia spinescens* | -105.47 | 21.91 |
| *Cordia spinescens* | -95.09 | 18.23 |
| *Cordia spinescens* | -97.86 | 17.04 |
| *Cordia spinescens* | -93.16 | 17.91 |
| *Cordia spinescens* | -92.90 | 16.80 |
| *Cordia spinescens* | -92.33 | 14.76 |
| *Cordia spinescens* | -96.12 | 18.09 |
| *Cordia spinescens* | -97.01 | 16.16 |
| *Cordia spinescens* | -95.11 | 18.42 |
| *Cordia spinescens* | -103.30 | 19.38 |
| *Cordia spinescens* | -93.57 | 17.97 |
| *Cordia spinescens* | -96.35 | 17.62 |
| *Cordia spinescens* | -98.89 | 21.17 |
| *Cordia spinescens* | -91.54 | 17.81 |
| *Cordia spinescens* | -90.76 | 16.33 |
| *Cordia spinescens* | -92.58 | 17.03 |
| *Cordia spinescens* | -91.35 | 16.41 |
| *Cordia spinescens* | -105.29 | 21.54 |
| *Cordia spinescens* | -99.05 | 21.21 |
| *Cordia spinescens* | -92.09 | 17.19 |
| *Cordia spinescens* | -92.45 | 16.71 |
| *Cordia spinescens* | -92.10 | 16.97 |
| *Cordia spinescens* | -92.51 | 16.89 |
| *Cordia spinescens* | -91.93 | 16.14 |
| *Cordia spinescens* | -101.05 | 17.56 |
| *Cordia spinescens* | -95.92 | 17.36 |
| *Cordia spinescens* | -92.87 | 16.72 |
| *Cordia spinescens* | -91.42 | 17.03 |
| *Cordia spinescens* | -96.95 | 19.18 |
| *Cordia spinescens* | -93.18 | 18.41 |
| *Cordia spinescens* | -90.61 | 16.45 |
| *Cordia spinescens* | -101.27 | 17.54 |
| *Cordia spinescens* | -105.10 | 21.50 |
| *Cordia spinescens* | -96.78 | 19.49 |
| *Cordia spinescens* | -98.98 | 16.91 |
| *Cordia spinescens* | -94.32 | 17.13 |
| *Cordia spinescens* | -93.54 | 16.24 |
| *Cordia spinescens* | -96.03 | 17.26 |
| *Cordia spinescens* | -98.89 | 21.63 |
| *Cordia spinescens* | -96.17 | 17.95 |
| *Cordia spinescens* | -90.92 | 16.12 |
| *Cordia spinescens* | -96.96 | 19.46 |
| *Cordia spinescens* | -96.96 | 19.21 |
| *Cordia spinescens* | -96.68 | 19.76 |
| *Cordia spinescens* | -90.93 | 16.13 |
| *Cordia spinescens* | -97.03 | 19.09 |
| *Cordia spinescens* | -104.70 | 21.58 |
| *Cordia spinescens* | -100.29 | 17.29 |
| *Cordia spinescens* | -97.12 | 18.83 |
| *Cordia spinescens* | -93.76 | 16.80 |
| *Cordia spinescens* | -95.99 | 17.29 |
| *Cordia spinescens* | -95.07 | 18.48 |
| *Cordia spinescens* | -90.89 | 16.82 |
| *Cordia spinescens* | -100.23 | 17.47 |
| *Cordia spinescens* | -91.31 | 16.81 |
| *Cordia spinescens* | -95.10 | 18.33 |
| *Cordia spinescens* | -100.23 | 17.34 |
| *Cordia spinescens* | -96.43 | 19.66 |
| *Cordia spinescens* | -97.76 | 20.06 |
| *Cordia spinescens* | -96.76 | 18.88 |
| *Cordia spinescens* | -103.74 | 19.46 |
| *Cordia spinescens* | -98.90 | 21.16 |
| *Cordia spinescens* | -98.71 | 16.97 |
| *Cordia spinescens* | -89.43 | 18.08 |
| *Cordia spinescens* | -92.11 | 17.25 |
| *Cordia spinescens* | -97.86 | 20.52 |
| *Cordia spinescens* | -105.84 | 22.73 |
| *Cordia spinescens* | -93.53 | 17.43 |
| *Cordia spinescens* | -100.19 | 17.39 |
| *Cordia spinescens* | -93.33 | 16.88 |
| *Cordia spinescens* | -96.99 | 19.40 |
| *Cordia spinescens* | -96.86 | 19.22 |
| *Cordia spinescens* | -94.03 | 18.63 |
| *Cordia spinescens* | -96.93 | 18.89 |
| *Cordia spinescens* | -95.04 | 17.91 |
| *Cordia spinescens* | -94.03 | 17.53 |
| *Cordia spinescens* | -92.32 | 17.17 |
| *Cordia spinescens* | -91.14 | 16.76 |
| *Cordia spinescens* | -101.00 | 17.59 |
| *Cordia spinescens* | -91.13 | 16.76 |
| *Cordia spinescens* | -98.41 | 17.03 |
| *Cordia spinescens* | -95.41 | 18.31 |
| *Cordia spinescens* | -93.05 | 15.82 |
| *Cordia spinescens* | -90.68 | 16.08 |
| *Cordia spinescens* | -110.24 | 23.56 |
| *Cordia spinescens* | -114.44 | 29.91 |
| *Cordia spinescens* | -92.26 | 14.94 |
| *Cordia spinescens* | -93.05 | 15.80 |
| *Cordia spinescens* | -99.21 | 17.04 |
| *Cordia spinescens* | -97.00 | 18.90 |
| *Cordia spinescens* | -92.29 | 15.74 |
| *Cordia spinescens* | -92.71 | 16.88 |
| *Cordia spinescens* | -98.65 | 20.76 |
| *Cordia spinescens* | -93.10 | 16.97 |
| *Cordia spinescens* | -96.29 | 17.37 |
| *Cordia spinescens* | -96.03 | 17.26 |
| *Cordia spinescens* | -92.66 | 15.32 |
| *Cordia spinescens* | -92.31 | 15.08 |
| *Cordia spinescens* | -92.29 | 15.00 |
| *Cordia spinescens* | -91.26 | 17.46 |
| *Cordia spinescens* | -96.85 | 18.54 |
| *Cordia spinescens* | -91.96 | 17.44 |
| *Cordia spinescens* | -96.03 | 17.26 |
| *Cordia spinescens* | -92.34 | 17.17 |
| *Cordia spinescens* | -91.49 | 17.18 |
| *Cordia spinescens* | -93.62 | 16.27 |
| *Cordia spinescens* | -93.47 | 17.73 |
| *Cordia spinescens* | -97.51 | 20.03 |
| *Cordia spinescens* | -95.06 | 18.56 |
| *Cordia spinescens* | -96.56 | 19.35 |
| *Cordia spinescens* | -94.14 | 16.73 |
| *Cordia spinescens* | -92.04 | 17.29 |
| *Cordia spinescens* | -92.60 | 17.76 |
| *Cordia spinescens* | -96.31 | 17.76 |
| *Cordia spinescens* | -93.76 | 16.80 |
| *Cordia spinescens* | -95.26 | 18.44 |
| *Cordia spinescens* | -104.94 | 21.58 |
| *Cordia spinescens* | -95.02 | 18.44 |
| *Cordia spinescens* | -91.71 | 17.30 |
| *Cordia spinescens* | -91.07 | 16.71 |
| *Cordia spinescens* | -97.46 | 19.89 |
| *Cordia spinescens* | -94.18 | 16.73 |
| *Cordia spinescens* | -95.04 | 18.53 |
| *Cordia spinescens* | -95.07 | 18.45 |
| *Cordia spinescens* | -95.53 | 18.08 |
| *Cordia spinescens* | -92.61 | 15.55 |
| *Cordia spinescens* | -91.87 | 17.41 |
| *Cordia spinescens* | -90.48 | 16.30 |
| *Cordia spinescens* | -92.71 | 17.14 |
| *Cordia spinescens* | -91.01 | 16.76 |
| *Cordia spinescens* | -95.30 | 18.46 |
| *Cordia spinescens* | -93.05 | 15.80 |
| *Cordia spinescens* | -98.33 | 18.81 |
| *Cordia spinescens* | -96.68 | 17.96 |
| *Cordia spinescens* | -96.51 | 17.82 |
| *Cordia spinescens* | -97.23 | 19.89 |
| *Cordia spinescens* | -91.09 | 16.09 |
| *Cordia spinescens* | -100.18 | 17.43 |
| *Cordia spinescens* | -91.93 | 17.07 |
| *Cordia spinescens* | -97.90 | 17.00 |
| *Cordia spinescens* | -91.54 | 17.69 |
| *Cordia spinescens* | -92.18 | 16.98 |
| *Cordia spinescens* | -96.43 | 19.79 |
| *Cordia spinescens* | -99.47 | 17.31 |
| *Cordia spinescens* | -96.36 | 17.63 |
| *Cordia spinescens* | -97.60 | 19.96 |
| *Cordia spinescens* | -96.93 | 19.54 |
| *Cordia spinescens* | -93.38 | 18.01 |
| *Cordia spinescens* | -96.96 | 19.55 |
| *Cordia spinescens* | -99.51 | 17.25 |
| *Cordia spinescens* | -91.83 | 16.12 |
| *Cordia spinescens* | -95.08 | 18.58 |
| *Cordia spinescens* | -100.23 | 17.44 |
| *Cordia spinescens* | -92.86 | 17.03 |
| *Cordia spinescens* | -91.60 | 16.95 |
| *Cordia spinescens* | -96.01 | 17.26 |
| *Cordia spinescens* | -91.71 | 16.11 |
| *Cordia spinescens* | -93.05 | 17.65 |
| *Cordia spinescens* | -97.92 | 20.08 |
| *Cordia spinescens* | -93.47 | 16.01 |
| *Cordia spinescens* | -92.11 | 15.03 |
| *Cordia spinescens* | -104.93 | 21.34 |
| *Cordia spinescens* | -103.34 | 19.33 |
| *Cordia spinescens* | -94.89 | 18.28 |
| *Cordia spinescens* | -91.54 | 16.13 |
| *Cordia spinescens* | -97.11 | 16.20 |
| *Cordia spinescens* | -94.35 | 17.16 |
| *Cordia spinescens* | -98.50 | 17.11 |
| *Cordia spinescens* | -91.67 | 16.08 |
| *Cordia spinescens* | -92.12 | 16.89 |
| *Cordia spinescens* | -98.48 | 16.74 |
| *Cordia spinescens* | -100.31 | 17.28 |
| *Cordia spinescens* | -91.04 | 18.09 |
| *Cordia spinescens* | -92.37 | 16.89 |
| *Cordia spinescens* | -91.62 | 16.11 |
| *Cordia spinescens* | -97.86 | 17.04 |
| *Cordia spinescens* | -94.13 | 16.71 |
| *Cordia spinescens* | -92.48 | 16.87 |
| *Cordia spinescens* | -94.14 | 16.68 |
| *Cordia spinescens* | -103.06 | 18.81 |
| *Cordia spinescens* | -92.04 | 16.73 |
| *Cordia spinescens* | -92.89 | 15.44 |
| *Cordia spinescens* | -92.80 | 17.54 |
| *Cordia spinescens* | -96.68 | 18.68 |
| *Cordia spinescens* | -96.11 | 17.84 |
| *Cordia spinescens* | -97.19 | 19.95 |
| *Cordia spinescens* | -91.29 | 16.99 |
| *Cordia spinescens* | -94.11 | 16.68 |
| *Cordia spinescens* | -92.69 | 16.12 |
| *Cordia spinescens* | -92.33 | 17.17 |
| *Cordia spinescens* | -100.23 | 17.40 |
| *Cordia spinescens* | -94.08 | 16.69 |
| *Cordia spinescens* | -96.92 | 19.58 |
| *Cordia spinescens* | -100.01 | 17.20 |
| *Cordia spinescens* | -95.84 | 17.40 |
| *Cordia spinescens* | -92.66 | 15.60 |
| *Cordia spinescens* | -96.29 | 17.69 |
| *Cordia spinescens* | -92.47 | 17.01 |
| *Cordia spinescens* | -92.17 | 15.07 |
| *Cordia spinescens* | -91.67 | 16.08 |
| *Cordia spinescens* | -92.92 | 16.89 |
| *Cordia spinescens* | -97.04 | 19.32 |
| *Cordia spinescens* | -93.48 | 17.66 |
| *Cordia spinescens* | -97.22 | 20.00 |
| *Cordia spinescens* | -96.93 | 19.54 |
| *Cordia spinescens* | -100.20 | 17.36 |
| *Cordia spinescens* | -103.73 | 19.39 |
| *Cordia spinescens* | -94.44 | 18.14 |
| *Cordia spinescens* | -92.46 | 16.87 |
| *Cordia spinescens* | -91.39 | 17.32 |
| *Cordia spinescens* | -92.60 | 16.97 |
| *Cordia spinescens* | -98.48 | 16.73 |
| *Cordia spinescens* | -91.76 | 16.89 |
| *Cordia spinescens* | -101.04 | 17.49 |
| *Cordia spinescens* | -94.78 | 18.32 |
| *Cordia spinescens* | -97.09 | 18.79 |
| *Cordia spinescens* | -91.07 | 16.71 |
| *Cordia spinescens* | -91.60 | 16.11 |
| *Cordia spinescens* | -93.11 | 17.91 |
| *Cordia spinescens* | -97.20 | 19.96 |
| *Cordia spinescens* | -91.61 | 16.99 |
| *Cordia spinescens* | -103.31 | 19.47 |
| *Cordia spinescens* | -95.09 | 18.61 |
| *Cordia spinescens* | -92.62 | 16.96 |
| *Cordia spinescens* | -90.64 | 16.47 |
| *Cordia spinescens* | -92.51 | 16.82 |
| *Cordia spinescens* | -91.11 | 16.77 |
| *Cordia spinescens* | -92.40 | 17.13 |
| *Cordia spinescens* | -91.42 | 17.03 |
| *Cordia spinescens* | -88.22 | 16.96 |
| *Cordia spinescens* | -92.33 | 17.18 |
| *Cordia spinescens* | -91.01 | 16.76 |
| *Cordia spinescens* | -92.34 | 17.17 |
| *Cordia spinescens* | -93.03 | 17.42 |
| *Cordia spinescens* | -96.76 | 18.88 |
| *Cordia spinescens* | -101.28 | 18.25 |
| *Cordia spinescens* | -99.10 | 21.22 |
| *Cordia spinescens* | -116.79 | 31.98 |
| *Cordia spinescens* | -100.19 | 17.42 |
| *Cordia spinescens* | -104.12 | 19.33 |
| *Cordia spinescens* | -95.06 | 18.55 |
| *Cordia spinescens* | -96.02 | 17.93 |
| *Cordia spinescens* | -97.86 | 17.05 |
| *Cordia spinescens* | -102.88 | 20.67 |
| *Cordia spinescens* | -92.72 | 17.14 |
| *Cordia spinescens* | -93.54 | 16.09 |
| *Cordia spinescens* | -92.65 | 18.53 |
| *Cordia spinescens* | -101.00 | 17.59 |
| *Cordia spinescens* | -99.05 | 21.21 |
| *Cordia spinescens* | -105.42 | 25.05 |
| *Cordia spinescens* | -92.71 | 16.88 |
| *Cordia spinescens* | -103.38 | 19.25 |
| *Cordia spinescens* | -96.95 | 19.46 |
| *Cordia spinescens* | -91.32 | 16.82 |
| *Cordia spinescens* | -97.25 | 19.79 |
| *Cordia spinescens* | -91.07 | 16.71 |
| *Cordia spinescens* | -98.41 | 17.19 |
| *Cordia spinescens* | -94.78 | 18.31 |
| *Cordia spinescens* | -100.14 | 17.38 |
| *Cordia spinescens* | -95.07 | 18.59 |
| *Cordia spinescens* | -100.43 | 17.21 |
| *Cordia spinescens* | -92.41 | 15.12 |
| *Cordia spinescens* | -94.34 | 17.11 |
| *Cordia spinescens* | -97.03 | 19.09 |
| *Cordia spinescens* | -98.48 | 16.75 |
| *Cordia spinescens* | -92.37 | 16.89 |
| *Cordia spinescens* | -96.96 | 19.15 |
| *Cordia spinescens* | -97.13 | 18.21 |
| *Cordia spinescens* | -97.14 | 18.19 |
| *Cordia spinescens* | -97.44 | 20.40 |
| *Cordia spinescens* | -96.90 | 18.84 |
| *Cordia spinescens* | -95.12 | 18.37 |
| *Cordia spinescens* | -96.82 | 19.21 |
| *Cordia spinescens* | -95.02 | 18.44 |
| *Cordia spinescens* | -95.98 | 17.28 |
| *Cordia spinescens* | -97.22 | 19.87 |
| *Cordia spinescens* | -95.06 | 18.56 |
| *Cordia spinescens* | -94.76 | 18.31 |
| *Cordia spinescens* | -91.99 | 16.71 |
| *Cordia spinescens* | -96.42 | 19.76 |
| *Cordia spinescens* | -91.11 | 16.75 |
| *Cordia spinescens* | -92.38 | 16.89 |
| *Cordia spinescens* | -97.24 | 19.93 |
| *Cordia spinescens* | -96.95 | 19.18 |
| *Cordia spinescens* | -96.86 | 19.22 |
| *Cordia spinescens* | -96.98 | 19.52 |
| *Cordia spinescens* | -91.62 | 16.11 |
| *Cordia spinescens* | -99.10 | 21.28 |
| *Cordia spinescens* | -97.09 | 18.86 |
| *Cordia spinescens* | -93.28 | 16.71 |
| *Cordia spinescens* | -98.34 | 20.76 |
| *Cordia spinescens* | -96.79 | 19.36 |
| *Cordia spinescens* | -92.32 | 17.17 |
| *Cordia spinescens* | -97.29 | 19.70 |
| *Cordia spinescens* | -94.75 | 18.35 |
| *Cordia spinescens* | -97.11 | 18.85 |
| *Cordia spinescens* | -95.11 | 18.42 |
| *Cordia spinescens* | -90.94 | 16.18 |
| *Cordia spinescens* | -97.44 | 19.95 |
| *Cordia spinescens* | -103.31 | 19.48 |
| *Cordia spinescens* | -94.88 | 18.27 |
| *Cordia spinescens* | -94.76 | 18.33 |
| *Cordia spinescens* | -97.10 | 18.85 |
| *Cordia spinescens* | -98.63 | 20.73 |
| *Cordia spinescens* | -92.73 | 17.14 |
| *Cordia spinescens* | -93.39 | 17.84 |
| *Cordia spinescens* | -92.47 | 17.30 |
| *Cordia spinescens* | -98.24 | 21.36 |
| *Cordia spinescens* | -96.94 | 19.51 |
| *Cordia spinescens* | -92.11 | 15.03 |
| *Cordia spinescens* | -96.43 | 19.35 |
| *Cordia spinescens* | -96.94 | 19.62 |
| *Cordia spinescens* | -97.09 | 18.79 |
| *Cordia spinescens* | -96.96 | 19.43 |
| *Cordia spinescens* | -97.18 | 19.99 |
| *Cordia spinescens* | -95.61 | 18.05 |
| *Cordia spinescens* | -97.80 | 20.07 |
| *Cordia spinescens* | -93.38 | 17.99 |
| *Cordia spinescens* | -92.26 | 14.91 |
| *Cordia spinescens* | -96.75 | 18.87 |
| *Cordia spinescens* | -97.05 | 19.18 |
| *Cordia spinescens* | -97.19 | 19.95 |
| *Cordia spinescens* | -95.04 | 18.50 |
| *Cordia spinescens* | -96.86 | 19.20 |
| *Cordia spinescens* | -96.94 | 19.51 |
| *Cordia spinescens* | -96.86 | 19.20 |
| *Cordia spinescens* | -96.17 | 19.22 |
| *Cordia spinescens* | -92.25 | 14.93 |
| *Cordia spinescens* | -97.03 | 19.11 |
| *Cordia spinescens* | -96.69 | 19.79 |
| *Cordia spinescens* | -97.07 | 18.93 |
| *Cordia spinescens* | -92.59 | 15.70 |
| *Cordia spinescens* | -97.24 | 19.90 |
| *Cordia spinescens* | -91.07 | 16.71 |
| *Cordia spinescens* | -92.14 | 16.93 |
| *Cordia spinescens* | -96.93 | 19.54 |
| *Cordia spinescens* | -94.03 | 18.20 |
| *Cordia spinescens* | -96.34 | 17.73 |
| *Cordia spinescens* | -97.08 | 18.91 |
| *Cordia spinescens* | -96.91 | 20.36 |
| *Cordia spinescens* | -96.30 | 19.20 |
| *Cordia spinescens* | -95.06 | 18.56 |
| *Cordia spinescens* | -92.26 | 16.83 |
| *Cordia spinescens* | -96.96 | 19.21 |
| *Cordia spinescens* | -105.73 | 23.03 |
| *Cordia spinescens* | -96.39 | 19.61 |
| *Cordia spinescens* | -95.10 | 18.33 |
| *Cordia spinescens* | -92.17 | 15.07 |
| *Cordia spinescens* | -94.75 | 18.32 |
| *Cordia spinescens* | -91.99 | 17.44 |
| *Cordia spinescens* | -96.90 | 19.57 |
| *Cordia spinescens* | -90.66 | 16.53 |
| *Cordia spinescens* | -91.13 | 17.77 |
| *Cordia spinescens* | -95.08 | 17.91 |
| *Cordia spinescens* | -94.91 | 17.95 |
| *Cordia spinescens* | -96.83 | 19.59 |
| *Cordia spinescens* | -95.03 | 18.51 |
| *Cordia spinescens* | -100.24 | 17.33 |
| *Cordia spinescens* | -97.00 | 16.15 |
| *Cordia spinescens* | -104.01 | 19.41 |
| *Cordia spinescens* | -92.35 | 17.20 |
| *Cordia spinescens* | -93.62 | 17.81 |
| *Cordia spinescens* | -93.08 | 15.86 |
| *Cordia spinescens* | -93.07 | 16.82 |
| *Cordia spinescens* | -92.66 | 15.37 |
| *Cordia spinescens* | -93.59 | 16.22 |
| *Cordia spinescens* | -92.72 | 15.68 |
| *Cordia spinescens* | -96.94 | 19.51 |
| *Cordia spinescens* | -92.76 | 16.94 |
| *Cordia spinescens* | -93.39 | 17.83 |
| *Cordia spinescens* | -91.58 | 16.98 |
| *Cordia spinescens* | -92.60 | 17.00 |
| *Cordia spinescens* | -92.57 | 16.96 |
| *Cordia spinescens* | -92.27 | 17.66 |
| *Cordia spinescens* | -92.32 | 17.17 |
| *Cordia spinescens* | -92.15 | 15.16 |
| *Cordia spinescens* | -92.98 | 15.68 |
| *Cordia spinescens* | -91.83 | 17.21 |
| *Cordia spinescens* | -91.13 | 16.76 |
| *Cordia spinescens* | -92.60 | 16.97 |
| *Cordia spinescens* | -91.99 | 16.70 |
| *Cordia spinescens* | -90.92 | 16.10 |
| *Cordia spinescens* | -92.14 | 16.93 |
| *Cordia spinescens* | -91.29 | 16.99 |
| *Cordia spinescens* | -94.88 | 18.27 |
| *Cordia spinescens* | -95.06 | 18.48 |
| *Cordia spinescens* | -97.04 | 19.21 |
| *Cordia spinescens* | -95.02 | 18.37 |
| *Cordia spinescens* | -94.76 | 18.26 |
| *Cordia spinescens* | -102.12 | 19.15 |
| *Cordia spinescens* | -76.83 | 2.14 |
| *Cordia spinescens* | -73.49 | 7.13 |
| *Cordia spinescens* | -74.46 | 4.77 |
| *Cordia spinescens* | -75.63 | 4.60 |
| *Cordia spinescens* | -76.15 | 4.78 |
| *Cordia spinescens* | -76.31 | 6.55 |
| *Cordia spinescens* | -75.17 | 6.07 |
| *Cordia spinescens* | -76.27 | 7.02 |
| *Cordia spinescens* | -78.13 | 1.67 |
| *Cordia spinescens* | -75.55 | 3.77 |
| *Cordia spinescens* | -74.44 | 4.20 |
| *Cordia spinescens* | -75.69 | 9.40 |
| *Cordia spinescens* | -75.00 | 6.17 |
| *Cordia spinescens* | -76.89 | 5.16 |
| *Cordia spinescens* | -74.34 | 5.25 |
| *Cordia spinescens* | -76.15 | 4.78 |
| *Cordia spinescens* | -77.14 | 7.97 |
| *Cordia spinescens* | -77.15 | 7.98 |
| *Cordia spinescens* | -75.07 | 7.31 |
| *Cordia spinescens* | -74.71 | 7.08 |
| *Cordia spinescens* | -76.07 | 5.87 |
| *Cordia spinescens* | -76.73 | 4.82 |
| *Cordia spinescens* | -76.72 | 6.21 |
| *Cordia spinescens* | -75.07 | 4.92 |
| *Cordia spinescens* | -72.29 | 7.09 |
| *Cordia spinescens* | -72.37 | 7.09 |
| *Cordia spinescens* | -72.55 | 7.27 |
| *Cordia spinescens* | -72.26 | 7.04 |
| *Cordia spinescens* | -72.58 | 7.26 |
| *Cordia spinescens* | -72.60 | 7.69 |
| *Cordia spinescens* | -72.73 | 6.68 |
| *Cordia spinescens* | -74.34 | 4.60 |
| *Cordia spinescens* | -74.43 | 4.93 |
| *Cordia spinescens* | -75.03 | 6.09 |
| *Cordia spinescens* | -75.02 | 6.09 |
| *Cordia spinescens* | -75.13 | 6.28 |
| *Cordia spinescens* | -75.05 | 7.22 |
| *Cordia spinescens* | -76.92 | 4.70 |
| *Cordia spinescens* | -76.53 | 5.75 |
| *Cordia spinescens* | -76.23 | 5.82 |
| *Cordia spinescens* | -76.23 | 5.81 |
| *Cordia spinescens* | -76.66 | 5.69 |
| *Cordia spinescens* | -76.36 | 5.72 |
| *Cordia spinescens* | -69.98 | -4.10 |
| *Cordia spinescens* | -73.93 | 6.64 |
| *Cordia spinescens* | -75.06 | 7.32 |
| *Cordia spinescens* | -74.17 | 10.65 |
| *Cordia spinescens* | -75.23 | 6.80 |
| *Cordia spinescens* | -77.40 | 6.23 |
| *Cordia spinescens* | -74.40 | 4.38 |
| *Cordia spinescens* | -70.47 | -3.62 |
| *Cordia spinescens* | -75.75 | 6.23 |
| *Cordia spinescens* | -74.77 | 7.02 |
| *Cordia spinescens* | -75.90 | 6.64 |
| *Cordia spinescens* | -74.42 | 4.92 |
| *Cordia spinescens* | -77.36 | 8.65 |
| *Cordia spinescens* | -75.95 | 2.21 |
| *Cordia spinescens* | -73.83 | 4.02 |
| *Cordia spinescens* | -75.82 | 4.22 |
| *Cordia spinescens* | -75.68 | 4.68 |
| *Cordia spinescens* | -76.08 | 5.98 |
| *Cordia spinescens* | -74.29 | 5.55 |
| *Cordia spinescens* | -75.57 | 4.74 |
| *Cordia spinescens* | -75.61 | 4.65 |
| *Cordia spinescens* | -74.87 | 5.96 |
| *Cordia spinescens* | -74.87 | 5.96 |
| *Cordia spinescens* | -74.78 | 6.00 |
| *Cordia spinescens* | -75.28 | 7.47 |
| *Cordia spinescens* | -75.73 | 4.86 |
| *Cordia spinescens* | -76.15 | 4.78 |
| *Cordia spinescens* | -75.70 | 1.63 |
| *Cordia spinescens* | -75.75 | 6.23 |
| *Cordia spinescens* | -74.40 | 4.38 |
| *Cordia spinescens* | -76.15 | 4.78 |
| *Cordia spinescens* | -75.03 | 6.11 |
| *Cordia spinescens* | -75.61 | 4.65 |
| *Cordia spinescens* | -74.29 | 5.55 |
| *Cordia spinescens* | -76.54 | 5.74 |
| *Cordia spinescens* | -74.34 | 5.26 |
| *Cordia spinescens* | -74.46 | 4.36 |
| *Cordia spinescens* | -74.78 | 6.00 |
| *Cordia spinescens* | -76.72 | 6.21 |
| *Cordia spinescens* | -75.57 | 4.74 |
| *Cordia spinescens* | -75.07 | 4.92 |
| *Cordia spinescens* | -76.23 | 5.81 |
| *Cordia spinescens* | -75.68 | 4.68 |
| *Cordia spinescens* | -76.61 | 6.27 |
| *Cordia spinescens* | -76.27 | 7.02 |
| *Cordia spinescens* | -76.27 | 5.75 |
| *Cordia spinescens* | -75.55 | 3.77 |
| *Cordia spinescens* | -76.15 | 4.78 |
| *Cordia spinescens* | -75.06 | 7.32 |
| *Cordia spinescens* | -74.46 | 4.77 |
| *Cordia spinescens* | -74.43 | 4.71 |
| *Cordia spinescens* | -75.07 | 7.31 |
| *Cordia spinescens* | -75.05 | 7.22 |
| *Cordia spinescens* | -76.31 | 6.55 |
| *Cordia spinescens* | -74.78 | 6.00 |
| *Cordia spinescens* | -75.13 | 6.28 |
| *Cordia spinescens* | -75.02 | 6.09 |
| *Cordia spinescens* | -74.77 | 7.02 |
| *Cordia spinescens* | -76.15 | 4.78 |
| *Cordia spinescens* | -74.42 | 4.92 |
| *Cordia spinescens* | -75.23 | 6.80 |
| *Cordia spinescens* | -76.08 | 5.98 |
| *Cordia spinescens* | -74.17 | 11.30 |
| *Cordia spinescens* | -78.63 | 0.13 |
| *Cordia spinescens* | -79.67 | 0.08 |
| *Cordia spinescens* | -79.67 | 0.08 |
| *Cordia spinescens* | -87.08 | 14.03 |
| *Cordia spinescens* | -82.40 | 8.80 |
| *Cordia spinescens* | -87.46 | 15.71 |
| *Cordia spinescens* | -104.92 | 21.33 |
| *Cordia spinescens* | -95.07 | 18.48 |
| *Cordia spinescens* | -100.28 | 17.40 |
| *Cordia spinescens* | -82.86 | 9.02 |
| *Cordia spinescens* | -84.12 | 9.84 |
| *Cordia spinescens* | -84.37 | 9.72 |
| *Cordia spinescens* | -84.52 | 9.75 |
| *Cordia spinescens* | -83.75 | 9.21 |
| *Cordia spinescens* | -78.47 | 0.85 |
| *Cordia spinescens* | -78.90 | 0.80 |
| *Cordia spinescens* | -78.87 | 0.80 |
| *Cordia spinescens* | -78.88 | 0.78 |
| *Cordia spinescens* | -76.80 | -2.52 |
| *Cordia spinescens* | -77.58 | 0.05 |
| *Cordia spinescens* | -89.08 | 14.35 |
| *Cordia spinescens* | -93.07 | 16.82 |
| *Cordia spinescens* | -89.44 | 16.20 |
| *Cordia spinescens* | -90.66 | 15.92 |
| *Cordia spinescens* | -93.05 | 15.80 |
| *Cordia spinescens* | -79.03 | 0.08 |
| *Cordia spinescens* | -78.95 | -1.98 |
| *Cordia spinescens* | -76.99 | -6.03 |
| *Cordia spinescens* | -87.06 | 14.04 |
| *Cordia spinescens* | -79.96 | 9.36 |
| *Cordia spinescens* | -89.79 | 16.92 |
| *Cordia spinescens* | -79.92 | 8.69 |
| *Cordia spinescens* | -79.85 | 9.17 |
| *Cordia spinescens* | -90.87 | 16.20 |
| *Cordia spinescens* | -104.92 | 21.30 |
| *Cordia spinescens* | -105.70 | 23.03 |
| *Cordia spinescens* | -78.18 | -2.10 |
| *Cordia spinescens* | -82.60 | 8.85 |
| *Cordia spinescens* | -67.95 | -14.40 |
| *Cordia spinescens* | -77.10 | -6.08 |
| *Cordia spinescens* | -79.31 | 9.24 |
| *Cordia spinescens* | -79.06 | 8.62 |
| *Cordia spinescens* | -77.78 | 8.97 |
| *Cordia spinescens* | -79.50 | 0.38 |
| *Cordia spinescens* | -79.73 | 0.35 |
| *Cordia spinescens* | -64.82 | -17.00 |
| *Cordia spinescens* | -84.07 | 10.00 |
| *Cordia spinescens* | -83.05 | 9.85 |
| *Cordia spinescens* | -82.81 | 9.74 |
| *Cordia spinescens* | -83.50 | 8.67 |
| *Cordia spinescens* | -64.80 | -16.90 |
| *Cordia spinescens* | -104.01 | 19.42 |
| *Cordia spinescens* | -91.22 | 15.84 |
| *Cordia spinescens* | -91.01 | 15.72 |
| *Cordia spinescens* | -90.99 | 15.69 |
| *Cordia spinescens* | -91.52 | 16.06 |
| *Cordia spinescens* | -91.21 | 15.84 |
| *Cordia spinescens* | -91.31 | 15.94 |
| *Cordia spinescens* | -73.41 | -3.96 |
| *Cordia spinescens* | -97.80 | 16.68 |
| *Cordia spinescens* | -88.98 | 15.68 |
| *Cordia spinescens* | -80.62 | 8.84 |
| *Cordia spinescens* | -97.31 | 20.02 |
| *Cordia spinescens* | -97.31 | 20.02 |
| *Cordia spinescens* | -83.00 | 9.00 |
| *Cordia spinescens* | -72.87 | 3.88 |
| *Cordia spinescens* | -73.00 | 8.00 |
| *Cordia spinescens* | -75.50 | 7.00 |
| *Cordia spinescens* | -74.17 | 5.00 |
| *Cordia spinescens* | -73.25 | 7.00 |
| *Cordia spinescens* | -74.50 | 10.00 |
| *Cordia spinescens* | -77.00 | 6.00 |
| *Cordia spinescens* | -65.91 | 7.08 |
| *Cordia spinescens* | -64.44 | -16.73 |
| *Cordia spinescens* | -74.14 | -9.18 |
| *Cordia spinescens* | -96.67 | 19.33 |
| *Cordia spinescens* | -75.25 | 3.75 |
| *Cordia spinescens* | -97.03 | 19.20 |
| *Cordia spinescens* | -95.02 | 18.44 |
| *Cordia spinescens* | -78.19 | -1.46 |
| *Cordia spinescens* | -75.50 | 5.25 |
| *Cordia spinescens* | -52.87 | -10.83 |
| *Cordia spinescens* | -96.93 | 19.51 |
| *Cordia spinescens* | -95.22 | 21.12 |
| *Cordia spinescens* | -76.50 | 3.75 |
| *Cordia spinescens* | -81.25 | 8.17 |
| *Cordia spinescens* | -75.67 | -9.05 |
| *Cordia spinescens* | -96.68 | 19.78 |
| *Cordia spinescens* | -97.07 | 18.93 |
| *Cordia spinescens* | -74.26 | -8.15 |
| *Cordia spinescens* | -96.43 | 19.35 |
| *Cordia spinescens* | -72.50 | 5.50 |
| *Cordia spinescens* | -96.77 | 19.48 |
| *Cordia spinescens* | -88.92 | 16.83 |
| *Cordia spinescens* | -70.24 | -2.37 |
| *Cordia spinescens* | -102.53 | 23.95 |
| *Cordia spinescens* | -58.97 | 4.79 |
| *Cordia spinescens* | -78.28 | -1.10 |
| *Cordia spinescens* | -85.18 | 12.22 |
| *Cordia spinescens* | -89.10 | 14.18 |
| *Cordia spinescens* | -56.83 | 4.78 |
| *Cordia spinescens* | -84.58 | 9.75 |
| *Cordia spinescens* | -85.28 | 12.20 |
| *Cordia spinescens* | -71.68 | 8.92 |
| *Cordia spinescens* | -66.07 | -11.00 |
| *Cordia spinescens* | -82.60 | 8.88 |
| *Cordia spinescens* | -90.25 | 15.25 |
| *Cordia spinescens* | -72.22 | 7.60 |
| *Cordia spinescens* | -79.37 | -0.35 |
| *Cordia spinescens* | -89.08 | 14.33 |
| *Cordia spinescens* | -80.47 | 8.78 |
| *Cordia spinescens* | -83.22 | 8.68 |
| *Cordia spinescens* | -102.00 | 23.00 |
| *Cordia spinescens* | -77.68 | -2.33 |
| *Cordia spinescens* | -89.03 | 17.08 |
| *Cordia spinescens* | -88.95 | 16.27 |
| *Cordia spinescens* | -89.07 | 18.00 |
| *Cordia spinescens* | -88.93 | 16.17 |
| *Cordia spinescens* | 0.00 | 0.00 |
| *Cordia spinescens* | -80.12 | 8.63 |
| *Cordia spinescens* | -73.45 | 7.82 |
| *Cordia spinescens* | -73.27 | 7.05 |
| *Cordia spinescens* | -73.01 | 6.96 |
| *Cordia spinescens* | -74.02 | 6.69 |
| *Cordia spinescens* | -73.36 | 6.44 |
| *Cordia spinescens* | -76.37 | 4.08 |
| *Cordia spinescens* | -76.59 | 3.24 |
| *Cordia spinescens* | -76.06 | 4.39 |
| *Cordia spinescens* | -76.67 | 3.74 |
| *Cordia spinescens* | -76.66 | 3.76 |
| *Cordia spinescens* | -76.33 | 3.99 |
| *Cordia spinescens* | -88.68 | 17.22 |
| *Cordia spinescens* | -88.88 | 17.02 |
| *Cordia spinescens* | -90.36 | 15.70 |
| *Cordia spinescens* | -75.66 | 4.65 |
| *Cordia spinescens* | -76.70 | 5.72 |
| *Cordia spinescens* | -75.44 | 5.81 |
| *Cordia spinescens* | -75.80 | 7.13 |
| *Cordia spinescens* | -75.05 | 7.22 |
| *Cordia spinescens* | -74.03 | 2.04 |
| *Cordia spinescens* | -76.37 | 6.75 |
| *Cordia spinescens* | -75.00 | 6.08 |
| *Cordia spinescens* | -78.59 | 1.40 |
| *Cordia spinescens* | -73.91 | 7.01 |
| *Cordia spinescens* | -76.67 | 5.70 |
| *Cordia spinescens* | -75.69 | 9.39 |
| *Cordia spinescens* | -74.70 | 7.08 |
| *Cordia spinescens* | -75.75 | 5.72 |
| *Cordia spinescens* | -75.13 | 6.03 |
| *Cordia spinescens* | -75.00 | 6.04 |
| *Cordia spinescens* | -75.35 | 7.48 |
| *Cordia spinescens* | -75.97 | 5.97 |
| *Cordia spinescens* | -75.00 | 6.58 |
| *Cordia spinescens* | -76.01 | 5.87 |
| *Cordia spinescens* | -76.08 | 5.83 |
| *Cordia spinescens* | -75.05 | 7.22 |
| *Cordia spinescens* | -75.90 | 5.65 |
| *Cordia spinescens* | -75.85 | 5.64 |
| *Cordia spinescens* | -75.59 | 6.30 |
| *Cordia spinescens* | -75.14 | 6.01 |
| *Cordia spinescens* | -75.36 | 6.40 |
| *Cordia spinescens* | -75.60 | 6.29 |
| *Cordia spinescens* | -75.60 | 6.13 |
| *Cordia spinescens* | -75.51 | 6.32 |
| *Cordia spinescens* | -75.65 | 5.91 |
| *Cordia spinescens* | -75.59 | 4.73 |
| *Cordia spinescens* | -77.33 | 8.62 |
| *Cordia spinescens* | -77.37 | 8.66 |
| *Cordia spinescens* | -75.15 | 6.86 |
| *Cordia spinescens* | -75.10 | 6.77 |
| *Cordia spinescens* | -75.59 | 4.99 |
| *Cordia spinescens* | -75.20 | 6.58 |
| *Cordia spinescens* | -75.33 | 6.44 |
| *Cordia spinescens* | -75.99 | 5.64 |
| *Cordia spinescens* | -75.73 | 4.90 |
| *Cordia spinescens* | -75.22 | 7.04 |
| *Cordia spinescens* | -75.58 | 6.27 |
| *Cordia spinescens* | -76.63 | 1.20 |
| *Cordia spinescens* | -75.10 | 6.79 |
| *Cordia spinescens* | -103.68 | 19.44 |
| *Cordia spinescens* | -75.95 | 2.21 |
| *Cordia spinescens* | -75.94 | 2.21 |
| *Cordia spinescens* | -75.62 | 4.68 |
| *Cordia spinescens* | -69.75 | -2.91 |
| *Cordia spinescens* | -76.63 | 1.20 |
| *Cordia spinescens* | -96.95 | 17.82 |
| *Cordia spinescens* | -91.93 | 17.74 |
| *Cordia spinescens* | -91.87 | 17.76 |
| *Cordia spinescens* | -90.85 | 17.89 |
| *Cordia spinescens* | -104.91 | 21.42 |
| *Cordia spinescens* | -91.10 | 16.73 |
| *Cordia spinescens* | -98.89 | 21.17 |
| *Cordia spinescens* | -91.25 | 17.03 |
| *Cordia spinescens* | -91.29 | 16.98 |
| *Cordia spinescens* | -91.32 | 17.01 |
| *Cordia spinescens* | -91.30 | 17.10 |
| *Cordia spinescens* | -91.25 | 16.94 |
| *Cordia spinescens* | -91.60 | 16.80 |
| *Cordia spinescens* | -91.25 | 16.93 |
| *Cordia spinescens* | -93.47 | 16.01 |
| *Cordia spinescens* | -99.05 | 21.21 |
| *Cordia spinescens* | -91.32 | 16.82 |
| *Cordia spinescens* | -100.43 | 17.21 |
| *Cordia spinescens* | -92.65 | 18.53 |
| *Cordia spinescens* | -97.02 | 19.08 |
| *Cordia spinescens* | -96.96 | 19.15 |
| *Cordia spinescens* | -100.19 | 17.42 |
| *Cordia spinescens* | -96.76 | 18.88 |
| *Cordia spinescens* | -97.25 | 19.79 |
| *Cordia spinescens* | -96.86 | 19.22 |
| *Cordia spinescens* | -92.71 | 16.88 |
| *Cordia spinescens* | -98.41 | 17.19 |
| *Cordia spinescens* | -100.14 | 17.38 |
| *Cordia spinescens* | -92.48 | 16.87 |
| *Cordia spinescens* | -92.41 | 15.12 |
| *Cordia spinescens* | -91.07 | 16.71 |
| *Cordia spinescens* | -92.37 | 16.89 |
| *Cordia spinescens* | -96.95 | 19.46 |
| *Cordia spinescens* | -92.72 | 17.14 |
| *Cordia spinescens* | -102.88 | 20.67 |
| *Cordia spinescens* | -93.54 | 16.09 |
| *Cordia spinescens* | -104.12 | 19.33 |
| *Cordia spinescens* | -101.28 | 18.25 |
| *Cordia spinescens* | -96.77 | 19.48 |
| *Cordia spinescens* | -94.34 | 17.11 |
| *Cordia spinescens* | -103.38 | 19.25 |
| *Cordia spinescens* | -96.03 | 17.26 |
| *Cordia spinescens* | -99.10 | 21.22 |
| *Cordia spinescens* | -91.13 | 17.77 |
| *Cordia spinescens* | -97.10 | 18.85 |
| *Cordia spinescens* | -97.22 | 19.92 |
| *Cordia spinescens* | -97.19 | 19.95 |
| *Cordia spinescens* | -96.90 | 18.84 |
| *Cordia spinescens* | -91.99 | 16.71 |
| *Cordia spinescens* | -97.80 | 20.05 |
| *Cordia spinescens* | -96.33 | 17.72 |
| *Cordia spinescens* | -92.14 | 16.93 |
| *Cordia spinescens* | -92.47 | 17.30 |
| *Cordia spinescens* | -92.25 | 14.93 |
| *Cordia spinescens* | -92.46 | 16.87 |
| *Cordia spinescens* | -96.39 | 19.61 |
| *Cordia spinescens* | -95.06 | 18.54 |
| *Cordia spinescens* | -96.96 | 19.43 |
| *Cordia spinescens* | -95.09 | 18.59 |
| *Cordia spinescens* | -97.22 | 19.90 |
| *Cordia spinescens* | -98.62 | 20.72 |
| *Cordia spinescens* | -92.25 | 14.90 |
| *Cordia spinescens* | -92.27 | 14.93 |
| *Cordia spinescens* | -93.27 | 16.70 |
| *Cordia spinescens* | -95.98 | 17.28 |
| *Cordia spinescens* | -103.30 | 19.47 |
| *Cordia spinescens* | -99.08 | 21.22 |
| *Cordia spinescens* | -93.38 | 17.83 |
| *Cordia spinescens* | -93.37 | 17.98 |
| *Cordia spinescens* | -92.17 | 15.07 |
| *Cordia spinescens* | -105.83 | 22.98 |
| *Cordia spinescens* | -91.98 | 17.50 |
| *Cordia spinescens* | -90.65 | 16.52 |
| *Cordia spinescens* | -92.72 | 17.13 |
| *Cordia spinescens* | -92.32 | 17.17 |
| *Cordia spinescens* | -91.32 | 17.08 |
| *Cordia spinescens* | -91.63 | 16.11 |
| *Cordia spinescens* | -98.12 | 18.85 |
| *Cordia spinescens* | -97.08 | 18.85 |
| *Cordia spinescens* | -97.44 | 20.40 |
| *Cordia spinescens* | -97.03 | 19.20 |
| *Cordia spinescens* | -97.02 | 19.10 |
| *Cordia spinescens* | -96.67 | 18.67 |
| *Cordia spinescens* | -96.75 | 18.87 |
| *Cordia spinescens* | -96.43 | 19.35 |
| *Cordia spinescens* | -94.89 | 18.27 |
| *Cordia spinescens* | -97.08 | 18.92 |
| *Cordia spinescens* | -95.02 | 18.44 |
| *Cordia spinescens* | -95.22 | 21.12 |
| *Cordia spinescens* | -96.90 | 19.57 |
| *Cordia spinescens* | -97.11 | 20.04 |
| *Cordia spinescens* | -98.23 | 21.35 |
| *Cordia spinescens* | -95.11 | 18.42 |
| *Cordia spinescens* | -96.94 | 19.51 |
| *Cordia spinescens* | -95.03 | 18.53 |
| *Cordia spinescens* | -96.90 | 19.13 |
| *Cordia spinescens* | -96.42 | 19.78 |
| *Cordia spinescens* | -96.82 | 19.58 |
| *Cordia spinescens* | -96.93 | 19.54 |
| *Cordia spinescens* | -95.08 | 18.45 |
| *Cordia spinescens* | -96.78 | 19.35 |
| *Cordia spinescens* | -95.28 | 18.58 |
| *Cordia spinescens* | -95.15 | 18.38 |
| *Cordia spinescens* | -95.07 | 17.90 |
| *Cordia spinescens* | -95.07 | 18.53 |
| *Cordia spinescens* | -96.82 | 19.21 |
| *Cordia spinescens* | -97.17 | 19.98 |
| *Cordia spinescens* | -96.94 | 19.63 |
| *Cordia spinescens* | -96.98 | 19.53 |
| *Cordia spinescens* | -96.95 | 19.18 |
| *Cordia spinescens* | -96.86 | 19.22 |
| *Cordia spinescens* | -96.96 | 19.21 |
| *Cordia spinescens* | -96.95 | 18.89 |
| *Cordia spinescens* | -97.00 | 19.40 |
| *Cordia spinescens* | -96.75 | 19.32 |
| *Cordia spinescens* | -97.29 | 19.70 |
| *Cordia spinescens* | -96.30 | 19.18 |
| *Cordia spinescens* | -96.68 | 19.78 |
| *Cordia spinescens* | -95.61 | 18.05 |
| *Cordia spinescens* | -94.75 | 18.32 |
| *Cordia spinescens* | -94.02 | 18.62 |
| *Cordia spinescens* | -97.45 | 19.88 |
| *Cordia spinescens* | -92.37 | 16.88 |
| *Cordia spinescens* | -96.97 | 19.52 |
| *Cordia spinescens* | -94.76 | 18.31 |
| *Cordia spinescens* | -94.75 | 18.35 |
| *Cordia spinescens* | -94.78 | 18.32 |
| *Cordia spinescens* | -94.75 | 18.32 |
| *Cordia spinescens* | -96.14 | 19.19 |
| *Cordia spinescens* | -92.26 | 16.83 |
| *Cordia spinescens* | -91.05 | 16.72 |
| *Cordia spinescens* | -84.06 | 10.07 |
| *Cordia spinescens* | -82.56 | 9.56 |
| *Cordia spinescens* | -79.37 | -0.35 |
| *Cordia spinescens* | -78.88 | 0.78 |
| *Cordia spinescens* | -78.23 | 0.73 |
| *Cordia spinescens* | -78.38 | 1.03 |
| *Cordia spinescens* | -78.47 | 0.85 |
| *Cordia spinescens* | -78.28 | -1.10 |
| *Cordia spinescens* | -78.87 | 0.80 |
| *Cordia spinescens* | -83.23 | 8.69 |
| *Cordia spinescens* | -91.31 | 17.01 |
| *Cordia spinescens* | -83.21 | 8.68 |
| *Cordia spinescens* | -82.63 | 9.56 |
| *Cordia spinescens* | -84.70 | 11.04 |
| *Cordia spinescens* | -85.05 | 12.88 |
| *Cordia spinescens* | -83.44 | 8.52 |
| *Cordia spinescens* | -84.18 | 9.73 |
| *Cordia spinescens* | -84.68 | 10.15 |
| *Cordia spinescens* | -83.22 | 8.69 |
| *Cordia spinescens* | -83.99 | 9.68 |
| *Cordia spinescens* | -83.97 | 9.74 |
| *Cordia spinescens* | -84.07 | 10.08 |
| *Cordia spinescens* | -83.34 | 9.78 |
| *Cordia spinescens* | -83.49 | 8.56 |
| *Cordia spinescens* | -83.50 | 10.17 |
| *Cordia spinescens* | -84.01 | 9.54 |
| *Cordia spinescens* | -83.19 | 9.89 |
| *Cordia spinescens* | -84.79 | 10.27 |
| *Cordia spinescens* | -83.73 | 10.64 |
| *Cordia spinescens* | -82.96 | 9.02 |
| *Cordia spinescens* | -84.78 | 10.36 |
| *Cordia spinescens* | -83.44 | 8.72 |
| *Cordia spinescens* | -82.91 | 8.96 |
| *Cordia spinescens* | -83.78 | 9.70 |
| *Cordia spinescens* | -82.97 | 9.47 |
| *Cordia spinescens* | -84.26 | 9.90 |
| *Cordia spinescens* | -84.99 | 10.61 |
| *Cordia spinescens* | -82.83 | 8.95 |
| *Cordia spinescens* | -82.95 | 8.78 |
| *Cordia spinescens* | -84.76 | 10.91 |
| *Cordia spinescens* | -83.59 | 9.39 |
| *Cordia spinescens* | -83.34 | 8.99 |
| *Cordia spinescens* | -83.56 | 8.70 |
| *Cordia spinescens* | -84.82 | 10.27 |
| *Cordia spinescens* | -83.25 | 8.76 |
| *Cordia spinescens* | -83.51 | 10.54 |
| *Cordia spinescens* | -83.85 | 9.51 |
| *Cordia spinescens* | -83.78 | 9.75 |
| *Cordia spinescens* | -83.61 | 8.73 |
| *Cordia spinescens* | -83.41 | 8.79 |
| *Cordia spinescens* | -83.38 | 8.75 |
| *Cordia spinescens* | -83.81 | 9.73 |
| *Cordia spinescens* | -82.85 | 8.95 |
| *Cordia spinescens* | -83.43 | 10.10 |
| *Cordia spinescens* | -84.16 | 10.25 |
| *Cordia spinescens* | -83.51 | 10.58 |
| *Cordia spinescens* | -83.58 | 10.78 |
| *Cordia spinescens* | -83.96 | 9.95 |
| *Cordia spinescens* | -82.93 | 8.95 |
| *Cordia spinescens* | -82.83 | 8.94 |
| *Cordia spinescens* | -84.48 | 10.15 |
| *Cordia spinescens* | -82.95 | 8.76 |
| *Cordia spinescens* | -83.95 | 9.73 |
| *Cordia spinescens* | -84.06 | 10.10 |
| *Cordia spinescens* | -82.85 | 8.93 |
| *Cordia spinescens* | -82.96 | 8.78 |
| *Cordia spinescens* | -84.10 | 9.86 |
| *Cordia spinescens* | -84.47 | 9.95 |
| *Cordia spinescens* | -81.58 | 7.80 |
| *Cordia spinescens* | -81.90 | 7.23 |
| *Cordia spinescens* | -81.73 | 7.61 |
| *Cordia spinescens* | -81.73 | 7.44 |
| *Cordia spinescens* | -81.75 | 7.48 |
| *Cordia spinescens* | -81.73 | 7.63 |
| *Cordia spinescens* | -83.22 | 8.68 |
| *Cordia spinescens* | -54.97 | -9.00 |
| *Melanthera nivea* | -105.43 | 20.87 |
| *Melanthera nivea* | -105.43 | 20.88 |
| *Melanthera nivea* | -105.42 | 20.87 |
| *Melanthera nivea* | -105.41 | 20.84 |
| *Melanthera nivea* | -105.32 | 20.95 |
| *Melanthera nivea* | -105.29 | 20.47 |
| *Melanthera nivea* | -105.29 | 20.45 |
| *Melanthera nivea* | -105.29 | 20.45 |
| *Melanthera nivea* | -105.27 | 21.62 |
| *Melanthera nivea* | -105.23 | 20.63 |
| *Melanthera nivea* | -105.07 | 19.53 |
| *Melanthera nivea* | -105.05 | 19.52 |
| *Melanthera nivea* | -105.05 | 19.52 |
| *Melanthera nivea* | -105.05 | 19.52 |
| *Melanthera nivea* | -105.05 | 19.50 |
| *Melanthera nivea* | -105.04 | 19.51 |
| *Melanthera nivea* | -105.04 | 19.51 |
| *Melanthera nivea* | -105.03 | 19.45 |
| *Melanthera nivea* | -105.03 | 19.55 |
| *Melanthera nivea* | -105.00 | 21.21 |
| *Melanthera nivea* | -104.96 | 19.41 |
| *Melanthera nivea* | -104.96 | 19.42 |
| *Melanthera nivea* | -104.96 | 19.43 |
| *Melanthera nivea* | -104.96 | 19.44 |
| *Melanthera nivea* | -104.95 | 19.42 |
| *Melanthera nivea* | -104.95 | 19.42 |
| *Melanthera nivea* | -104.95 | 19.42 |
| *Melanthera nivea* | -104.95 | 19.43 |
| *Melanthera nivea* | -104.95 | 19.43 |
| *Melanthera nivea* | -104.95 | 19.43 |
| *Melanthera nivea* | -104.95 | 19.42 |
| *Melanthera nivea* | -104.29 | 19.77 |
| *Melanthera nivea* | -104.15 | 19.79 |
| *Melanthera nivea* | -103.69 | 24.83 |
| *Melanthera nivea* | -103.50 | 18.60 |
| *Melanthera nivea* | -103.50 | 18.59 |
| *Melanthera nivea* | -102.05 | 19.03 |
| *Melanthera nivea* | -102.05 | 19.02 |
| *Melanthera nivea* | -102.00 | 23.00 |
| *Melanthera nivea* | -101.90 | 19.31 |
| *Melanthera nivea* | -101.84 | 18.04 |
| *Melanthera nivea* | -101.84 | 18.04 |
| *Melanthera nivea* | -101.79 | 17.84 |
| *Melanthera nivea* | -101.60 | 17.98 |
| *Melanthera nivea* | -101.17 | 22.05 |
| *Melanthera nivea* | -100.37 | 18.69 |
| *Melanthera nivea* | -100.36 | 18.69 |
| *Melanthera nivea* | -99.75 | 21.50 |
| *Melanthera nivea* | -99.39 | 22.60 |
| *Melanthera nivea* | -99.26 | 21.98 |
| *Melanthera nivea* | -99.10 | 21.22 |
| *Melanthera nivea* | -99.10 | 21.22 |
| *Melanthera nivea* | -99.06 | 21.28 |
| *Melanthera nivea* | -99.06 | 21.27 |
| *Melanthera nivea* | -99.06 | 21.28 |
| *Melanthera nivea* | -99.05 | 21.25 |
| *Melanthera nivea* | -99.05 | 21.25 |
| *Melanthera nivea* | -99.05 | 21.27 |
| *Melanthera nivea* | -99.05 | 21.27 |
| *Melanthera nivea* | -99.04 | 21.26 |
| *Melanthera nivea* | -99.00 | 21.54 |
| *Melanthera nivea* | -99.00 | 21.54 |
| *Melanthera nivea* | -98.99 | 21.50 |
| *Melanthera nivea* | -98.98 | 21.47 |
| *Melanthera nivea* | -98.97 | 21.61 |
| *Melanthera nivea* | -98.96 | 21.42 |
| *Melanthera nivea* | -98.96 | 21.61 |
| *Melanthera nivea* | -98.91 | 21.64 |
| *Melanthera nivea* | -98.90 | 21.61 |
| *Melanthera nivea* | -98.89 | 21.63 |
| *Melanthera nivea* | -98.82 | 18.12 |
| *Melanthera nivea* | -98.80 | 21.27 |
| *Melanthera nivea* | -98.75 | 21.25 |
| *Melanthera nivea* | -98.49 | 22.16 |
| *Melanthera nivea* | -98.42 | 22.13 |
| *Melanthera nivea* | -98.38 | 20.70 |
| *Melanthera nivea* | -98.33 | 20.43 |
| *Melanthera nivea* | -98.23 | 21.34 |
| *Melanthera nivea* | -98.23 | 21.35 |
| *Melanthera nivea* | -98.22 | 20.93 |
| *Melanthera nivea* | -98.21 | 20.94 |
| *Melanthera nivea* | -98.20 | 20.93 |
| *Melanthera nivea* | -98.19 | 20.93 |
| *Melanthera nivea* | -98.19 | 20.93 |
| *Melanthera nivea* | -98.05 | 22.91 |
| *Melanthera nivea* | -97.98 | 20.37 |
| *Melanthera nivea* | -97.98 | 20.37 |
| *Melanthera nivea* | -97.98 | 20.37 |
| *Melanthera nivea* | -97.98 | 20.37 |
| *Melanthera nivea* | -97.98 | 22.28 |
| *Melanthera nivea* | -97.97 | 22.27 |
| *Melanthera nivea* | -97.97 | 22.27 |
| *Melanthera nivea* | -97.93 | 20.27 |
| *Melanthera nivea* | -97.93 | 17.03 |
| *Melanthera nivea* | -97.93 | 17.02 |
| *Melanthera nivea* | -97.92 | 17.02 |
| *Melanthera nivea* | -97.89 | 20.39 |
| *Melanthera nivea* | -97.89 | 22.25 |
| *Melanthera nivea* | -97.89 | 22.25 |
| *Melanthera nivea* | -97.89 | 22.24 |
| *Melanthera nivea* | -97.89 | 22.24 |
| *Melanthera nivea* | -97.87 | 22.26 |
| *Melanthera nivea* | -97.86 | 22.23 |
| *Melanthera nivea* | -97.86 | 22.23 |
| *Melanthera nivea* | -97.85 | 20.25 |
| *Melanthera nivea* | -97.85 | 20.25 |
| *Melanthera nivea* | -97.85 | 20.25 |
| *Melanthera nivea* | -97.84 | 22.26 |
| *Melanthera nivea* | -97.83 | 22.25 |
| *Melanthera nivea* | -97.81 | 21.23 |
| *Melanthera nivea* | -97.80 | 22.25 |
| *Melanthera nivea* | -97.80 | 20.25 |
| *Melanthera nivea* | -97.79 | 20.07 |
| *Melanthera nivea* | -97.75 | 20.05 |
| *Melanthera nivea* | -97.73 | 20.13 |
| *Melanthera nivea* | -97.73 | 20.13 |
| *Melanthera nivea* | -97.67 | 19.97 |
| *Melanthera nivea* | -97.61 | 20.06 |
| *Melanthera nivea* | -97.61 | 20.06 |
| *Melanthera nivea* | -97.58 | 20.14 |
| *Melanthera nivea* | -97.58 | 20.14 |
| *Melanthera nivea* | -97.57 | 20.16 |
| *Melanthera nivea* | -97.53 | 20.04 |
| *Melanthera nivea* | -97.51 | 20.09 |
| *Melanthera nivea* | -97.51 | 20.04 |
| *Melanthera nivea* | -97.47 | 20.54 |
| *Melanthera nivea* | -97.47 | 20.55 |
| *Melanthera nivea* | -97.43 | 20.57 |
| *Melanthera nivea* | -97.43 | 20.02 |
| *Melanthera nivea* | -97.32 | 20.03 |
| *Melanthera nivea* | -97.31 | 20.70 |
| *Melanthera nivea* | -97.31 | 20.70 |
| *Melanthera nivea* | -97.24 | 19.93 |
| *Melanthera nivea* | -97.23 | 20.02 |
| *Melanthera nivea* | -97.23 | 19.97 |
| *Melanthera nivea* | -97.22 | 19.92 |
| *Melanthera nivea* | -97.22 | 19.97 |
| *Melanthera nivea* | -97.22 | 19.93 |
| *Melanthera nivea* | -97.22 | 19.93 |
| *Melanthera nivea* | -97.22 | 19.92 |
| *Melanthera nivea* | -97.22 | 19.92 |
| *Melanthera nivea* | -97.22 | 19.93 |
| *Melanthera nivea* | -97.21 | 19.96 |
| *Melanthera nivea* | -97.21 | 19.96 |
| *Melanthera nivea* | -97.19 | 20.08 |
| *Melanthera nivea* | -97.19 | 19.10 |
| *Melanthera nivea* | -97.14 | 20.44 |
| *Melanthera nivea* | -97.14 | 20.44 |
| *Melanthera nivea* | -97.13 | 18.82 |
| *Melanthera nivea* | -97.11 | 18.85 |
| *Melanthera nivea* | -97.11 | 18.85 |
| *Melanthera nivea* | -97.11 | 18.85 |
| *Melanthera nivea* | -97.10 | 18.85 |
| *Melanthera nivea* | -97.10 | 18.85 |
| *Melanthera nivea* | -97.10 | 18.85 |
| *Melanthera nivea* | -97.10 | 16.17 |
| *Melanthera nivea* | -97.09 | 18.86 |
| *Melanthera nivea* | -97.08 | 18.91 |
| *Melanthera nivea* | -97.08 | 18.85 |
| *Melanthera nivea* | -97.08 | 18.92 |
| *Melanthera nivea* | -97.07 | 18.87 |
| *Melanthera nivea* | -97.07 | 18.93 |
| *Melanthera nivea* | -97.07 | 18.93 |
| *Melanthera nivea* | -97.05 | 18.88 |
| *Melanthera nivea* | -97.02 | 18.96 |
| *Melanthera nivea* | -97.01 | 19.11 |
| *Melanthera nivea* | -97.01 | 18.91 |
| *Melanthera nivea* | -97.00 | 18.88 |
| *Melanthera nivea* | -97.00 | 18.88 |
| *Melanthera nivea* | -97.00 | 20.05 |
| *Melanthera nivea* | -97.00 | 18.67 |
| *Melanthera nivea* | -97.00 | 19.40 |
| *Melanthera nivea* | -97.00 | 19.45 |
| *Melanthera nivea* | -97.00 | 19.40 |
| *Melanthera nivea* | -96.99 | 19.40 |
| *Melanthera nivea* | -96.99 | 19.40 |
| *Melanthera nivea* | -96.99 | 20.05 |
| *Melanthera nivea* | -96.98 | 18.88 |
| *Melanthera nivea* | -96.98 | 18.90 |
| *Melanthera nivea* | -96.97 | 19.39 |
| *Melanthera nivea* | -96.97 | 19.39 |
| *Melanthera nivea* | -96.96 | 18.81 |
| *Melanthera nivea* | -96.96 | 18.81 |
| *Melanthera nivea* | -96.96 | 19.53 |
| *Melanthera nivea* | -96.96 | 19.32 |
| *Melanthera nivea* | -96.96 | 19.32 |
| *Melanthera nivea* | -96.96 | 19.32 |
| *Melanthera nivea* | -96.95 | 19.48 |
| *Melanthera nivea* | -96.95 | 19.48 |
| *Melanthera nivea* | -96.95 | 19.43 |
| *Melanthera nivea* | -96.95 | 18.90 |
| *Melanthera nivea* | -96.95 | 19.47 |
| *Melanthera nivea* | -96.95 | 19.47 |
| *Melanthera nivea* | -96.95 | 19.47 |
| *Melanthera nivea* | -96.95 | 19.50 |
| *Melanthera nivea* | -96.95 | 18.89 |
| *Melanthera nivea* | -96.95 | 18.89 |
| *Melanthera nivea* | -96.95 | 18.89 |
| *Melanthera nivea* | -96.94 | 19.51 |
| *Melanthera nivea* | -96.94 | 19.52 |
| *Melanthera nivea* | -96.94 | 19.51 |
| *Melanthera nivea* | -96.94 | 19.54 |
| *Melanthera nivea* | -96.93 | 19.40 |
| *Melanthera nivea* | -96.93 | 19.40 |
| *Melanthera nivea* | -96.93 | 18.89 |
| *Melanthera nivea* | -96.93 | 18.88 |
| *Melanthera nivea* | -96.93 | 19.54 |
| *Melanthera nivea* | -96.93 | 19.54 |
| *Melanthera nivea* | -96.92 | 18.90 |
| *Melanthera nivea* | -96.92 | 18.90 |
| *Melanthera nivea* | -96.92 | 18.88 |
| *Melanthera nivea* | -96.92 | 19.61 |
| *Melanthera nivea* | -96.92 | 19.61 |
| *Melanthera nivea* | -96.91 | 18.85 |
| *Melanthera nivea* | -96.91 | 18.66 |
| *Melanthera nivea* | -96.90 | 18.66 |
| *Melanthera nivea* | -96.90 | 19.50 |
| *Melanthera nivea* | -96.90 | 19.61 |
| *Melanthera nivea* | -96.90 | 19.61 |
| *Melanthera nivea* | -96.89 | 19.60 |
| *Melanthera nivea* | -96.89 | 18.86 |
| *Melanthera nivea* | -96.89 | 19.38 |
| *Melanthera nivea* | -96.89 | 18.86 |
| *Melanthera nivea* | -96.89 | 20.09 |
| *Melanthera nivea* | -96.89 | 19.61 |
| *Melanthera nivea* | -96.89 | 19.61 |
| *Melanthera nivea* | -96.88 | 19.27 |
| *Melanthera nivea* | -96.88 | 19.37 |
| *Melanthera nivea* | -96.88 | 19.27 |
| *Melanthera nivea* | -96.88 | 19.52 |
| *Melanthera nivea* | -96.88 | 20.20 |
| *Melanthera nivea* | -96.87 | 18.77 |
| *Melanthera nivea* | -96.87 | 18.77 |
| *Melanthera nivea* | -96.87 | 18.85 |
| *Melanthera nivea* | -96.87 | 20.20 |
| *Melanthera nivea* | -96.87 | 19.27 |
| *Melanthera nivea* | -96.86 | 19.51 |
| *Melanthera nivea* | -96.86 | 18.86 |
| *Melanthera nivea* | -96.85 | 18.84 |
| *Melanthera nivea* | -96.85 | 18.84 |
| *Melanthera nivea* | -96.85 | 18.84 |
| *Melanthera nivea* | -96.85 | 18.84 |
| *Melanthera nivea* | -96.84 | 18.83 |
| *Melanthera nivea* | -96.84 | 18.83 |
| *Melanthera nivea* | -96.84 | 18.88 |
| *Melanthera nivea* | -96.84 | 18.88 |
| *Melanthera nivea* | -96.83 | 19.45 |
| *Melanthera nivea* | -96.83 | 19.49 |
| *Melanthera nivea* | -96.83 | 19.49 |
| *Melanthera nivea* | -96.82 | 19.21 |
| *Melanthera nivea* | -96.82 | 19.22 |
| *Melanthera nivea* | -96.82 | 19.48 |
| *Melanthera nivea* | -96.82 | 19.21 |
| *Melanthera nivea* | -96.81 | 19.48 |
| *Melanthera nivea* | -96.80 | 18.76 |
| *Melanthera nivea* | -96.80 | 18.76 |
| *Melanthera nivea* | -96.80 | 19.47 |
| *Melanthera nivea* | -96.80 | 18.96 |
| *Melanthera nivea* | -96.80 | 20.25 |
| *Melanthera nivea* | -96.80 | 20.25 |
| *Melanthera nivea* | -96.79 | 18.53 |
| *Melanthera nivea* | -96.78 | 18.95 |
| *Melanthera nivea* | -96.78 | 18.83 |
| *Melanthera nivea* | -96.78 | 18.91 |
| *Melanthera nivea* | -96.78 | 20.23 |
| *Melanthera nivea* | -96.78 | 18.93 |
| *Melanthera nivea* | -96.77 | 18.82 |
| *Melanthera nivea* | -96.77 | 18.82 |
| *Melanthera nivea* | -96.77 | 18.82 |
| *Melanthera nivea* | -96.77 | 18.92 |
| *Melanthera nivea* | -96.77 | 20.22 |
| *Melanthera nivea* | -96.77 | 18.85 |
| *Melanthera nivea* | -96.75 | 18.64 |
| *Melanthera nivea* | -96.74 | 19.91 |
| *Melanthera nivea* | -96.74 | 18.64 |
| *Melanthera nivea* | -96.73 | 19.90 |
| *Melanthera nivea* | -96.73 | 19.90 |
| *Melanthera nivea* | -96.73 | 18.64 |
| *Melanthera nivea* | -96.72 | 18.38 |
| *Melanthera nivea* | -96.71 | 19.89 |
| *Melanthera nivea* | -96.69 | 19.23 |
| *Melanthera nivea* | -96.68 | 19.22 |
| *Melanthera nivea* | -96.67 | 17.86 |
| *Melanthera nivea* | -96.67 | 18.67 |
| *Melanthera nivea* | -96.67 | 18.67 |
| *Melanthera nivea* | -96.67 | 19.22 |
| *Melanthera nivea* | -96.66 | 18.42 |
| *Melanthera nivea* | -96.65 | 18.43 |
| *Melanthera nivea* | -96.65 | 18.51 |
| *Melanthera nivea* | -96.61 | 19.76 |
| *Melanthera nivea* | -96.61 | 19.76 |
| *Melanthera nivea* | -96.59 | 19.18 |
| *Melanthera nivea* | -96.59 | 19.21 |
| *Melanthera nivea* | -96.56 | 19.79 |
| *Melanthera nivea* | -96.55 | 19.25 |
| *Melanthera nivea* | -96.55 | 19.78 |
| *Melanthera nivea* | -96.50 | 17.75 |
| *Melanthera nivea* | -96.49 | 19.49 |
| *Melanthera nivea* | -96.49 | 19.34 |
| *Melanthera nivea* | -96.48 | 19.48 |
| *Melanthera nivea* | -96.46 | 18.23 |
| *Melanthera nivea* | -96.42 | 17.67 |
| *Melanthera nivea* | -96.41 | 19.53 |
| *Melanthera nivea* | -96.41 | 19.53 |
| *Melanthera nivea* | -96.41 | 18.23 |
| *Melanthera nivea* | -96.40 | 18.23 |
| *Melanthera nivea* | -96.40 | 19.24 |
| *Melanthera nivea* | -96.39 | 19.27 |
| *Melanthera nivea* | -96.39 | 19.27 |
| *Melanthera nivea* | -96.39 | 19.27 |
| *Melanthera nivea* | -96.37 | 17.63 |
| *Melanthera nivea* | -96.37 | 17.98 |
| *Melanthera nivea* | -96.36 | 15.81 |
| *Melanthera nivea* | -96.36 | 19.34 |
| *Melanthera nivea* | -96.36 | 19.34 |
| *Melanthera nivea* | -96.36 | 15.81 |
| *Melanthera nivea* | -96.35 | 17.62 |
| *Melanthera nivea* | -96.35 | 17.62 |
| *Melanthera nivea* | -96.35 | 18.22 |
| *Melanthera nivea* | -96.34 | 18.22 |
| *Melanthera nivea* | -96.33 | 17.74 |
| *Melanthera nivea* | -96.33 | 19.32 |
| *Melanthera nivea* | -96.32 | 19.32 |
| *Melanthera nivea* | -96.32 | 19.32 |
| *Melanthera nivea* | -96.32 | 17.78 |
| *Melanthera nivea* | -96.32 | 17.75 |
| *Melanthera nivea* | -96.31 | 17.72 |
| *Melanthera nivea* | -96.31 | 17.70 |
| *Melanthera nivea* | -96.30 | 18.11 |
| *Melanthera nivea* | -96.30 | 19.20 |
| *Melanthera nivea* | -96.30 | 19.18 |
| *Melanthera nivea* | -96.28 | 17.68 |
| *Melanthera nivea* | -96.24 | 15.73 |
| *Melanthera nivea* | -96.23 | 15.73 |
| *Melanthera nivea* | -96.23 | 15.73 |
| *Melanthera nivea* | -96.23 | 15.73 |
| *Melanthera nivea* | -96.23 | 18.29 |
| *Melanthera nivea* | -96.22 | 18.28 |
| *Melanthera nivea* | -96.22 | 18.28 |
| *Melanthera nivea* | -96.22 | 18.28 |
| *Melanthera nivea* | -96.21 | 15.76 |
| *Melanthera nivea* | -96.21 | 15.76 |
| *Melanthera nivea* | -96.21 | 15.76 |
| *Melanthera nivea* | -96.21 | 15.78 |
| *Melanthera nivea* | -96.21 | 15.78 |
| *Melanthera nivea* | -96.21 | 15.78 |
| *Melanthera nivea* | -96.21 | 17.86 |
| *Melanthera nivea* | -96.21 | 17.86 |
| *Melanthera nivea* | -96.19 | 15.84 |
| *Melanthera nivea* | -96.18 | 15.78 |
| *Melanthera nivea* | -96.18 | 15.78 |
| *Melanthera nivea* | -96.18 | 17.94 |
| *Melanthera nivea* | -96.18 | 15.78 |
| *Melanthera nivea* | -96.17 | 15.78 |
| *Melanthera nivea* | -96.17 | 15.77 |
| *Melanthera nivea* | -96.17 | 18.02 |
| *Melanthera nivea* | -96.17 | 17.93 |
| *Melanthera nivea* | -96.17 | 15.72 |
| *Melanthera nivea* | -96.17 | 15.72 |
| *Melanthera nivea* | -96.17 | 15.72 |
| *Melanthera nivea* | -96.15 | 18.08 |
| *Melanthera nivea* | -96.15 | 17.94 |
| *Melanthera nivea* | -96.15 | 18.07 |
| *Melanthera nivea* | -96.14 | 18.14 |
| *Melanthera nivea* | -96.14 | 18.14 |
| *Melanthera nivea* | -96.14 | 18.14 |
| *Melanthera nivea* | -96.14 | 19.20 |
| *Melanthera nivea* | -96.13 | 19.17 |
| *Melanthera nivea* | -96.13 | 18.10 |
| *Melanthera nivea* | -96.10 | 15.77 |
| *Melanthera nivea* | -96.09 | 15.95 |
| *Melanthera nivea* | -96.06 | 15.78 |
| *Melanthera nivea* | -96.05 | 17.90 |
| *Melanthera nivea* | -96.03 | 17.25 |
| *Melanthera nivea* | -96.03 | 17.25 |
| *Melanthera nivea* | -96.03 | 17.25 |
| *Melanthera nivea* | -96.03 | 17.25 |
| *Melanthera nivea* | -96.03 | 17.88 |
| *Melanthera nivea* | -96.02 | 17.87 |
| *Melanthera nivea* | -96.00 | 17.25 |
| *Melanthera nivea* | -96.00 | 15.85 |
| *Melanthera nivea* | -95.98 | 15.81 |
| *Melanthera nivea* | -95.98 | 15.82 |
| *Melanthera nivea* | -95.97 | 15.84 |
| *Melanthera nivea* | -95.97 | 15.84 |
| *Melanthera nivea* | -95.97 | 15.84 |
| *Melanthera nivea* | -95.97 | 15.85 |
| *Melanthera nivea* | -95.97 | 15.83 |
| *Melanthera nivea* | -95.97 | 15.83 |
| *Melanthera nivea* | -95.97 | 15.83 |
| *Melanthera nivea* | -95.97 | 15.83 |
| *Melanthera nivea* | -95.97 | 15.84 |
| *Melanthera nivea* | -95.96 | 15.84 |
| *Melanthera nivea* | -95.96 | 15.83 |
| *Melanthera nivea* | -95.96 | 15.84 |
| *Melanthera nivea* | -95.96 | 15.84 |
| *Melanthera nivea* | -95.96 | 15.85 |
| *Melanthera nivea* | -95.96 | 15.85 |
| *Melanthera nivea* | -95.96 | 15.83 |
| *Melanthera nivea* | -95.96 | 15.83 |
| *Melanthera nivea* | -95.96 | 15.87 |
| *Melanthera nivea* | -95.96 | 15.86 |
| *Melanthera nivea* | -95.95 | 15.96 |
| *Melanthera nivea* | -95.92 | 15.87 |
| *Melanthera nivea* | -95.87 | 15.98 |
| *Melanthera nivea* | -95.82 | 17.77 |
| *Melanthera nivea* | -95.82 | 18.38 |
| *Melanthera nivea* | -95.77 | 17.47 |
| *Melanthera nivea* | -95.75 | 17.75 |
| *Melanthera nivea* | -95.71 | 18.41 |
| *Melanthera nivea* | -95.71 | 18.41 |
| *Melanthera nivea* | -95.37 | 18.59 |
| *Melanthera nivea* | -95.35 | 18.58 |
| *Melanthera nivea* | -95.29 | 18.46 |
| *Melanthera nivea* | -95.29 | 18.43 |
| *Melanthera nivea* | -95.28 | 18.45 |
| *Melanthera nivea* | -95.28 | 18.58 |
| *Melanthera nivea* | -95.24 | 16.95 |
| *Melanthera nivea* | -95.23 | 18.57 |
| *Melanthera nivea* | -95.23 | 16.95 |
| *Melanthera nivea* | -95.22 | 18.58 |
| *Melanthera nivea* | -95.21 | 16.35 |
| *Melanthera nivea* | -95.21 | 16.35 |
| *Melanthera nivea* | -95.21 | 18.45 |
| *Melanthera nivea* | -95.21 | 18.45 |
| *Melanthera nivea* | -95.20 | 18.44 |
| *Melanthera nivea* | -95.20 | 18.39 |
| *Melanthera nivea* | -95.20 | 18.39 |
| *Melanthera nivea* | -95.19 | 16.36 |
| *Melanthera nivea* | -95.18 | 18.43 |
| *Melanthera nivea* | -95.18 | 18.44 |
| *Melanthera nivea* | -95.18 | 18.43 |
| *Melanthera nivea* | -95.18 | 18.42 |
| *Melanthera nivea* | -95.18 | 18.43 |
| *Melanthera nivea* | -95.18 | 18.43 |
| *Melanthera nivea* | -95.16 | 18.46 |
| *Melanthera nivea* | -95.15 | 18.60 |
| *Melanthera nivea* | -95.15 | 18.40 |
| *Melanthera nivea* | -95.15 | 18.45 |
| *Melanthera nivea* | -95.15 | 18.57 |
| *Melanthera nivea* | -95.14 | 16.82 |
| *Melanthera nivea* | -95.13 | 17.47 |
| *Melanthera nivea* | -95.11 | 18.42 |
| *Melanthera nivea* | -95.11 | 18.45 |
| *Melanthera nivea* | -95.10 | 18.42 |
| *Melanthera nivea* | -95.10 | 18.42 |
| *Melanthera nivea* | -95.10 | 18.42 |
| *Melanthera nivea* | -95.09 | 18.41 |
| *Melanthera nivea* | -95.09 | 18.41 |
| *Melanthera nivea* | -95.09 | 18.41 |
| *Melanthera nivea* | -95.09 | 18.62 |
| *Melanthera nivea* | -95.08 | 18.45 |
| *Melanthera nivea* | -95.08 | 18.58 |
| *Melanthera nivea* | -95.07 | 18.58 |
| *Melanthera nivea* | -95.07 | 18.44 |
| *Melanthera nivea* | -95.07 | 18.59 |
| *Melanthera nivea* | -95.07 | 18.57 |
| *Melanthera nivea* | -95.07 | 18.58 |
| *Melanthera nivea* | -95.06 | 18.58 |
| *Melanthera nivea* | -95.03 | 18.53 |
| *Melanthera nivea* | -94.96 | 16.53 |
| *Melanthera nivea* | -94.94 | 16.56 |
| *Melanthera nivea* | -94.92 | 16.45 |
| *Melanthera nivea* | -94.92 | 17.10 |
| *Melanthera nivea* | -94.91 | 16.68 |
| *Melanthera nivea* | -94.90 | 16.67 |
| *Melanthera nivea* | -94.89 | 18.26 |
| *Melanthera nivea* | -94.88 | 18.25 |
| *Melanthera nivea* | -94.76 | 17.73 |
| *Melanthera nivea* | -94.76 | 17.77 |
| *Melanthera nivea* | -94.75 | 17.72 |
| *Melanthera nivea* | -94.69 | 16.90 |
| *Melanthera nivea* | -94.61 | 17.26 |
| *Melanthera nivea* | -94.61 | 17.26 |
| *Melanthera nivea* | -94.61 | 17.25 |
| *Melanthera nivea* | -94.58 | 17.25 |
| *Melanthera nivea* | -94.57 | 16.37 |
| *Melanthera nivea* | -94.56 | 18.12 |
| *Melanthera nivea* | -94.55 | 18.12 |
| *Melanthera nivea* | -94.53 | 17.26 |
| *Melanthera nivea* | -94.52 | 17.27 |
| *Melanthera nivea* | -94.50 | 17.27 |
| *Melanthera nivea* | -94.50 | 17.28 |
| *Melanthera nivea* | -94.50 | 17.28 |
| *Melanthera nivea* | -94.37 | 17.27 |
| *Melanthera nivea* | -94.37 | 17.35 |
| *Melanthera nivea* | -94.22 | 18.06 |
| *Melanthera nivea* | -94.21 | 17.84 |
| *Melanthera nivea* | -94.20 | 17.83 |
| *Melanthera nivea* | -94.15 | 16.73 |
| *Melanthera nivea* | -94.15 | 16.73 |
| *Melanthera nivea* | -94.15 | 16.67 |
| *Melanthera nivea* | -94.15 | 16.73 |
| *Melanthera nivea* | -94.15 | 16.67 |
| *Melanthera nivea* | -94.15 | 16.73 |
| *Melanthera nivea* | -94.15 | 16.71 |
| *Melanthera nivea* | -94.15 | 16.74 |
| *Melanthera nivea* | -94.15 | 16.70 |
| *Melanthera nivea* | -94.15 | 16.71 |
| *Melanthera nivea* | -94.14 | 16.68 |
| *Melanthera nivea* | -94.14 | 16.70 |
| *Melanthera nivea* | -94.14 | 16.72 |
| *Melanthera nivea* | -94.14 | 16.71 |
| *Melanthera nivea* | -94.13 | 16.72 |
| *Melanthera nivea* | -94.13 | 16.07 |
| *Melanthera nivea* | -94.13 | 16.70 |
| *Melanthera nivea* | -94.13 | 16.70 |
| *Melanthera nivea* | -94.13 | 16.72 |
| *Melanthera nivea* | -94.13 | 16.71 |
| *Melanthera nivea* | -94.11 | 17.09 |
| *Melanthera nivea* | -94.11 | 17.09 |
| *Melanthera nivea* | -94.11 | 17.96 |
| *Melanthera nivea* | -94.10 | 18.05 |
| *Melanthera nivea* | -94.10 | 17.95 |
| *Melanthera nivea* | -94.07 | 16.68 |
| *Melanthera nivea* | -94.04 | 16.68 |
| *Melanthera nivea* | -94.02 | 16.47 |
| *Melanthera nivea* | -93.97 | 16.67 |
| *Melanthera nivea* | -93.94 | 16.64 |
| *Melanthera nivea* | -93.93 | 16.52 |
| *Melanthera nivea* | -93.87 | 16.06 |
| *Melanthera nivea* | -93.87 | 16.05 |
| *Melanthera nivea* | -93.86 | 16.31 |
| *Melanthera nivea* | -93.77 | 17.68 |
| *Melanthera nivea* | -93.77 | 16.98 |
| *Melanthera nivea* | -93.74 | 16.15 |
| *Melanthera nivea* | -93.71 | 16.94 |
| *Melanthera nivea* | -93.67 | 16.26 |
| *Melanthera nivea* | -93.66 | 16.25 |
| *Melanthera nivea* | -93.62 | 17.01 |
| *Melanthera nivea* | -93.62 | 15.94 |
| *Melanthera nivea* | -93.60 | 17.95 |
| *Melanthera nivea* | -93.60 | 15.92 |
| *Melanthera nivea* | -93.59 | 16.90 |
| *Melanthera nivea* | -93.59 | 18.00 |
| *Melanthera nivea* | -93.58 | 17.98 |
| *Melanthera nivea* | -93.57 | 17.97 |
| *Melanthera nivea* | -93.55 | 18.00 |
| *Melanthera nivea* | -93.55 | 18.00 |
| *Melanthera nivea* | -93.51 | 17.51 |
| *Melanthera nivea* | -93.49 | 16.79 |
| *Melanthera nivea* | -93.48 | 17.00 |
| *Melanthera nivea* | -93.48 | 15.99 |
| *Melanthera nivea* | -93.48 | 15.99 |
| *Melanthera nivea* | -93.47 | 18.00 |
| *Melanthera nivea* | -93.47 | 18.00 |
| *Melanthera nivea* | -93.41 | 16.82 |
| *Melanthera nivea* | -93.39 | 18.20 |
| *Melanthera nivea* | -93.39 | 18.21 |
| *Melanthera nivea* | -93.38 | 18.20 |
| *Melanthera nivea* | -93.37 | 18.20 |
| *Melanthera nivea* | -93.36 | 16.77 |
| *Melanthera nivea* | -93.36 | 18.20 |
| *Melanthera nivea* | -93.34 | 16.80 |
| *Melanthera nivea* | -93.33 | 17.92 |
| *Melanthera nivea* | -93.29 | 17.99 |
| *Melanthera nivea* | -93.29 | 18.00 |
| *Melanthera nivea* | -93.28 | 18.01 |
| *Melanthera nivea* | -93.28 | 18.01 |
| *Melanthera nivea* | -93.28 | 16.20 |
| *Melanthera nivea* | -93.28 | 17.99 |
| *Melanthera nivea* | -93.27 | 16.80 |
| *Melanthera nivea* | -93.27 | 16.80 |
| *Melanthera nivea* | -93.27 | 16.23 |
| *Melanthera nivea* | -93.26 | 16.80 |
| *Melanthera nivea* | -93.25 | 15.92 |
| *Melanthera nivea* | -93.25 | 15.92 |
| *Melanthera nivea* | -93.22 | 16.78 |
| *Melanthera nivea* | -93.21 | 18.15 |
| *Melanthera nivea* | -93.21 | 18.36 |
| *Melanthera nivea* | -93.19 | 16.81 |
| *Melanthera nivea* | -93.17 | 16.75 |
| *Melanthera nivea* | -93.17 | 16.76 |
| *Melanthera nivea* | -93.17 | 16.88 |
| *Melanthera nivea* | -93.15 | 15.74 |
| *Melanthera nivea* | -93.14 | 17.52 |
| *Melanthera nivea* | -93.13 | 18.25 |
| *Melanthera nivea* | -93.12 | 16.90 |
| *Melanthera nivea* | -93.12 | 18.24 |
| *Melanthera nivea* | -93.12 | 17.91 |
| *Melanthera nivea* | -93.12 | 16.71 |
| *Melanthera nivea* | -93.12 | 16.78 |
| *Melanthera nivea* | -93.12 | 16.75 |
| *Melanthera nivea* | -93.12 | 16.75 |
| *Melanthera nivea* | -93.12 | 16.75 |
| *Melanthera nivea* | -93.12 | 16.84 |
| *Melanthera nivea* | -93.11 | 17.90 |
| *Melanthera nivea* | -93.11 | 16.73 |
| *Melanthera nivea* | -93.11 | 18.22 |
| *Melanthera nivea* | -93.10 | 16.78 |
| *Melanthera nivea* | -93.10 | 16.91 |
| *Melanthera nivea* | -93.10 | 16.75 |
| *Melanthera nivea* | -93.10 | 16.67 |
| *Melanthera nivea* | -93.10 | 16.73 |
| *Melanthera nivea* | -93.10 | 16.72 |
| *Melanthera nivea* | -93.10 | 16.94 |
| *Melanthera nivea* | -93.09 | 17.43 |
| *Melanthera nivea* | -93.09 | 16.67 |
| *Melanthera nivea* | -93.09 | 17.44 |
| *Melanthera nivea* | -93.09 | 16.81 |
| *Melanthera nivea* | -93.09 | 16.83 |
| *Melanthera nivea* | -93.08 | 16.83 |
| *Melanthera nivea* | -93.07 | 16.80 |
| *Melanthera nivea* | -93.05 | 15.80 |
| *Melanthera nivea* | -93.05 | 15.80 |
| *Melanthera nivea* | -93.05 | 15.77 |
| *Melanthera nivea* | -93.05 | 15.81 |
| *Melanthera nivea* | -93.04 | 17.42 |
| *Melanthera nivea* | -93.03 | 17.41 |
| *Melanthera nivea* | -93.03 | 17.40 |
| *Melanthera nivea* | -93.03 | 17.40 |
| *Melanthera nivea* | -93.03 | 17.25 |
| *Melanthera nivea* | -93.02 | 18.16 |
| *Melanthera nivea* | -93.02 | 17.27 |
| *Melanthera nivea* | -93.02 | 18.15 |
| *Melanthera nivea* | -93.02 | 17.22 |
| *Melanthera nivea* | -93.00 | 17.32 |
| *Melanthera nivea* | -93.00 | 17.32 |
| *Melanthera nivea* | -92.99 | 18.19 |
| *Melanthera nivea* | -92.99 | 18.19 |
| *Melanthera nivea* | -92.99 | 16.59 |
| *Melanthera nivea* | -92.99 | 18.19 |
| *Melanthera nivea* | -92.99 | 17.93 |
| *Melanthera nivea* | -92.98 | 16.54 |
| *Melanthera nivea* | -92.98 | 16.70 |
| *Melanthera nivea* | -92.98 | 16.04 |
| *Melanthera nivea* | -92.98 | 17.56 |
| *Melanthera nivea* | -92.98 | 16.60 |
| *Melanthera nivea* | -92.97 | 16.70 |
| *Melanthera nivea* | -92.97 | 17.55 |
| *Melanthera nivea* | -92.97 | 17.99 |
| *Melanthera nivea* | -92.95 | 18.02 |
| *Melanthera nivea* | -92.94 | 15.53 |
| *Melanthera nivea* | -92.93 | 16.10 |
| *Melanthera nivea* | -92.93 | 17.99 |
| *Melanthera nivea* | -92.93 | 15.49 |
| *Melanthera nivea* | -92.92 | 17.52 |
| *Melanthera nivea* | -92.92 | 15.47 |
| *Melanthera nivea* | -92.92 | 17.08 |
| *Melanthera nivea* | -92.91 | 16.79 |
| *Melanthera nivea* | -92.90 | 17.52 |
| *Melanthera nivea* | -92.90 | 16.74 |
| *Melanthera nivea* | -92.88 | 17.55 |
| *Melanthera nivea* | -92.83 | 17.73 |
| *Melanthera nivea* | -92.79 | 16.92 |
| *Melanthera nivea* | -92.78 | 16.96 |
| *Melanthera nivea* | -92.78 | 16.96 |
| *Melanthera nivea* | -92.78 | 16.96 |
| *Melanthera nivea* | -92.78 | 16.96 |
| *Melanthera nivea* | -92.78 | 16.96 |
| *Melanthera nivea* | -92.78 | 16.96 |
| *Melanthera nivea* | -92.77 | 16.92 |
| *Melanthera nivea* | -92.77 | 16.88 |
| *Melanthera nivea* | -92.77 | 16.88 |
| *Melanthera nivea* | -92.77 | 17.76 |
| *Melanthera nivea* | -92.77 | 16.89 |
| *Melanthera nivea* | -92.75 | 17.75 |
| *Melanthera nivea* | -92.72 | 15.91 |
| *Melanthera nivea* | -92.72 | 15.31 |
| *Melanthera nivea* | -92.71 | 16.43 |
| *Melanthera nivea* | -92.71 | 16.43 |
| *Melanthera nivea* | -92.67 | 15.34 |
| *Melanthera nivea* | -92.67 | 15.28 |
| *Melanthera nivea* | -92.66 | 18.34 |
| *Melanthera nivea* | -92.66 | 15.32 |
| *Melanthera nivea* | -92.65 | 18.34 |
| *Melanthera nivea* | -92.64 | 18.42 |
| *Melanthera nivea* | -92.64 | 18.42 |
| *Melanthera nivea* | -92.64 | 18.41 |
| *Melanthera nivea* | -92.64 | 18.37 |
| *Melanthera nivea* | -92.64 | 18.37 |
| *Melanthera nivea* | -92.64 | 18.37 |
| *Melanthera nivea* | -92.64 | 18.42 |
| *Melanthera nivea* | -92.64 | 18.44 |
| *Melanthera nivea* | -92.63 | 18.28 |
| *Melanthera nivea* | -92.63 | 18.28 |
| *Melanthera nivea* | -92.63 | 18.28 |
| *Melanthera nivea* | -92.63 | 18.42 |
| *Melanthera nivea* | -92.63 | 18.42 |
| *Melanthera nivea* | -92.63 | 18.27 |
| *Melanthera nivea* | -92.63 | 18.37 |
| *Melanthera nivea* | -92.63 | 18.37 |
| *Melanthera nivea* | -92.62 | 18.37 |
| *Melanthera nivea* | -92.62 | 18.37 |
| *Melanthera nivea* | -92.62 | 18.35 |
| *Melanthera nivea* | -92.62 | 18.35 |
| *Melanthera nivea* | -92.61 | 18.35 |
| *Melanthera nivea* | -92.61 | 18.37 |
| *Melanthera nivea* | -92.61 | 18.37 |
| *Melanthera nivea* | -92.61 | 18.37 |
| *Melanthera nivea* | -92.60 | 18.35 |
| *Melanthera nivea* | -92.60 | 17.72 |
| *Melanthera nivea* | -92.60 | 16.89 |
| *Melanthera nivea* | -92.59 | 17.72 |
| *Melanthera nivea* | -92.59 | 16.85 |
| *Melanthera nivea* | -92.59 | 17.72 |
| *Melanthera nivea* | -92.59 | 18.34 |
| *Melanthera nivea* | -92.58 | 18.60 |
| *Melanthera nivea* | -92.58 | 18.34 |
| *Melanthera nivea* | -92.58 | 18.60 |
| *Melanthera nivea* | -92.58 | 18.34 |
| *Melanthera nivea* | -92.58 | 18.45 |
| *Melanthera nivea* | -92.58 | 18.45 |
| *Melanthera nivea* | -92.57 | 18.44 |
| *Melanthera nivea* | -92.57 | 18.59 |
| *Melanthera nivea* | -92.57 | 18.45 |
| *Melanthera nivea* | -92.56 | 18.62 |
| *Melanthera nivea* | -92.56 | 18.62 |
| *Melanthera nivea* | -92.56 | 15.08 |
| *Melanthera nivea* | -92.56 | 16.96 |
| *Melanthera nivea* | -92.56 | 16.96 |
| *Melanthera nivea* | -92.56 | 18.39 |
| *Melanthera nivea* | -92.55 | 18.38 |
| *Melanthera nivea* | -92.53 | 16.87 |
| *Melanthera nivea* | -92.52 | 18.48 |
| *Melanthera nivea* | -92.52 | 18.48 |
| *Melanthera nivea* | -92.52 | 18.47 |
| *Melanthera nivea* | -92.52 | 18.48 |
| *Melanthera nivea* | -92.51 | 16.82 |
| *Melanthera nivea* | -92.51 | 16.82 |
| *Melanthera nivea* | -92.51 | 15.93 |
| *Melanthera nivea* | -92.50 | 17.72 |
| *Melanthera nivea* | -92.50 | 16.82 |
| *Melanthera nivea* | -92.49 | 17.66 |
| *Melanthera nivea* | -92.49 | 16.89 |
| *Melanthera nivea* | -92.49 | 16.89 |
| *Melanthera nivea* | -92.48 | 17.04 |
| *Melanthera nivea* | -92.48 | 14.92 |
| *Melanthera nivea* | -92.48 | 16.87 |
| *Melanthera nivea* | -92.48 | 16.87 |
| *Melanthera nivea* | -92.48 | 18.64 |
| *Melanthera nivea* | -92.48 | 15.21 |
| *Melanthera nivea* | -92.47 | 16.54 |
| *Melanthera nivea* | -92.47 | 16.87 |
| *Melanthera nivea* | -92.47 | 15.20 |
| *Melanthera nivea* | -92.47 | 15.14 |
| *Melanthera nivea* | -92.47 | 15.14 |
| *Melanthera nivea* | -92.45 | 14.86 |
| *Melanthera nivea* | -92.45 | 14.86 |
| *Melanthera nivea* | -92.44 | 17.98 |
| *Melanthera nivea* | -92.44 | 16.53 |
| *Melanthera nivea* | -92.44 | 16.50 |
| *Melanthera nivea* | -92.43 | 16.47 |
| *Melanthera nivea* | -92.43 | 16.49 |
| *Melanthera nivea* | -92.43 | 16.48 |
| *Melanthera nivea* | -92.42 | 18.36 |
| *Melanthera nivea* | -92.42 | 18.36 |
| *Melanthera nivea* | -92.41 | 16.48 |
| *Melanthera nivea* | -92.40 | 18.36 |
| *Melanthera nivea* | -92.39 | 16.79 |
| *Melanthera nivea* | -92.39 | 16.79 |
| *Melanthera nivea* | -92.38 | 16.86 |
| *Melanthera nivea* | -92.36 | 16.77 |
| *Melanthera nivea* | -92.36 | 18.21 |
| *Melanthera nivea* | -92.36 | 18.21 |
| *Melanthera nivea* | -92.36 | 16.11 |
| *Melanthera nivea* | -92.35 | 14.94 |
| *Melanthera nivea* | -92.35 | 15.31 |
| *Melanthera nivea* | -92.35 | 14.94 |
| *Melanthera nivea* | -92.35 | 14.94 |
| *Melanthera nivea* | -92.35 | 15.31 |
| *Melanthera nivea* | -92.35 | 18.20 |
| *Melanthera nivea* | -92.35 | 15.26 |
| *Melanthera nivea* | -92.35 | 18.21 |
| *Melanthera nivea* | -92.35 | 17.22 |
| *Melanthera nivea* | -92.35 | 17.23 |
| *Melanthera nivea* | -92.34 | 15.32 |
| *Melanthera nivea* | -92.34 | 18.23 |
| *Melanthera nivea* | -92.34 | 18.23 |
| *Melanthera nivea* | -92.33 | 18.23 |
| *Melanthera nivea* | -92.33 | 18.22 |
| *Melanthera nivea* | -92.33 | 17.28 |
| *Melanthera nivea* | -92.32 | 15.56 |
| *Melanthera nivea* | -92.32 | 14.84 |
| *Melanthera nivea* | -92.30 | 15.23 |
| *Melanthera nivea* | -92.28 | 14.88 |
| *Melanthera nivea* | -92.28 | 15.65 |
| *Melanthera nivea* | -92.28 | 16.82 |
| *Melanthera nivea* | -92.28 | 17.11 |
| *Melanthera nivea* | -92.27 | 16.82 |
| *Melanthera nivea* | -92.27 | 16.82 |
| *Melanthera nivea* | -92.27 | 17.10 |
| *Melanthera nivea* | -92.26 | 16.81 |
| *Melanthera nivea* | -92.26 | 14.91 |
| *Melanthera nivea* | -92.25 | 14.90 |
| *Melanthera nivea* | -92.18 | 14.76 |
| *Melanthera nivea* | -92.18 | 14.78 |
| *Melanthera nivea* | -92.18 | 14.78 |
| *Melanthera nivea* | -92.17 | 15.02 |
| *Melanthera nivea* | -92.17 | 17.17 |
| *Melanthera nivea* | -92.16 | 15.58 |
| *Melanthera nivea* | -92.16 | 14.99 |
| *Melanthera nivea* | -92.16 | 14.96 |
| *Melanthera nivea* | -92.15 | 14.95 |
| *Melanthera nivea* | -92.15 | 14.98 |
| *Melanthera nivea* | -92.14 | 15.06 |
| *Melanthera nivea* | -92.14 | 15.02 |
| *Melanthera nivea* | -92.14 | 15.58 |
| *Melanthera nivea* | -92.14 | 18.17 |
| *Melanthera nivea* | -92.13 | 15.02 |
| *Melanthera nivea* | -92.13 | 16.82 |
| *Melanthera nivea* | -92.12 | 18.21 |
| *Melanthera nivea* | -92.12 | 15.13 |
| *Melanthera nivea* | -92.11 | 15.04 |
| *Melanthera nivea* | -92.11 | 17.18 |
| *Melanthera nivea* | -92.10 | 18.15 |
| *Melanthera nivea* | -92.09 | 18.11 |
| *Melanthera nivea* | -92.08 | 17.22 |
| *Melanthera nivea* | -92.08 | 16.90 |
| *Melanthera nivea* | -92.07 | 15.03 |
| *Melanthera nivea* | -92.05 | 18.10 |
| *Melanthera nivea* | -92.03 | 17.76 |
| *Melanthera nivea* | -92.01 | 17.73 |
| *Melanthera nivea* | -92.01 | 17.73 |
| *Melanthera nivea* | -92.01 | 17.84 |
| *Melanthera nivea* | -92.01 | 17.73 |
| *Melanthera nivea* | -92.01 | 17.85 |
| *Melanthera nivea* | -92.00 | 17.72 |
| *Melanthera nivea* | -92.00 | 17.84 |
| *Melanthera nivea* | -91.99 | 17.39 |
| *Melanthera nivea* | -91.98 | 17.50 |
| *Melanthera nivea* | -91.98 | 16.32 |
| *Melanthera nivea* | -91.97 | 17.44 |
| *Melanthera nivea* | -91.97 | 17.46 |
| *Melanthera nivea* | -91.96 | 17.54 |
| *Melanthera nivea* | -91.96 | 18.00 |
| *Melanthera nivea* | -91.94 | 15.61 |
| *Melanthera nivea* | -91.90 | 18.05 |
| *Melanthera nivea* | -91.88 | 17.76 |
| *Melanthera nivea* | -91.85 | 18.37 |
| *Melanthera nivea* | -91.83 | 17.77 |
| *Melanthera nivea* | -91.81 | 17.75 |
| *Melanthera nivea* | -91.80 | 17.90 |
| *Melanthera nivea* | -91.79 | 17.75 |
| *Melanthera nivea* | -91.79 | 17.74 |
| *Melanthera nivea* | -91.79 | 17.75 |
| *Melanthera nivea* | -91.79 | 17.90 |
| *Melanthera nivea* | -91.79 | 18.64 |
| *Melanthera nivea* | -91.78 | 14.66 |
| *Melanthera nivea* | -91.75 | 14.81 |
| *Melanthera nivea* | -91.71 | 16.10 |
| *Melanthera nivea* | -91.71 | 16.10 |
| *Melanthera nivea* | -91.71 | 16.11 |
| *Melanthera nivea* | -91.70 | 16.11 |
| *Melanthera nivea* | -91.70 | 16.11 |
| *Melanthera nivea* | -91.66 | 16.27 |
| *Melanthera nivea* | -91.59 | 14.63 |
| *Melanthera nivea* | -91.51 | 18.42 |
| *Melanthera nivea* | -91.51 | 18.42 |
| *Melanthera nivea* | -91.47 | 18.46 |
| *Melanthera nivea* | -91.46 | 18.46 |
| *Melanthera nivea* | -91.37 | 18.90 |
| *Melanthera nivea* | -91.37 | 18.90 |
| *Melanthera nivea* | -91.37 | 17.29 |
| *Melanthera nivea* | -91.32 | 18.93 |
| *Melanthera nivea* | -91.32 | 18.93 |
| *Melanthera nivea* | -91.31 | 15.94 |
| *Melanthera nivea* | -91.26 | 18.96 |
| *Melanthera nivea* | -91.26 | 18.96 |
| *Melanthera nivea* | -91.25 | 16.94 |
| *Melanthera nivea* | -91.25 | 16.93 |
| *Melanthera nivea* | -91.18 | 17.13 |
| *Melanthera nivea* | -91.18 | 18.98 |
| *Melanthera nivea* | -91.18 | 18.98 |
| *Melanthera nivea* | -91.18 | 18.97 |
| *Melanthera nivea* | -91.18 | 18.97 |
| *Melanthera nivea* | -91.18 | 18.95 |
| *Melanthera nivea* | -91.18 | 18.95 |
| *Melanthera nivea* | -91.18 | 18.96 |
| *Melanthera nivea* | -91.18 | 18.96 |
| *Melanthera nivea* | -91.16 | 18.55 |
| *Melanthera nivea* | -91.15 | 18.99 |
| *Melanthera nivea* | -91.15 | 18.99 |
| *Melanthera nivea* | -91.14 | 17.78 |
| *Melanthera nivea* | -91.13 | 16.75 |
| *Melanthera nivea* | -91.13 | 17.77 |
| *Melanthera nivea* | -91.13 | 16.76 |
| *Melanthera nivea* | -91.13 | 16.76 |
| *Melanthera nivea* | -91.13 | 14.48 |
| *Melanthera nivea* | -91.13 | 16.76 |
| *Melanthera nivea* | -91.12 | 16.71 |
| *Melanthera nivea* | -91.12 | 16.71 |
| *Melanthera nivea* | -91.11 | 16.05 |
| *Melanthera nivea* | -91.11 | 16.05 |
| *Melanthera nivea* | -91.11 | 16.81 |
| *Melanthera nivea* | -91.10 | 16.76 |
| *Melanthera nivea* | -91.10 | 16.80 |
| *Melanthera nivea* | -91.10 | 19.02 |
| *Melanthera nivea* | -91.10 | 19.02 |
| *Melanthera nivea* | -91.09 | 19.03 |
| *Melanthera nivea* | -91.09 | 19.03 |
| *Melanthera nivea* | -91.09 | 19.02 |
| *Melanthera nivea* | -91.09 | 19.02 |
| *Melanthera nivea* | -91.08 | 16.74 |
| *Melanthera nivea* | -91.07 | 16.71 |
| *Melanthera nivea* | -91.05 | 18.19 |
| *Melanthera nivea* | -91.05 | 18.16 |
| *Melanthera nivea* | -91.03 | 16.77 |
| *Melanthera nivea* | -91.02 | 16.72 |
| *Melanthera nivea* | -91.01 | 16.76 |
| *Melanthera nivea* | -91.00 | 16.76 |
| *Melanthera nivea* | -90.99 | 16.76 |
| *Melanthera nivea* | -90.97 | 16.76 |
| *Melanthera nivea* | -90.97 | 16.90 |
| *Melanthera nivea* | -90.97 | 16.90 |
| *Melanthera nivea* | -90.96 | 16.10 |
| *Melanthera nivea* | -90.96 | 16.18 |
| *Melanthera nivea* | -90.94 | 16.15 |
| *Melanthera nivea* | -90.93 | 16.12 |
| *Melanthera nivea* | -90.93 | 16.79 |
| *Melanthera nivea* | -90.93 | 16.11 |
| *Melanthera nivea* | -90.93 | 16.12 |
| *Melanthera nivea* | -90.92 | 16.79 |
| *Melanthera nivea* | -90.91 | 16.73 |
| *Melanthera nivea* | -90.91 | 16.82 |
| *Melanthera nivea* | -90.91 | 16.80 |
| *Melanthera nivea* | -90.91 | 16.80 |
| *Melanthera nivea* | -90.90 | 16.80 |
| *Melanthera nivea* | -90.89 | 16.72 |
| *Melanthera nivea* | -90.87 | 16.71 |
| *Melanthera nivea* | -90.87 | 16.70 |
| *Melanthera nivea* | -90.86 | 17.91 |
| *Melanthera nivea* | -90.82 | 16.61 |
| *Melanthera nivea* | -90.82 | 16.66 |
| *Melanthera nivea* | -90.82 | 17.89 |
| *Melanthera nivea* | -90.82 | 16.66 |
| *Melanthera nivea* | -90.81 | 14.46 |
| *Melanthera nivea* | -90.80 | 16.65 |
| *Melanthera nivea* | -90.80 | 18.61 |
| *Melanthera nivea* | -90.77 | 16.63 |
| *Melanthera nivea* | -90.77 | 16.63 |
| *Melanthera nivea* | -90.76 | 16.61 |
| *Melanthera nivea* | -90.75 | 18.85 |
| *Melanthera nivea* | -90.74 | 18.77 |
| *Melanthera nivea* | -90.73 | 18.70 |
| *Melanthera nivea* | -90.73 | 18.75 |
| *Melanthera nivea* | -90.73 | 18.70 |
| *Melanthera nivea* | -90.73 | 18.90 |
| *Melanthera nivea* | -90.73 | 19.12 |
| *Melanthera nivea* | -90.73 | 18.61 |
| *Melanthera nivea* | -90.73 | 16.47 |
| *Melanthera nivea* | -90.73 | 16.47 |
| *Melanthera nivea* | -90.72 | 19.07 |
| *Melanthera nivea* | -90.72 | 19.32 |
| *Melanthera nivea* | -90.72 | 16.62 |
| *Melanthera nivea* | -90.72 | 19.07 |
| *Melanthera nivea* | -90.71 | 19.35 |
| *Melanthera nivea* | -90.70 | 16.58 |
| *Melanthera nivea* | -90.68 | 16.08 |
| *Melanthera nivea* | -90.65 | 16.46 |
| *Melanthera nivea* | -90.63 | 14.90 |
| *Melanthera nivea* | -90.62 | 14.87 |
| *Melanthera nivea* | -90.62 | 14.87 |
| *Melanthera nivea* | -90.61 | 19.28 |
| *Melanthera nivea* | -90.58 | 19.82 |
| *Melanthera nivea* | -90.58 | 19.82 |
| *Melanthera nivea* | -90.58 | 19.82 |
| *Melanthera nivea* | -90.57 | 16.10 |
| *Melanthera nivea* | -90.53 | 19.80 |
| *Melanthera nivea* | -90.53 | 19.80 |
| *Melanthera nivea* | -90.53 | 19.80 |
| *Melanthera nivea* | -90.51 | 14.57 |
| *Melanthera nivea* | -90.51 | 19.76 |
| *Melanthera nivea* | -90.51 | 19.79 |
| *Melanthera nivea* | -90.50 | 14.44 |
| *Melanthera nivea* | -90.50 | 19.79 |
| *Melanthera nivea* | -90.50 | 14.44 |
| *Melanthera nivea* | -90.49 | 19.79 |
| *Melanthera nivea* | -90.49 | 19.85 |
| *Melanthera nivea* | -90.49 | 16.11 |
| *Melanthera nivea* | -90.45 | 14.55 |
| *Melanthera nivea* | -90.45 | 20.69 |
| *Melanthera nivea* | -90.45 | 20.69 |
| *Melanthera nivea* | -90.44 | 19.80 |
| *Melanthera nivea* | -90.44 | 20.69 |
| *Melanthera nivea* | -90.44 | 19.80 |
| *Melanthera nivea* | -90.44 | 18.98 |
| *Melanthera nivea* | -90.42 | 15.68 |
| *Melanthera nivea* | -90.42 | 18.60 |
| *Melanthera nivea* | -90.42 | 20.50 |
| *Melanthera nivea* | -90.40 | 20.86 |
| *Melanthera nivea* | -90.40 | 20.87 |
| *Melanthera nivea* | -90.40 | 20.85 |
| *Melanthera nivea* | -90.40 | 20.86 |
| *Melanthera nivea* | -90.40 | 20.86 |
| *Melanthera nivea* | -90.40 | 20.86 |
| *Melanthera nivea* | -90.40 | 19.72 |
| *Melanthera nivea* | -90.40 | 19.93 |
| *Melanthera nivea* | -90.40 | 19.93 |
| *Melanthera nivea* | -90.39 | 20.85 |
| *Melanthera nivea* | -90.39 | 20.89 |
| *Melanthera nivea* | -90.39 | 20.89 |
| *Melanthera nivea* | -90.39 | 19.93 |
| *Melanthera nivea* | -90.39 | 20.89 |
| *Melanthera nivea* | -90.38 | 20.88 |
| *Melanthera nivea* | -90.38 | 19.92 |
| *Melanthera nivea* | -90.38 | 20.91 |
| *Melanthera nivea* | -90.38 | 20.89 |
| *Melanthera nivea* | -90.38 | 19.94 |
| *Melanthera nivea* | -90.38 | 19.94 |
| *Melanthera nivea* | -90.38 | 19.94 |
| *Melanthera nivea* | -90.38 | 19.94 |
| *Melanthera nivea* | -90.37 | 19.93 |
| *Melanthera nivea* | -90.37 | 20.53 |
| *Melanthera nivea* | -90.37 | 20.85 |
| *Melanthera nivea* | -90.37 | 20.85 |
| *Melanthera nivea* | -90.37 | 20.85 |
| *Melanthera nivea* | -90.36 | 20.98 |
| *Melanthera nivea* | -90.34 | 19.29 |
| *Melanthera nivea* | -90.34 | 19.72 |
| *Melanthera nivea* | -90.33 | 18.88 |
| *Melanthera nivea* | -90.33 | 19.13 |
| *Melanthera nivea* | -90.33 | 18.88 |
| *Melanthera nivea* | -90.32 | 15.10 |
| *Melanthera nivea* | -90.31 | 19.01 |
| *Melanthera nivea* | -90.31 | 19.01 |
| *Melanthera nivea* | -90.30 | 18.63 |
| *Melanthera nivea* | -90.29 | 19.13 |
| *Melanthera nivea* | -90.28 | 19.81 |
| *Melanthera nivea* | -90.28 | 20.88 |
| *Melanthera nivea* | -90.27 | 20.87 |
| *Melanthera nivea* | -90.26 | 20.01 |
| *Melanthera nivea* | -90.26 | 19.00 |
| *Melanthera nivea* | -90.25 | 19.00 |
| *Melanthera nivea* | -90.23 | 16.57 |
| *Melanthera nivea* | -90.23 | 18.61 |
| *Melanthera nivea* | -90.23 | 18.65 |
| *Melanthera nivea* | -90.23 | 20.86 |
| *Melanthera nivea* | -90.20 | 20.08 |
| *Melanthera nivea* | -90.20 | 20.08 |
| *Melanthera nivea* | -90.20 | 20.08 |
| *Melanthera nivea* | -90.18 | 19.53 |
| *Melanthera nivea* | -90.15 | 18.55 |
| *Melanthera nivea* | -90.14 | 18.63 |
| *Melanthera nivea* | -90.11 | 18.45 |
| *Melanthera nivea* | -90.09 | 21.15 |
| *Melanthera nivea* | -90.08 | 18.45 |
| *Melanthera nivea* | -90.07 | 21.16 |
| *Melanthera nivea* | -90.07 | 18.61 |
| *Melanthera nivea* | -90.07 | 18.61 |
| *Melanthera nivea* | -90.07 | 18.59 |
| *Melanthera nivea* | -90.07 | 13.80 |
| *Melanthera nivea* | -90.05 | 14.93 |
| *Melanthera nivea* | -90.04 | 21.16 |
| *Melanthera nivea* | -90.03 | 21.14 |
| *Melanthera nivea* | -90.00 | 16.83 |
| *Melanthera nivea* | -89.99 | 20.56 |
| *Melanthera nivea* | -89.99 | 20.56 |
| *Melanthera nivea* | -89.98 | 21.18 |
| *Melanthera nivea* | -89.96 | 20.15 |
| *Melanthera nivea* | -89.95 | 20.56 |
| *Melanthera nivea* | -89.95 | 20.15 |
| *Melanthera nivea* | -89.94 | 17.14 |
| *Melanthera nivea* | -89.93 | 13.82 |
| *Melanthera nivea* | -89.93 | 18.51 |
| *Melanthera nivea* | -89.92 | 20.18 |
| *Melanthera nivea* | -89.92 | 18.54 |
| *Melanthera nivea* | -89.92 | 18.50 |
| *Melanthera nivea* | -89.92 | 20.49 |
| *Melanthera nivea* | -89.91 | 18.54 |
| *Melanthera nivea* | -89.90 | 14.91 |
| *Melanthera nivea* | -89.90 | 18.53 |
| *Melanthera nivea* | -89.90 | 18.53 |
| *Melanthera nivea* | -89.90 | 18.52 |
| *Melanthera nivea* | -89.90 | 18.39 |
| *Melanthera nivea* | -89.90 | 18.54 |
| *Melanthera nivea* | -89.90 | 18.54 |
| *Melanthera nivea* | -89.90 | 18.54 |
| *Melanthera nivea* | -89.90 | 18.39 |
| *Melanthera nivea* | -89.89 | 18.36 |
| *Melanthera nivea* | -89.89 | 18.46 |
| *Melanthera nivea* | -89.89 | 18.46 |
| *Melanthera nivea* | -89.89 | 18.38 |
| *Melanthera nivea* | -89.89 | 18.53 |
| *Melanthera nivea* | -89.89 | 18.36 |
| *Melanthera nivea* | -89.88 | 17.07 |
| *Melanthera nivea* | -89.88 | 20.66 |
| *Melanthera nivea* | -89.87 | 20.65 |
| *Melanthera nivea* | -89.87 | 20.65 |
| *Melanthera nivea* | -89.85 | 18.61 |
| *Melanthera nivea* | -89.85 | 18.61 |
| *Melanthera nivea* | -89.84 | 21.25 |
| *Melanthera nivea* | -89.83 | 21.25 |
| *Melanthera nivea* | -89.83 | 18.26 |
| *Melanthera nivea* | -89.83 | 21.25 |
| *Melanthera nivea* | -89.82 | 18.12 |
| *Melanthera nivea* | -89.82 | 18.26 |
| *Melanthera nivea* | -89.82 | 18.12 |
| *Melanthera nivea* | -89.82 | 18.12 |
| *Melanthera nivea* | -89.82 | 18.12 |
| *Melanthera nivea* | -89.82 | 18.12 |
| *Melanthera nivea* | -89.82 | 18.12 |
| *Melanthera nivea* | -89.81 | 18.12 |
| *Melanthera nivea* | -89.80 | 18.14 |
| *Melanthera nivea* | -89.80 | 21.16 |
| *Melanthera nivea* | -89.80 | 13.90 |
| *Melanthera nivea* | -89.80 | 13.87 |
| *Melanthera nivea* | -89.79 | 20.66 |
| *Melanthera nivea* | -89.78 | 20.65 |
| *Melanthera nivea* | -89.72 | 14.12 |
| *Melanthera nivea* | -89.72 | 20.87 |
| *Melanthera nivea* | -89.72 | 20.87 |
| *Melanthera nivea* | -89.72 | 20.52 |
| *Melanthera nivea* | -89.72 | 15.55 |
| *Melanthera nivea* | -89.72 | 17.00 |
| *Melanthera nivea* | -89.72 | 17.00 |
| *Melanthera nivea* | -89.69 | 21.06 |
| *Melanthera nivea* | -89.69 | 16.99 |
| *Melanthera nivea* | -89.66 | 21.28 |
| *Melanthera nivea* | -89.66 | 21.28 |
| *Melanthera nivea* | -89.66 | 19.50 |
| *Melanthera nivea* | -89.66 | 21.29 |
| *Melanthera nivea* | -89.66 | 21.29 |
| *Melanthera nivea* | -89.65 | 21.17 |
| *Melanthera nivea* | -89.65 | 21.18 |
| *Melanthera nivea* | -89.64 | 21.03 |
| *Melanthera nivea* | -89.64 | 21.26 |
| *Melanthera nivea* | -89.64 | 21.03 |
| *Melanthera nivea* | -89.64 | 21.01 |
| *Melanthera nivea* | -89.64 | 21.03 |
| *Melanthera nivea* | -89.64 | 21.10 |
| *Melanthera nivea* | -89.63 | 20.99 |
| *Melanthera nivea* | -89.63 | 20.98 |
| *Melanthera nivea* | -89.62 | 20.98 |
| *Melanthera nivea* | -89.62 | 20.98 |
| *Melanthera nivea* | -89.62 | 20.98 |
| *Melanthera nivea* | -89.61 | 18.53 |
| *Melanthera nivea* | -89.61 | 21.03 |
| *Melanthera nivea* | -89.61 | 21.03 |
| *Melanthera nivea* | -89.60 | 13.68 |
| *Melanthera nivea* | -89.60 | 21.09 |
| *Melanthera nivea* | -89.59 | 21.00 |
| *Melanthera nivea* | -89.58 | 18.53 |
| *Melanthera nivea* | -89.58 | 18.53 |
| *Melanthera nivea* | -89.56 | 20.84 |
| *Melanthera nivea* | -89.56 | 20.84 |
| *Melanthera nivea* | -89.56 | 20.84 |
| *Melanthera nivea* | -89.56 | 21.04 |
| *Melanthera nivea* | -89.53 | 20.40 |
| *Melanthera nivea* | -89.53 | 20.40 |
| *Melanthera nivea* | -89.53 | 21.12 |
| *Melanthera nivea* | -89.53 | 17.69 |
| *Melanthera nivea* | -89.53 | 17.69 |
| *Melanthera nivea* | -89.53 | 21.06 |
| *Melanthera nivea* | -89.53 | 21.08 |
| *Melanthera nivea* | -89.52 | 21.10 |
| *Melanthera nivea* | -89.52 | 21.30 |
| *Melanthera nivea* | -89.52 | 21.14 |
| *Melanthera nivea* | -89.51 | 20.27 |
| *Melanthera nivea* | -89.51 | 21.31 |
| *Melanthera nivea* | -89.49 | 21.31 |
| *Melanthera nivea* | -89.48 | 18.23 |
| *Melanthera nivea* | -89.48 | 21.15 |
| *Melanthera nivea* | -89.48 | 18.23 |
| *Melanthera nivea* | -89.47 | 18.23 |
| *Melanthera nivea* | -89.47 | 14.52 |
| *Melanthera nivea* | -89.46 | 18.25 |
| *Melanthera nivea* | -89.46 | 18.10 |
| *Melanthera nivea* | -89.46 | 18.09 |
| *Melanthera nivea* | -89.46 | 18.24 |
| *Melanthera nivea* | -89.45 | 21.11 |
| *Melanthera nivea* | -89.45 | 18.23 |
| *Melanthera nivea* | -89.45 | 18.23 |
| *Melanthera nivea* | -89.45 | 21.32 |
| *Melanthera nivea* | -89.45 | 20.26 |
| *Melanthera nivea* | -89.45 | 18.24 |
| *Melanthera nivea* | -89.45 | 20.25 |
| *Melanthera nivea* | -89.45 | 18.25 |
| *Melanthera nivea* | -89.45 | 20.25 |
| *Melanthera nivea* | -89.45 | 20.26 |
| *Melanthera nivea* | -89.45 | 20.25 |
| *Melanthera nivea* | -89.45 | 20.26 |
| *Melanthera nivea* | -89.45 | 18.24 |
| *Melanthera nivea* | -89.45 | 18.22 |
| *Melanthera nivea* | -89.45 | 20.25 |
| *Melanthera nivea* | -89.45 | 21.32 |
| *Melanthera nivea* | -89.44 | 20.98 |
| *Melanthera nivea* | -89.44 | 18.68 |
| *Melanthera nivea* | -89.44 | 20.99 |
| *Melanthera nivea* | -89.44 | 20.98 |
| *Melanthera nivea* | -89.44 | 18.42 |
| *Melanthera nivea* | -89.44 | 18.43 |
| *Melanthera nivea* | -89.43 | 18.43 |
| *Melanthera nivea* | -89.43 | 20.27 |
| *Melanthera nivea* | -89.43 | 18.67 |
| *Melanthera nivea* | -89.43 | 20.26 |
| *Melanthera nivea* | -89.43 | 20.89 |
| *Melanthera nivea* | -89.43 | 18.59 |
| *Melanthera nivea* | -89.43 | 20.89 |
| *Melanthera nivea* | -89.42 | 20.89 |
| *Melanthera nivea* | -89.42 | 20.89 |
| *Melanthera nivea* | -89.42 | 20.97 |
| *Melanthera nivea* | -89.42 | 18.58 |
| *Melanthera nivea* | -89.41 | 18.59 |
| *Melanthera nivea* | -89.41 | 18.59 |
| *Melanthera nivea* | -89.41 | 18.59 |
| *Melanthera nivea* | -89.41 | 18.61 |
| *Melanthera nivea* | -89.41 | 18.00 |
| *Melanthera nivea* | -89.41 | 18.65 |
| *Melanthera nivea* | -89.41 | 18.63 |
| *Melanthera nivea* | -89.41 | 18.66 |
| *Melanthera nivea* | -89.41 | 18.00 |
| *Melanthera nivea* | -89.41 | 18.65 |
| *Melanthera nivea* | -89.41 | 18.66 |
| *Melanthera nivea* | -89.40 | 18.00 |
| *Melanthera nivea* | -89.40 | 18.66 |
| *Melanthera nivea* | -89.40 | 18.73 |
| *Melanthera nivea* | -89.40 | 18.68 |
| *Melanthera nivea* | -89.39 | 20.39 |
| *Melanthera nivea* | -89.39 | 19.27 |
| *Melanthera nivea* | -89.39 | 17.06 |
| *Melanthera nivea* | -89.39 | 19.27 |
| *Melanthera nivea* | -89.38 | 18.43 |
| *Melanthera nivea* | -89.38 | 18.43 |
| *Melanthera nivea* | -89.38 | 13.66 |
| *Melanthera nivea* | -89.38 | 18.79 |
| *Melanthera nivea* | -89.37 | 13.63 |
| *Melanthera nivea* | -89.37 | 18.85 |
| *Melanthera nivea* | -89.37 | 15.13 |
| *Melanthera nivea* | -89.36 | 18.86 |
| *Melanthera nivea* | -89.36 | 18.86 |
| *Melanthera nivea* | -89.36 | 17.87 |
| *Melanthera nivea* | -89.35 | 18.68 |
| *Melanthera nivea* | -89.35 | 18.68 |
| *Melanthera nivea* | -89.35 | 18.88 |
| *Melanthera nivea* | -89.35 | 18.67 |
| *Melanthera nivea* | -89.35 | 18.88 |
| *Melanthera nivea* | -89.35 | 13.78 |
| *Melanthera nivea* | -89.35 | 20.26 |
| *Melanthera nivea* | -89.33 | 15.56 |
| *Melanthera nivea* | -89.33 | 14.56 |
| *Melanthera nivea* | -89.32 | 17.91 |
| *Melanthera nivea* | -89.32 | 17.91 |
| *Melanthera nivea* | -89.31 | 14.56 |
| *Melanthera nivea* | -89.31 | 18.97 |
| *Melanthera nivea* | -89.30 | 18.93 |
| *Melanthera nivea* | -89.30 | 13.48 |
| *Melanthera nivea* | -89.30 | 13.80 |
| *Melanthera nivea* | -89.30 | 21.29 |
| *Melanthera nivea* | -89.30 | 21.29 |
| *Melanthera nivea* | -89.28 | 13.68 |
| *Melanthera nivea* | -89.27 | 21.33 |
| *Melanthera nivea* | -89.26 | 18.49 |
| *Melanthera nivea* | -89.26 | 18.49 |
| *Melanthera nivea* | -89.26 | 20.19 |
| *Melanthera nivea* | -89.25 | 13.67 |
| *Melanthera nivea* | -89.25 | 13.68 |
| *Melanthera nivea* | -89.25 | 13.67 |
| *Melanthera nivea* | -89.25 | 21.33 |
| *Melanthera nivea* | -89.24 | 18.01 |
| *Melanthera nivea* | -89.24 | 21.34 |
| *Melanthera nivea* | -89.24 | 19.19 |
| *Melanthera nivea* | -89.24 | 19.19 |
| *Melanthera nivea* | -89.24 | 19.19 |
| *Melanthera nivea* | -89.24 | 19.18 |
| *Melanthera nivea* | -89.23 | 21.34 |
| *Melanthera nivea* | -89.22 | 19.24 |
| *Melanthera nivea* | -89.22 | 19.24 |
| *Melanthera nivea* | -89.22 | 19.23 |
| *Melanthera nivea* | -89.20 | 13.71 |
| *Melanthera nivea* | -89.20 | 21.35 |
| *Melanthera nivea* | -89.18 | 13.66 |
| *Melanthera nivea* | -89.18 | 17.92 |
| *Melanthera nivea* | -89.18 | 17.06 |
| *Melanthera nivea* | -89.18 | 19.59 |
| *Melanthera nivea* | -89.18 | 19.59 |
| *Melanthera nivea* | -89.17 | 19.20 |
| *Melanthera nivea* | -89.17 | 19.20 |
| *Melanthera nivea* | -89.17 | 17.88 |
| *Melanthera nivea* | -89.17 | 19.58 |
| *Melanthera nivea* | -89.17 | 19.19 |
| *Melanthera nivea* | -89.17 | 19.19 |
| *Melanthera nivea* | -89.17 | 19.19 |
| *Melanthera nivea* | -89.17 | 21.35 |
| *Melanthera nivea* | -89.17 | 21.35 |
| *Melanthera nivea* | -89.16 | 21.35 |
| *Melanthera nivea* | -89.15 | 19.72 |
| *Melanthera nivea* | -89.15 | 19.72 |
| *Melanthera nivea* | -89.15 | 19.72 |
| *Melanthera nivea* | -89.14 | 18.57 |
| *Melanthera nivea* | -89.13 | 20.93 |
| *Melanthera nivea* | -89.12 | 18.12 |
| *Melanthera nivea* | -89.12 | 18.30 |
| *Melanthera nivea* | -89.11 | 17.17 |
| *Melanthera nivea* | -89.11 | 19.69 |
| *Melanthera nivea* | -89.11 | 19.69 |
| *Melanthera nivea* | -89.11 | 19.69 |
| *Melanthera nivea* | -89.11 | 19.69 |
| *Melanthera nivea* | -89.11 | 19.69 |
| *Melanthera nivea* | -89.11 | 20.16 |
| *Melanthera nivea* | -89.10 | 20.16 |
| *Melanthera nivea* | -89.10 | 17.08 |
| *Melanthera nivea* | -89.10 | 20.15 |
| *Melanthera nivea* | -89.08 | 14.08 |
| *Melanthera nivea* | -89.08 | 17.10 |
| *Melanthera nivea* | -89.07 | 17.10 |
| *Melanthera nivea* | -89.07 | 16.25 |
| *Melanthera nivea* | -89.07 | 17.10 |
| *Melanthera nivea* | -89.05 | 16.28 |
| *Melanthera nivea* | -89.05 | 16.28 |
| *Melanthera nivea* | -89.05 | 20.09 |
| *Melanthera nivea* | -89.04 | 15.27 |
| *Melanthera nivea* | -89.03 | 16.67 |
| *Melanthera nivea* | -89.03 | 16.87 |
| *Melanthera nivea* | -89.03 | 18.30 |
| *Melanthera nivea* | -89.03 | 16.67 |
| *Melanthera nivea* | -89.02 | 16.28 |
| *Melanthera nivea* | -89.02 | 19.69 |
| *Melanthera nivea* | -89.02 | 19.69 |
| *Melanthera nivea* | -89.02 | 20.93 |
| *Melanthera nivea* | -89.02 | 20.93 |
| *Melanthera nivea* | -89.02 | 19.69 |
| *Melanthera nivea* | -89.02 | 21.37 |
| *Melanthera nivea* | -89.01 | 18.37 |
| *Melanthera nivea* | -89.01 | 21.36 |
| *Melanthera nivea* | -89.01 | 19.68 |
| *Melanthera nivea* | -89.01 | 19.42 |
| *Melanthera nivea* | -89.01 | 19.42 |
| *Melanthera nivea* | -89.01 | 20.03 |
| *Melanthera nivea* | -89.00 | 16.92 |
| *Melanthera nivea* | -89.00 | 13.67 |
| *Melanthera nivea* | -89.00 | 19.41 |
| *Melanthera nivea* | -88.99 | 17.08 |
| *Melanthera nivea* | -88.99 | 18.19 |
| *Melanthera nivea* | -88.98 | 16.97 |
| *Melanthera nivea* | -88.98 | 16.72 |
| *Melanthera nivea* | -88.98 | 16.97 |
| *Melanthera nivea* | -88.98 | 18.19 |
| *Melanthera nivea* | -88.98 | 18.19 |
| *Melanthera nivea* | -88.98 | 18.19 |
| *Melanthera nivea* | -88.97 | 20.63 |
| *Melanthera nivea* | -88.97 | 20.63 |
| *Melanthera nivea* | -88.97 | 17.05 |
| *Melanthera nivea* | -88.97 | 17.05 |
| *Melanthera nivea* | -88.97 | 17.02 |
| *Melanthera nivea* | -88.97 | 17.05 |
| *Melanthera nivea* | -88.94 | 20.52 |
| *Melanthera nivea* | -88.93 | 16.78 |
| *Melanthera nivea* | -88.93 | 16.22 |
| *Melanthera nivea* | -88.93 | 16.82 |
| *Melanthera nivea* | -88.93 | 20.50 |
| *Melanthera nivea* | -88.92 | 16.83 |
| *Melanthera nivea* | -88.92 | 20.49 |
| *Melanthera nivea* | -88.92 | 17.05 |
| *Melanthera nivea* | -88.92 | 16.85 |
| *Melanthera nivea* | -88.92 | 20.49 |
| *Melanthera nivea* | -88.91 | 20.46 |
| *Melanthera nivea* | -88.90 | 20.49 |
| *Melanthera nivea* | -88.90 | 20.97 |
| *Melanthera nivea* | -88.90 | 20.97 |
| *Melanthera nivea* | -88.90 | 20.45 |
| *Melanthera nivea* | -88.89 | 19.52 |
| *Melanthera nivea* | -88.89 | 19.52 |
| *Melanthera nivea* | -88.88 | 16.88 |
| *Melanthera nivea* | -88.88 | 17.92 |
| *Melanthera nivea* | -88.87 | 19.54 |
| *Melanthera nivea* | -88.87 | 19.54 |
| *Melanthera nivea* | -88.87 | 19.53 |
| *Melanthera nivea* | -88.87 | 13.74 |
| *Melanthera nivea* | -88.86 | 20.55 |
| *Melanthera nivea* | -88.84 | 20.56 |
| *Melanthera nivea* | -88.83 | 18.73 |
| *Melanthera nivea* | -88.83 | 20.91 |
| *Melanthera nivea* | -88.83 | 20.58 |
| *Melanthera nivea* | -88.83 | 19.32 |
| *Melanthera nivea* | -88.80 | 18.48 |
| *Melanthera nivea* | -88.80 | 19.79 |
| *Melanthera nivea* | -88.80 | 19.94 |
| *Melanthera nivea* | -88.79 | 17.26 |
| *Melanthera nivea* | -88.78 | 17.23 |
| *Melanthera nivea* | -88.78 | 18.65 |
| *Melanthera nivea* | -88.78 | 17.22 |
| *Melanthera nivea* | -88.78 | 17.22 |
| *Melanthera nivea* | -88.77 | 17.17 |
| *Melanthera nivea* | -88.77 | 16.52 |
| *Melanthera nivea* | -88.77 | 17.17 |
| *Melanthera nivea* | -88.76 | 20.90 |
| *Melanthera nivea* | -88.76 | 19.90 |
| *Melanthera nivea* | -88.71 | 17.15 |
| *Melanthera nivea* | -88.71 | 20.50 |
| *Melanthera nivea* | -88.71 | 18.48 |
| *Melanthera nivea* | -88.70 | 17.58 |
| *Melanthera nivea* | -88.69 | 19.30 |
| *Melanthera nivea* | -88.68 | 17.22 |
| *Melanthera nivea* | -88.68 | 19.30 |
| *Melanthera nivea* | -88.68 | 17.60 |
| *Melanthera nivea* | -88.68 | 14.45 |
| *Melanthera nivea* | -88.68 | 17.15 |
| *Melanthera nivea* | -88.68 | 19.50 |
| *Melanthera nivea* | -88.68 | 19.50 |
| *Melanthera nivea* | -88.66 | 17.28 |
| *Melanthera nivea* | -88.66 | 18.24 |
| *Melanthera nivea* | -88.65 | 17.27 |
| *Melanthera nivea* | -88.64 | 18.49 |
| *Melanthera nivea* | -88.63 | 14.57 |
| *Melanthera nivea* | -88.63 | 18.49 |
| *Melanthera nivea* | -88.63 | 18.49 |
| *Melanthera nivea* | -88.62 | 18.49 |
| *Melanthera nivea* | -88.62 | 17.28 |
| *Melanthera nivea* | -88.60 | 21.27 |
| *Melanthera nivea* | -88.59 | 19.51 |
| *Melanthera nivea* | -88.59 | 19.59 |
| *Melanthera nivea* | -88.58 | 14.58 |
| *Melanthera nivea* | -88.57 | 20.67 |
| *Melanthera nivea* | -88.57 | 20.68 |
| *Melanthera nivea* | -88.57 | 20.68 |
| *Melanthera nivea* | -88.57 | 20.69 |
| *Melanthera nivea* | -88.57 | 18.17 |
| *Melanthera nivea* | -88.56 | 17.34 |
| *Melanthera nivea* | -88.56 | 19.38 |
| *Melanthera nivea* | -88.56 | 18.78 |
| *Melanthera nivea* | -88.56 | 19.39 |
| *Melanthera nivea* | -88.55 | 17.35 |
| *Melanthera nivea* | -88.55 | 18.39 |
| *Melanthera nivea* | -88.55 | 18.02 |
| *Melanthera nivea* | -88.55 | 17.37 |
| *Melanthera nivea* | -88.54 | 18.24 |
| *Melanthera nivea* | -88.53 | 13.48 |
| *Melanthera nivea* | -88.53 | 17.35 |
| *Melanthera nivea* | -88.53 | 17.22 |
| *Melanthera nivea* | -88.51 | 21.49 |
| *Melanthera nivea* | -88.51 | 20.57 |
| *Melanthera nivea* | -88.50 | 18.95 |
| *Melanthera nivea* | -88.50 | 19.15 |
| *Melanthera nivea* | -88.49 | 19.14 |
| *Melanthera nivea* | -88.46 | 19.08 |
| *Melanthera nivea* | -88.46 | 19.08 |
| *Melanthera nivea* | -88.46 | 18.57 |
| *Melanthera nivea* | -88.45 | 19.08 |
| *Melanthera nivea* | -88.45 | 17.40 |
| *Melanthera nivea* | -88.45 | 19.08 |
| *Melanthera nivea* | -88.44 | 17.42 |
| *Melanthera nivea* | -88.43 | 17.40 |
| *Melanthera nivea* | -88.43 | 20.37 |
| *Melanthera nivea* | -88.41 | 18.77 |
| *Melanthera nivea* | -88.41 | 18.50 |
| *Melanthera nivea* | -88.40 | 18.20 |
| *Melanthera nivea* | -88.38 | 17.46 |
| *Melanthera nivea* | -88.38 | 18.39 |
| *Melanthera nivea* | -88.38 | 18.39 |
| *Melanthera nivea* | -88.38 | 18.51 |
| *Melanthera nivea* | -88.35 | 17.55 |
| *Melanthera nivea* | -88.34 | 18.52 |
| *Melanthera nivea* | -88.33 | 16.98 |
| *Melanthera nivea* | -88.33 | 18.52 |
| *Melanthera nivea* | -88.31 | 17.56 |
| *Melanthera nivea* | -88.31 | 18.51 |
| *Melanthera nivea* | -88.31 | 18.50 |
| *Melanthera nivea* | -88.31 | 18.50 |
| *Melanthera nivea* | -88.31 | 18.50 |
| *Melanthera nivea* | -88.30 | 18.50 |
| *Melanthera nivea* | -88.30 | 18.51 |
| *Melanthera nivea* | -88.30 | 18.51 |
| *Melanthera nivea* | -88.29 | 20.72 |
| *Melanthera nivea* | -88.29 | 17.65 |
| *Melanthera nivea* | -88.28 | 20.73 |
| *Melanthera nivea* | -88.27 | 20.72 |
| *Melanthera nivea* | -88.27 | 18.69 |
| *Melanthera nivea* | -88.27 | 19.97 |
| *Melanthera nivea* | -88.27 | 20.71 |
| *Melanthera nivea* | -88.26 | 20.71 |
| *Melanthera nivea* | -88.26 | 18.98 |
| *Melanthera nivea* | -88.26 | 20.66 |
| *Melanthera nivea* | -88.25 | 20.65 |
| *Melanthera nivea* | -88.25 | 20.71 |
| *Melanthera nivea* | -88.24 | 20.66 |
| *Melanthera nivea* | -88.24 | 20.68 |
| *Melanthera nivea* | -88.24 | 20.71 |
| *Melanthera nivea* | -88.24 | 20.70 |
| *Melanthera nivea* | -88.23 | 20.70 |
| *Melanthera nivea* | -88.23 | 20.70 |
| *Melanthera nivea* | -88.22 | 16.97 |
| *Melanthera nivea* | -88.22 | 13.77 |
| *Melanthera nivea* | -88.21 | 20.63 |
| *Melanthera nivea* | -88.21 | 20.71 |
| *Melanthera nivea* | -88.20 | 20.62 |
| *Melanthera nivea* | -88.20 | 20.70 |
| *Melanthera nivea* | -88.20 | 20.62 |
| *Melanthera nivea* | -88.18 | 13.88 |
| *Melanthera nivea* | -88.18 | 21.59 |
| *Melanthera nivea* | -88.18 | 21.53 |
| *Melanthera nivea* | -88.16 | 19.78 |
| *Melanthera nivea* | -88.16 | 19.62 |
| *Melanthera nivea* | -88.16 | 20.68 |
| *Melanthera nivea* | -88.16 | 20.68 |
| *Melanthera nivea* | -88.15 | 20.64 |
| *Melanthera nivea* | -88.13 | 19.46 |
| *Melanthera nivea* | -88.11 | 19.40 |
| *Melanthera nivea* | -88.11 | 19.07 |
| *Melanthera nivea* | -88.11 | 19.07 |
| *Melanthera nivea* | -88.11 | 19.28 |
| *Melanthera nivea* | -88.11 | 21.58 |
| *Melanthera nivea* | -88.11 | 19.09 |
| *Melanthera nivea* | -88.10 | 19.23 |
| *Melanthera nivea* | -88.10 | 21.57 |
| *Melanthera nivea* | -88.10 | 19.26 |
| *Melanthera nivea* | -88.10 | 19.26 |
| *Melanthera nivea* | -88.10 | 19.26 |
| *Melanthera nivea* | -88.08 | 19.86 |
| *Melanthera nivea* | -88.07 | 15.22 |
| *Melanthera nivea* | -88.07 | 13.60 |
| *Melanthera nivea* | -88.07 | 19.85 |
| *Melanthera nivea* | -88.07 | 21.37 |
| *Melanthera nivea* | -88.06 | 21.37 |
| *Melanthera nivea* | -88.06 | 21.01 |
| *Melanthera nivea* | -88.05 | 14.99 |
| *Melanthera nivea* | -88.05 | 20.77 |
| *Melanthera nivea* | -88.03 | 19.58 |
| *Melanthera nivea* | -88.03 | 20.42 |
| *Melanthera nivea* | -88.03 | 20.42 |
| *Melanthera nivea* | -88.01 | 19.88 |
| *Melanthera nivea* | -88.01 | 21.54 |
| *Melanthera nivea* | -87.99 | 21.54 |
| *Melanthera nivea* | -87.99 | 21.61 |
| *Melanthera nivea* | -87.99 | 19.35 |
| *Melanthera nivea* | -87.98 | 19.48 |
| *Melanthera nivea* | -87.97 | 19.49 |
| *Melanthera nivea* | -87.97 | 19.62 |
| *Melanthera nivea* | -87.96 | 19.62 |
| *Melanthera nivea* | -87.95 | 19.62 |
| *Melanthera nivea* | -87.92 | 17.92 |
| *Melanthera nivea* | -87.92 | 19.64 |
| *Melanthera nivea* | -87.90 | 21.57 |
| *Melanthera nivea* | -87.88 | 19.94 |
| *Melanthera nivea* | -87.86 | 18.31 |
| *Melanthera nivea* | -87.85 | 14.78 |
| *Melanthera nivea* | -87.85 | 13.33 |
| *Melanthera nivea* | -87.85 | 18.30 |
| *Melanthera nivea* | -87.85 | 19.88 |
| *Melanthera nivea* | -87.84 | 21.56 |
| *Melanthera nivea* | -87.83 | 18.29 |
| *Melanthera nivea* | -87.83 | 13.27 |
| *Melanthera nivea* | -87.83 | 18.31 |
| *Melanthera nivea* | -87.79 | 18.44 |
| *Melanthera nivea* | -87.78 | 14.95 |
| *Melanthera nivea* | -87.78 | 18.44 |
| *Melanthera nivea* | -87.77 | 19.92 |
| *Melanthera nivea* | -87.77 | 18.43 |
| *Melanthera nivea* | -87.76 | 18.50 |
| *Melanthera nivea* | -87.75 | 13.30 |
| *Melanthera nivea* | -87.74 | 20.50 |
| *Melanthera nivea* | -87.74 | 20.49 |
| *Melanthera nivea* | -87.73 | 20.50 |
| *Melanthera nivea* | -87.73 | 21.38 |
| *Melanthera nivea* | -87.73 | 20.49 |
| *Melanthera nivea* | -87.73 | 20.49 |
| *Melanthera nivea* | -87.72 | 20.51 |
| *Melanthera nivea* | -87.72 | 18.75 |
| *Melanthera nivea* | -87.71 | 14.87 |
| *Melanthera nivea* | -87.71 | 18.72 |
| *Melanthera nivea* | -87.71 | 14.26 |
| *Melanthera nivea* | -87.71 | 18.72 |
| *Melanthera nivea* | -87.70 | 20.50 |
| *Melanthera nivea* | -87.69 | 14.44 |
| *Melanthera nivea* | -87.67 | 21.52 |
| *Melanthera nivea* | -87.65 | 20.62 |
| *Melanthera nivea* | -87.65 | 19.78 |
| *Melanthera nivea* | -87.65 | 14.77 |
| *Melanthera nivea* | -87.64 | 13.27 |
| *Melanthera nivea* | -87.64 | 19.79 |
| *Melanthera nivea* | -87.64 | 19.79 |
| *Melanthera nivea* | -87.63 | 19.79 |
| *Melanthera nivea* | -87.63 | 19.79 |
| *Melanthera nivea* | -87.62 | 19.79 |
| *Melanthera nivea* | -87.62 | 18.95 |
| *Melanthera nivea* | -87.62 | 18.95 |
| *Melanthera nivea* | -87.61 | 19.79 |
| *Melanthera nivea* | -87.60 | 19.78 |
| *Melanthera nivea* | -87.60 | 19.78 |
| *Melanthera nivea* | -87.60 | 19.78 |
| *Melanthera nivea* | -87.58 | 15.04 |
| *Melanthera nivea* | -87.50 | 15.79 |
| *Melanthera nivea* | -87.50 | 20.25 |
| *Melanthera nivea* | -87.48 | 20.03 |
| *Melanthera nivea* | -87.48 | 20.03 |
| *Melanthera nivea* | -87.48 | 20.03 |
| *Melanthera nivea* | -87.46 | 20.21 |
| *Melanthera nivea* | -87.45 | 15.73 |
| *Melanthera nivea* | -87.45 | 15.73 |
| *Melanthera nivea* | -87.44 | 20.20 |
| *Melanthera nivea* | -87.44 | 20.20 |
| *Melanthera nivea* | -87.43 | 20.23 |
| *Melanthera nivea* | -87.43 | 20.22 |
| *Melanthera nivea* | -87.37 | 14.94 |
| *Melanthera nivea* | -87.35 | 20.33 |
| *Melanthera nivea* | -87.21 | 14.12 |
| *Melanthera nivea* | -87.19 | 14.09 |
| *Melanthera nivea* | -87.18 | 21.21 |
| *Melanthera nivea* | -87.17 | 14.09 |
| *Melanthera nivea* | -87.17 | 14.09 |
| *Melanthera nivea* | -87.13 | 20.57 |
| *Melanthera nivea* | -87.11 | 14.11 |
| *Melanthera nivea* | -87.08 | 14.12 |
| *Melanthera nivea* | -87.05 | 12.70 |
| *Melanthera nivea* | -87.05 | 12.38 |
| *Melanthera nivea* | -87.05 | 14.16 |
| *Melanthera nivea* | -87.02 | 20.40 |
| *Melanthera nivea* | -87.02 | 12.38 |
| *Melanthera nivea* | -87.00 | 12.70 |
| *Melanthera nivea* | -87.00 | 20.37 |
| *Melanthera nivea* | -86.99 | 20.29 |
| *Melanthera nivea* | -86.99 | 13.96 |
| *Melanthera nivea* | -86.99 | 20.51 |
| *Melanthera nivea* | -86.98 | 20.74 |
| *Melanthera nivea* | -86.97 | 20.76 |
| *Melanthera nivea* | -86.97 | 20.76 |
| *Melanthera nivea* | -86.95 | 20.50 |
| *Melanthera nivea* | -86.95 | 20.48 |
| *Melanthera nivea* | -86.94 | 20.52 |
| *Melanthera nivea* | -86.93 | 20.52 |
| *Melanthera nivea* | -86.92 | 20.82 |
| *Melanthera nivea* | -86.91 | 15.71 |
| *Melanthera nivea* | -86.91 | 20.82 |
| *Melanthera nivea* | -86.88 | 20.95 |
| *Melanthera nivea* | -86.88 | 20.95 |
| *Melanthera nivea* | -86.88 | 20.95 |
| *Melanthera nivea* | -86.88 | 20.85 |
| *Melanthera nivea* | -86.88 | 20.85 |
| *Melanthera nivea* | -86.88 | 20.95 |
| *Melanthera nivea* | -86.88 | 20.85 |
| *Melanthera nivea* | -86.88 | 20.85 |
| *Melanthera nivea* | -86.87 | 20.84 |
| *Melanthera nivea* | -86.87 | 12.42 |
| *Melanthera nivea* | -86.85 | 21.07 |
| *Melanthera nivea* | -86.85 | 21.07 |
| *Melanthera nivea* | -86.83 | 20.97 |
| *Melanthera nivea* | -86.83 | 20.99 |
| *Melanthera nivea* | -86.83 | 21.13 |
| *Melanthera nivea* | -86.82 | 21.20 |
| *Melanthera nivea* | -86.81 | 21.18 |
| *Melanthera nivea* | -86.79 | 21.49 |
| *Melanthera nivea* | -86.79 | 21.04 |
| *Melanthera nivea* | -86.77 | 15.73 |
| *Melanthera nivea* | -86.74 | 21.24 |
| *Melanthera nivea* | -86.73 | 21.25 |
| *Melanthera nivea* | -86.73 | 21.23 |
| *Melanthera nivea* | -86.73 | 21.23 |
| *Melanthera nivea* | -86.73 | 21.22 |
| *Melanthera nivea* | -86.72 | 21.22 |
| *Melanthera nivea* | -86.66 | 14.56 |
| *Melanthera nivea* | -86.63 | 13.32 |
| *Melanthera nivea* | -86.58 | 12.42 |
| *Melanthera nivea* | -86.58 | 13.43 |
| *Melanthera nivea* | -86.58 | 13.42 |
| *Melanthera nivea* | -86.55 | 13.42 |
| *Melanthera nivea* | -86.53 | 12.38 |
| *Melanthera nivea* | -86.53 | 13.48 |
| *Melanthera nivea* | -86.52 | 12.40 |
| *Melanthera nivea* | -86.52 | 11.78 |
| *Melanthera nivea* | -86.52 | 13.50 |
| *Melanthera nivea* | -86.50 | 11.87 |
| *Melanthera nivea* | -86.50 | 11.88 |
| *Melanthera nivea* | -86.43 | 12.10 |
| *Melanthera nivea* | -86.43 | 13.12 |
| *Melanthera nivea* | -86.42 | 13.42 |
| *Melanthera nivea* | -86.42 | 12.70 |
| *Melanthera nivea* | -86.42 | 13.10 |
| *Melanthera nivea* | -86.40 | 12.98 |
| *Melanthera nivea* | -86.38 | 13.22 |
| *Melanthera nivea* | -86.37 | 12.29 |
| *Melanthera nivea* | -86.37 | 13.25 |
| *Melanthera nivea* | -86.37 | 12.98 |
| *Melanthera nivea* | -86.36 | 13.71 |
| *Melanthera nivea* | -86.35 | 11.95 |
| *Melanthera nivea* | -86.35 | 13.25 |
| *Melanthera nivea* | -86.35 | 13.27 |
| *Melanthera nivea* | -86.35 | 13.03 |
| *Melanthera nivea* | -86.33 | 13.23 |
| *Melanthera nivea* | -86.33 | 11.95 |
| *Melanthera nivea* | -86.32 | 11.97 |
| *Melanthera nivea* | -86.32 | 13.20 |
| *Melanthera nivea* | -86.32 | 13.15 |
| *Melanthera nivea* | -86.32 | 12.08 |
| *Melanthera nivea* | -86.32 | 11.98 |
| *Melanthera nivea* | -86.32 | 13.70 |
| *Melanthera nivea* | -86.30 | 12.15 |
| *Melanthera nivea* | -86.30 | 13.03 |
| *Melanthera nivea* | -86.30 | 13.18 |
| *Melanthera nivea* | -86.30 | 13.17 |
| *Melanthera nivea* | -86.30 | 11.97 |
| *Melanthera nivea* | -86.28 | 12.02 |
| *Melanthera nivea* | -86.27 | 13.02 |
| *Melanthera nivea* | -86.27 | 13.27 |
| *Melanthera nivea* | -86.27 | 12.15 |
| *Melanthera nivea* | -86.26 | 13.39 |
| *Melanthera nivea* | -86.25 | 13.23 |
| *Melanthera nivea* | -86.25 | 13.00 |
| *Melanthera nivea* | -86.25 | 12.10 |
| *Melanthera nivea* | -86.24 | 13.42 |
| *Melanthera nivea* | -86.24 | 13.30 |
| *Melanthera nivea* | -86.23 | 13.22 |
| *Melanthera nivea* | -86.23 | 12.07 |
| *Melanthera nivea* | -86.21 | 11.55 |
| *Melanthera nivea* | -86.20 | 11.82 |
| *Melanthera nivea* | -86.17 | 11.78 |
| *Melanthera nivea* | -86.15 | 12.00 |
| *Melanthera nivea* | -86.15 | 12.07 |
| *Melanthera nivea* | -86.13 | 12.15 |
| *Melanthera nivea* | -86.12 | 15.00 |
| *Melanthera nivea* | -86.12 | 12.13 |
| *Melanthera nivea* | -86.12 | 12.38 |
| *Melanthera nivea* | -86.12 | 12.17 |
| *Melanthera nivea* | -86.10 | 12.85 |
| *Melanthera nivea* | -86.10 | 13.75 |
| *Melanthera nivea* | -86.10 | 13.12 |
| *Melanthera nivea* | -86.08 | 13.77 |
| *Melanthera nivea* | -86.08 | 12.38 |
| *Melanthera nivea* | -86.08 | 13.72 |
| *Melanthera nivea* | -86.07 | 11.92 |
| *Melanthera nivea* | -86.07 | 12.58 |
| *Melanthera nivea* | -86.07 | 11.92 |
| *Melanthera nivea* | -86.05 | 12.62 |
| *Melanthera nivea* | -86.05 | 11.88 |
| *Melanthera nivea* | -86.05 | 12.35 |
| *Melanthera nivea* | -86.05 | 11.90 |
| *Melanthera nivea* | -86.03 | 11.95 |
| *Melanthera nivea* | -86.00 | 11.83 |
| *Melanthera nivea* | -86.00 | 11.85 |
| *Melanthera nivea* | -86.00 | 13.72 |
| *Melanthera nivea* | -86.00 | 13.13 |
| *Melanthera nivea* | -85.98 | 11.75 |
| *Melanthera nivea* | -85.98 | 11.85 |
| *Melanthera nivea* | -85.98 | 13.07 |
| *Melanthera nivea* | -85.98 | 11.34 |
| *Melanthera nivea* | -85.97 | 11.87 |
| *Melanthera nivea* | -85.97 | 11.82 |
| *Melanthera nivea* | -85.97 | 11.82 |
| *Melanthera nivea* | -85.96 | 15.08 |
| *Melanthera nivea* | -85.95 | 11.47 |
| *Melanthera nivea* | -85.93 | 11.38 |
| *Melanthera nivea* | -85.93 | 11.83 |
| *Melanthera nivea* | -85.92 | 10.86 |
| *Melanthera nivea* | -85.92 | 13.00 |
| *Melanthera nivea* | -85.92 | 13.00 |
| *Melanthera nivea* | -85.90 | 13.07 |
| *Melanthera nivea* | -85.90 | 11.41 |
| *Melanthera nivea* | -85.87 | 12.95 |
| *Melanthera nivea* | -85.86 | 10.86 |
| *Melanthera nivea* | -85.85 | 10.86 |
| *Melanthera nivea* | -85.85 | 12.95 |
| *Melanthera nivea* | -85.85 | 11.77 |
| *Melanthera nivea* | -85.83 | 12.98 |
| *Melanthera nivea* | -85.82 | 11.74 |
| *Melanthera nivea* | -85.82 | 13.02 |
| *Melanthera nivea* | -85.82 | 12.88 |
| *Melanthera nivea* | -85.80 | 10.50 |
| *Melanthera nivea* | -85.80 | 11.75 |
| *Melanthera nivea* | -85.80 | 13.00 |
| *Melanthera nivea* | -85.80 | 10.51 |
| *Melanthera nivea* | -85.78 | 15.42 |
| *Melanthera nivea* | -85.78 | 11.13 |
| *Melanthera nivea* | -85.78 | 13.08 |
| *Melanthera nivea* | -85.78 | 11.15 |
| *Melanthera nivea* | -85.78 | 10.89 |
| *Melanthera nivea* | -85.78 | 10.89 |
| *Melanthera nivea* | -85.77 | 10.85 |
| *Melanthera nivea* | -85.77 | 13.72 |
| *Melanthera nivea* | -85.75 | 13.50 |
| *Melanthera nivea* | -85.75 | 13.07 |
| *Melanthera nivea* | -85.74 | 10.87 |
| *Melanthera nivea* | -85.73 | 13.52 |
| *Melanthera nivea* | -85.72 | 12.67 |
| *Melanthera nivea* | -85.72 | 10.87 |
| *Melanthera nivea* | -85.72 | 13.22 |
| *Melanthera nivea* | -85.71 | 11.05 |
| *Melanthera nivea* | -85.71 | 10.00 |
| *Melanthera nivea* | -85.70 | 10.89 |
| *Melanthera nivea* | -85.70 | 9.99 |
| *Melanthera nivea* | -85.70 | 10.90 |
| *Melanthera nivea* | -85.70 | 10.88 |
| *Melanthera nivea* | -85.70 | 10.89 |
| *Melanthera nivea* | -85.68 | 13.37 |
| *Melanthera nivea* | -85.68 | 10.83 |
| *Melanthera nivea* | -85.67 | 11.18 |
| *Melanthera nivea* | -85.67 | 12.47 |
| *Melanthera nivea* | -85.66 | 10.80 |
| *Melanthera nivea* | -85.66 | 10.89 |
| *Melanthera nivea* | -85.64 | 10.80 |
| *Melanthera nivea* | -85.63 | 13.23 |
| *Melanthera nivea* | -85.63 | 13.60 |
| *Melanthera nivea* | -85.62 | 10.16 |
| *Melanthera nivea* | -85.62 | 10.16 |
| *Melanthera nivea* | -85.62 | 10.16 |
| *Melanthera nivea* | -85.62 | 10.83 |
| *Melanthera nivea* | -85.62 | 13.52 |
| *Melanthera nivea* | -85.61 | 10.87 |
| *Melanthera nivea* | -85.59 | 11.04 |
| *Melanthera nivea* | -85.58 | 10.83 |
| *Melanthera nivea* | -85.58 | 10.87 |
| *Melanthera nivea* | -85.58 | 10.16 |
| *Melanthera nivea* | -85.58 | 10.83 |
| *Melanthera nivea* | -85.58 | 10.87 |
| *Melanthera nivea* | -85.55 | 12.40 |
| *Melanthera nivea* | -85.55 | 12.63 |
| *Melanthera nivea* | -85.52 | 12.38 |
| *Melanthera nivea* | -85.50 | 12.94 |
| *Melanthera nivea* | -85.47 | 10.90 |
| *Melanthera nivea* | -85.47 | 10.93 |
| *Melanthera nivea* | -85.43 | 14.10 |
| *Melanthera nivea* | -85.43 | 10.62 |
| *Melanthera nivea* | -85.42 | 12.20 |
| *Melanthera nivea* | -85.38 | 12.28 |
| *Melanthera nivea* | -85.38 | 12.28 |
| *Melanthera nivea* | -85.37 | 9.84 |
| *Melanthera nivea* | -85.37 | 10.34 |
| *Melanthera nivea* | -85.37 | 10.51 |
| *Melanthera nivea* | -85.37 | 13.85 |
| *Melanthera nivea* | -85.37 | 10.78 |
| *Melanthera nivea* | -85.36 | 9.86 |
| *Melanthera nivea* | -85.36 | 9.86 |
| *Melanthera nivea* | -85.36 | 9.84 |
| *Melanthera nivea* | -85.33 | 12.08 |
| *Melanthera nivea* | -85.32 | 14.23 |
| *Melanthera nivea* | -85.32 | 10.25 |
| *Melanthera nivea* | -85.32 | 10.53 |
| *Melanthera nivea* | -85.31 | 10.55 |
| *Melanthera nivea* | -85.28 | 12.20 |
| *Melanthera nivea* | -85.28 | 10.76 |
| *Melanthera nivea* | -85.28 | 10.79 |
| *Melanthera nivea* | -85.27 | 9.95 |
| *Melanthera nivea* | -85.27 | 10.79 |
| *Melanthera nivea* | -85.27 | 12.92 |
| *Melanthera nivea* | -85.25 | 10.91 |
| *Melanthera nivea* | -85.20 | 10.22 |
| *Melanthera nivea* | -85.20 | 10.22 |
| *Melanthera nivea* | -85.20 | 10.22 |
| *Melanthera nivea* | -85.20 | 10.22 |
| *Melanthera nivea* | -85.18 | 13.57 |
| *Melanthera nivea* | -85.17 | 10.34 |
| *Melanthera nivea* | -85.17 | 14.22 |
| *Melanthera nivea* | -85.15 | 14.47 |
| *Melanthera nivea* | -85.14 | 9.60 |
| *Melanthera nivea* | -85.13 | 10.45 |
| *Melanthera nivea* | -85.10 | 9.58 |
| *Melanthera nivea* | -85.10 | 9.58 |
| *Melanthera nivea* | -85.10 | 9.58 |
| *Melanthera nivea* | -85.08 | 12.07 |
| *Melanthera nivea* | -85.06 | 13.74 |
| *Melanthera nivea* | -84.97 | 13.73 |
| *Melanthera nivea* | -84.97 | 13.73 |
| *Melanthera nivea* | -84.96 | 13.74 |
| *Melanthera nivea* | -84.93 | 13.75 |
| *Melanthera nivea* | -84.90 | 9.95 |
| *Melanthera nivea* | -84.85 | 10.37 |
| *Melanthera nivea* | -84.85 | 13.70 |
| *Melanthera nivea* | -84.85 | 10.36 |
| *Melanthera nivea* | -84.84 | 10.25 |
| *Melanthera nivea* | -84.84 | 10.25 |
| *Melanthera nivea* | -84.84 | 10.21 |
| *Melanthera nivea* | -84.84 | 13.73 |
| *Melanthera nivea* | -84.84 | 10.25 |
| *Melanthera nivea* | -84.83 | 10.25 |
| *Melanthera nivea* | -84.83 | 10.25 |
| *Melanthera nivea* | -84.82 | 13.67 |
| *Melanthera nivea* | -84.82 | 13.72 |
| *Melanthera nivea* | -84.81 | 13.72 |
| *Melanthera nivea* | -84.80 | 10.30 |
| *Melanthera nivea* | -84.80 | 10.30 |
| *Melanthera nivea* | -84.80 | 10.30 |
| *Melanthera nivea* | -84.80 | 13.63 |
| *Melanthera nivea* | -84.80 | 13.65 |
| *Melanthera nivea* | -84.80 | 10.28 |
| *Melanthera nivea* | -84.79 | 10.27 |
| *Melanthera nivea* | -84.79 | 10.32 |
| *Melanthera nivea* | -84.73 | 10.02 |
| *Melanthera nivea* | -84.71 | 9.89 |
| *Melanthera nivea* | -84.70 | 9.88 |
| *Melanthera nivea* | -84.68 | 9.58 |
| *Melanthera nivea* | -84.62 | 9.59 |
| *Melanthera nivea* | -84.61 | 9.77 |
| *Melanthera nivea* | -84.61 | 9.78 |
| *Melanthera nivea* | -84.61 | 9.78 |
| *Melanthera nivea* | -84.60 | 10.38 |
| *Melanthera nivea* | -84.58 | 9.75 |
| *Melanthera nivea* | -84.57 | 9.81 |
| *Melanthera nivea* | -84.57 | 14.03 |
| *Melanthera nivea* | -84.52 | 10.08 |
| *Melanthera nivea* | -84.52 | 14.00 |
| *Melanthera nivea* | -84.52 | 14.00 |
| *Melanthera nivea* | -84.48 | 12.12 |
| *Melanthera nivea* | -84.47 | 11.04 |
| *Melanthera nivea* | -84.47 | 10.09 |
| *Melanthera nivea* | -84.47 | 10.09 |
| *Melanthera nivea* | -84.47 | 10.06 |
| *Melanthera nivea* | -84.46 | 9.82 |
| *Melanthera nivea* | -84.45 | 11.57 |
| *Melanthera nivea* | -84.43 | 11.67 |
| *Melanthera nivea* | -84.40 | 10.37 |
| *Melanthera nivea* | -84.37 | 11.07 |
| *Melanthera nivea* | -84.36 | 11.02 |
| *Melanthera nivea* | -84.35 | 11.12 |
| *Melanthera nivea* | -84.35 | 11.07 |
| *Melanthera nivea* | -84.33 | 10.97 |
| *Melanthera nivea* | -84.30 | 11.10 |
| *Melanthera nivea* | -84.29 | 9.86 |
| *Melanthera nivea* | -84.29 | 9.86 |
| *Melanthera nivea* | -84.29 | 9.86 |
| *Melanthera nivea* | -84.27 | 9.90 |
| *Melanthera nivea* | -84.27 | 9.92 |
| *Melanthera nivea* | -84.26 | 13.97 |
| *Melanthera nivea* | -84.23 | 12.18 |
| *Melanthera nivea* | -84.23 | 12.17 |
| *Melanthera nivea* | -84.22 | 9.93 |
| *Melanthera nivea* | -84.22 | 11.42 |
| *Melanthera nivea* | -84.21 | 9.93 |
| *Melanthera nivea* | -84.20 | 11.13 |
| *Melanthera nivea* | -84.19 | 9.94 |
| *Melanthera nivea* | -84.19 | 9.94 |
| *Melanthera nivea* | -84.17 | 10.28 |
| *Melanthera nivea* | -84.17 | 10.27 |
| *Melanthera nivea* | -84.16 | 9.90 |
| *Melanthera nivea* | -84.16 | 9.59 |
| *Melanthera nivea* | -84.16 | 9.93 |
| *Melanthera nivea* | -84.15 | 9.96 |
| *Melanthera nivea* | -84.13 | 9.97 |
| *Melanthera nivea* | -84.08 | 9.97 |
| *Melanthera nivea* | -84.08 | 11.53 |
| *Melanthera nivea* | -84.08 | 9.93 |
| *Melanthera nivea* | -84.07 | 10.45 |
| *Melanthera nivea* | -84.07 | 9.59 |
| *Melanthera nivea* | -84.06 | 9.59 |
| *Melanthera nivea* | -84.03 | 10.43 |
| *Melanthera nivea* | -84.03 | 10.43 |
| *Melanthera nivea* | -84.02 | 11.58 |
| *Melanthera nivea* | -84.02 | 11.37 |
| *Melanthera nivea* | -84.01 | 10.45 |
| *Melanthera nivea* | -84.00 | 10.43 |
| *Melanthera nivea* | -84.00 | 10.43 |
| *Melanthera nivea* | -84.00 | 10.44 |
| *Melanthera nivea* | -83.94 | 9.89 |
| *Melanthera nivea* | -83.94 | 9.47 |
| *Melanthera nivea* | -83.92 | 13.13 |
| *Melanthera nivea* | -83.85 | 11.60 |
| *Melanthera nivea* | -83.85 | 11.58 |
| *Melanthera nivea* | -83.85 | 10.18 |
| *Melanthera nivea* | -83.84 | 10.18 |
| *Melanthera nivea* | -83.80 | 11.53 |
| *Melanthera nivea* | -83.78 | 11.02 |
| *Melanthera nivea* | -83.77 | 11.00 |
| *Melanthera nivea* | -83.73 | 10.95 |
| *Melanthera nivea* | -83.67 | 11.67 |
| *Melanthera nivea* | -83.64 | 9.39 |
| *Melanthera nivea* | -83.59 | 8.48 |
| *Melanthera nivea* | -83.58 | 8.47 |
| *Melanthera nivea* | -83.58 | 8.46 |
| *Melanthera nivea* | -83.52 | 8.61 |
| *Melanthera nivea* | -83.52 | 8.61 |
| *Melanthera nivea* | -83.51 | 8.69 |
| *Melanthera nivea* | -83.50 | 9.02 |
| *Melanthera nivea* | -83.50 | 9.02 |
| *Melanthera nivea* | -83.50 | 10.51 |
| *Melanthera nivea* | -83.50 | 8.69 |
| *Melanthera nivea* | -83.50 | 9.83 |
| *Melanthera nivea* | -83.50 | 8.56 |
| *Melanthera nivea* | -83.49 | 8.45 |
| *Melanthera nivea* | -83.49 | 8.46 |
| *Melanthera nivea* | -83.48 | 8.69 |
| *Melanthera nivea* | -83.47 | 8.48 |
| *Melanthera nivea* | -83.47 | 8.48 |
| *Melanthera nivea* | -83.46 | 9.96 |
| *Melanthera nivea* | -83.46 | 9.96 |
| *Melanthera nivea* | -83.46 | 8.48 |
| *Melanthera nivea* | -83.46 | 9.96 |
| *Melanthera nivea* | -83.34 | 8.55 |
| *Melanthera nivea* | -83.34 | 8.55 |
| *Melanthera nivea* | -83.28 | 9.63 |
| *Melanthera nivea* | -83.28 | 9.62 |
| *Melanthera nivea* | -83.26 | 9.62 |
| *Melanthera nivea* | -83.26 | 9.62 |
| *Melanthera nivea* | -83.25 | 9.05 |
| *Melanthera nivea* | -83.25 | 9.05 |
| *Melanthera nivea* | -83.25 | 9.05 |
| *Melanthera nivea* | -83.24 | 9.05 |
| *Melanthera nivea* | -83.21 | 14.31 |
| *Melanthera nivea* | -83.07 | 12.17 |
| *Melanthera nivea* | -83.06 | 12.18 |
| *Melanthera nivea* | -83.05 | 12.17 |
| *Melanthera nivea* | -83.05 | 12.18 |
| *Melanthera nivea* | -83.05 | 9.44 |
| *Melanthera nivea* | -83.03 | 9.67 |
| *Melanthera nivea* | -83.03 | 9.68 |
| *Melanthera nivea* | -83.02 | 9.67 |
| *Melanthera nivea* | -82.97 | 8.78 |
| *Melanthera nivea* | -82.97 | 8.79 |
| *Melanthera nivea* | -82.97 | 8.79 |
| *Melanthera nivea* | -82.96 | 9.03 |
| *Melanthera nivea* | -82.96 | 9.03 |
| *Melanthera nivea* | -82.96 | 8.78 |
| *Melanthera nivea* | -82.96 | 8.78 |
| *Melanthera nivea* | -82.96 | 9.51 |
| *Melanthera nivea* | -82.96 | 9.51 |
| *Melanthera nivea* | -82.96 | 8.79 |
| *Melanthera nivea* | -82.95 | 9.87 |
| *Melanthera nivea* | -82.95 | 9.86 |
| *Melanthera nivea* | -82.94 | 9.75 |
| *Melanthera nivea* | -82.93 | 8.73 |
| *Melanthera nivea* | -82.90 | 9.80 |
| *Melanthera nivea* | -82.85 | 8.96 |
| *Melanthera nivea* | -82.84 | 9.73 |
| *Melanthera nivea* | -82.83 | 9.74 |
| *Melanthera nivea* | -82.73 | 9.57 |
| *Melanthera nivea* | -82.71 | 9.63 |
| *Melanthera nivea* | -82.70 | 9.63 |
| *Melanthera nivea* | -82.67 | 9.63 |
| *Melanthera nivea* | -82.62 | 9.61 |
| *Melanthera nivea* | -82.48 | 8.83 |
| *Melanthera nivea* | -82.47 | 8.82 |
| *Melanthera nivea* | -82.45 | 9.45 |
| *Melanthera nivea* | -82.45 | 9.31 |
| *Melanthera nivea* | -82.43 | 8.77 |
| *Melanthera nivea* | -82.42 | 9.29 |
| *Melanthera nivea* | -82.42 | 8.73 |
| *Melanthera nivea* | -82.42 | 8.78 |
| *Melanthera nivea* | -82.41 | 23.13 |
| *Melanthera nivea* | -82.40 | 8.80 |
| *Melanthera nivea* | -82.40 | 9.30 |
| *Melanthera nivea* | -82.39 | 22.97 |
| *Melanthera nivea* | -82.38 | 9.25 |
| *Melanthera nivea* | -82.38 | 9.25 |
| *Melanthera nivea* | -82.38 | 22.98 |
| *Melanthera nivea* | -82.37 | 23.11 |
| *Melanthera nivea* | -82.27 | 22.82 |
| *Melanthera nivea* | -82.17 | 8.92 |
| *Melanthera nivea* | -82.15 | 9.32 |
| *Melanthera nivea* | -81.34 | 8.78 |
| *Melanthera nivea* | -81.25 | 8.17 |
| *Melanthera nivea* | -81.13 | 8.52 |
| *Melanthera nivea* | -81.12 | 8.52 |
| *Melanthera nivea* | -81.12 | 8.51 |
| *Melanthera nivea* | -81.10 | 8.48 |
| *Melanthera nivea* | -81.08 | 8.49 |
| *Melanthera nivea* | -80.68 | 8.17 |
| *Melanthera nivea* | -80.53 | 8.94 |
| *Melanthera nivea* | -80.52 | 7.25 |
| *Melanthera nivea* | -80.18 | 9.18 |
| *Melanthera nivea* | -80.13 | 9.21 |
| *Melanthera nivea* | -80.12 | 8.63 |
| *Melanthera nivea* | -80.12 | 8.60 |
| *Melanthera nivea* | -80.12 | 8.63 |
| *Melanthera nivea* | -80.11 | 8.64 |
| *Melanthera nivea* | -80.10 | 9.15 |
| *Melanthera nivea* | -80.08 | 7.60 |
| *Melanthera nivea* | -80.07 | 8.58 |
| *Melanthera nivea* | -80.05 | 9.23 |
| *Melanthera nivea* | -80.02 | 9.22 |
| *Melanthera nivea* | -80.00 | 9.30 |
| *Melanthera nivea* | -79.85 | 9.15 |
| *Melanthera nivea* | -79.84 | 9.14 |
| *Melanthera nivea* | -79.83 | 9.16 |
| *Melanthera nivea* | -79.75 | 9.17 |
| *Melanthera nivea* | -79.73 | 9.14 |
| *Melanthera nivea* | -79.70 | 9.12 |
| *Melanthera nivea* | -79.70 | -1.27 |
| *Melanthera nivea* | -79.68 | 9.14 |
| *Melanthera nivea* | -79.67 | 9.55 |
| *Melanthera nivea* | -79.67 | 9.55 |
| *Melanthera nivea* | -79.66 | 9.08 |
| *Melanthera nivea* | -79.66 | 9.07 |
| *Melanthera nivea* | -79.65 | 9.55 |
| *Melanthera nivea* | -79.63 | 9.53 |
| *Melanthera nivea* | -79.61 | 9.02 |
| *Melanthera nivea* | -79.60 | 9.20 |
| *Melanthera nivea* | -79.60 | -2.40 |
| *Melanthera nivea* | -79.58 | 9.60 |
| *Melanthera nivea* | -79.58 | 8.95 |
| *Melanthera nivea* | -79.57 | 9.04 |
| *Melanthera nivea* | -79.56 | 8.98 |
| *Melanthera nivea* | -79.56 | 9.61 |
| *Melanthera nivea* | -79.56 | 9.19 |
| *Melanthera nivea* | -79.55 | 9.10 |
| *Melanthera nivea* | -79.55 | 9.09 |
| *Melanthera nivea* | -79.55 | 8.96 |
| *Melanthera nivea* | -79.53 | 9.09 |
| *Melanthera nivea* | -79.52 | 8.98 |
| *Melanthera nivea* | -79.48 | -2.22 |
| *Melanthera nivea* | -79.44 | 9.08 |
| *Melanthera nivea* | -79.41 | 9.17 |
| *Melanthera nivea* | -79.38 | 9.08 |
| *Melanthera nivea* | -79.37 | -0.58 |
| *Melanthera nivea* | -79.36 | 9.11 |
| *Melanthera nivea* | -79.25 | -0.08 |
| *Melanthera nivea* | -79.04 | 21.62 |
| *Melanthera nivea* | -79.00 | 1.05 |
| *Melanthera nivea* | -78.90 | 0.75 |
| *Melanthera nivea* | -78.77 | -1.43 |
| *Melanthera nivea* | -78.54 | 8.98 |
| *Melanthera nivea* | -78.53 | 1.17 |
| *Melanthera nivea* | -78.53 | 1.17 |
| *Melanthera nivea* | -78.43 | 1.68 |
| *Melanthera nivea* | -78.27 | 0.97 |
| *Melanthera nivea* | -78.27 | 0.97 |
| *Melanthera nivea* | -78.24 | 9.32 |
| *Melanthera nivea* | -78.22 | 2.18 |
| *Melanthera nivea* | -78.03 | 9.23 |
| *Melanthera nivea* | -77.98 | 1.60 |
| *Melanthera nivea* | -77.92 | -6.99 |
| *Melanthera nivea* | -77.68 | 7.75 |
| *Melanthera nivea* | -77.68 | 8.18 |
| *Melanthera nivea* | -77.57 | 17.94 |
| *Melanthera nivea* | -77.45 | 25.07 |
| *Melanthera nivea* | -77.45 | 8.35 |
| *Melanthera nivea* | -77.41 | 6.12 |
| *Melanthera nivea* | -77.37 | 6.12 |
| *Melanthera nivea* | -77.37 | 8.66 |
| *Melanthera nivea* | -77.33 | 6.13 |
| *Melanthera nivea* | -77.28 | 4.42 |
| *Melanthera nivea* | -77.18 | 7.28 |
| *Melanthera nivea* | -77.17 | 4.17 |
| *Melanthera nivea* | -77.11 | 8.36 |
| *Melanthera nivea* | -77.00 | 3.92 |
| *Melanthera nivea* | -76.73 | 3.59 |
| *Melanthera nivea* | -76.73 | 8.10 |
| *Melanthera nivea* | -76.72 | 8.10 |
| *Melanthera nivea* | -76.72 | 8.10 |
| *Melanthera nivea* | -76.62 | 4.93 |
| *Melanthera nivea* | -76.60 | 3.60 |
| *Melanthera nivea* | -76.58 | 5.78 |
| *Melanthera nivea* | -76.55 | 3.27 |
| *Melanthera nivea* | -76.45 | 7.29 |
| *Melanthera nivea* | -76.45 | 7.29 |
| *Melanthera nivea* | -76.43 | 6.35 |
| *Melanthera nivea* | -76.38 | 4.89 |
| *Melanthera nivea* | -76.29 | 4.87 |
| *Melanthera nivea* | -76.29 | 4.87 |
| *Melanthera nivea* | -76.16 | 5.86 |
| *Melanthera nivea* | -76.08 | 5.34 |
| *Melanthera nivea* | -75.97 | 5.99 |
| *Melanthera nivea* | -75.97 | 5.98 |
| *Melanthera nivea* | -75.87 | 0.08 |
| *Melanthera nivea* | -75.87 | 0.08 |
| *Melanthera nivea* | -75.87 | -0.08 |
| *Melanthera nivea* | -75.87 | -0.08 |
| *Melanthera nivea* | -75.87 | 0.08 |
| *Melanthera nivea* | -75.74 | 5.96 |
| *Melanthera nivea* | -75.74 | 5.96 |
| *Melanthera nivea* | -75.67 | 5.88 |
| *Melanthera nivea* | -75.67 | 5.88 |
| *Melanthera nivea* | -75.67 | 2.26 |
| *Melanthera nivea* | -75.67 | 2.26 |
| *Melanthera nivea* | -75.66 | 5.88 |
| *Melanthera nivea* | -75.54 | 1.69 |
| *Melanthera nivea* | -75.54 | 1.69 |
| *Melanthera nivea* | -75.43 | 10.35 |
| *Melanthera nivea* | -75.43 | 10.34 |
| *Melanthera nivea* | -75.22 | 10.65 |
| *Melanthera nivea* | -75.15 | 6.02 |
| *Melanthera nivea* | -75.14 | 6.01 |
| *Melanthera nivea* | -75.07 | 9.97 |
| *Melanthera nivea* | -75.04 | 5.15 |
| *Melanthera nivea* | -75.04 | 5.15 |
| *Melanthera nivea* | -74.95 | 11.00 |
| *Melanthera nivea* | -74.94 | 10.65 |
| *Melanthera nivea* | -74.83 | 4.40 |
| *Melanthera nivea* | -74.83 | 4.41 |
| *Melanthera nivea* | -74.83 | 4.41 |
| *Melanthera nivea* | -74.83 | 4.38 |
| *Melanthera nivea* | -74.80 | 4.42 |
| *Melanthera nivea* | -74.80 | 4.42 |
| *Melanthera nivea* | -74.70 | 7.09 |
| *Melanthera nivea* | -74.64 | 6.54 |
| *Melanthera nivea* | -74.56 | 4.35 |
| *Melanthera nivea* | -74.56 | 4.35 |
| *Melanthera nivea* | -74.50 | 4.18 |
| *Melanthera nivea* | -74.50 | 4.18 |
| *Melanthera nivea* | -74.50 | -4.53 |
| *Melanthera nivea* | -74.50 | 20.35 |
| *Melanthera nivea* | -74.48 | 4.19 |
| *Melanthera nivea* | -74.48 | 4.19 |
| *Melanthera nivea* | -74.43 | 4.97 |
| *Melanthera nivea* | -74.43 | 4.71 |
| *Melanthera nivea* | -74.33 | 4.60 |
| *Melanthera nivea* | -74.17 | 10.65 |
| *Melanthera nivea* | -74.15 | 10.88 |
| *Melanthera nivea* | -73.62 | 8.31 |
| *Melanthera nivea* | -73.33 | 7.08 |
| *Melanthera nivea* | -73.15 | 7.21 |
| *Melanthera nivea* | -72.91 | 4.10 |
| *Melanthera nivea* | -72.31 | 8.20 |
| *Melanthera nivea* | -71.83 | -3.33 |
| *Melanthera nivea* | -71.83 | -3.33 |
| *Melanthera nivea* | -71.78 | 18.68 |
| *Melanthera nivea* | -71.75 | 18.15 |
| *Melanthera nivea* | -71.67 | -3.17 |
| *Melanthera nivea* | -71.33 | 12.17 |
| *Melanthera nivea* | -71.20 | 18.02 |
| *Melanthera nivea* | -71.20 | 18.02 |
| *Melanthera nivea* | -70.85 | 19.00 |
| *Melanthera nivea* | -70.75 | 18.90 |
| *Melanthera nivea* | -70.73 | 19.07 |
| *Melanthera nivea* | -70.62 | 19.09 |
| *Melanthera nivea* | -70.62 | 19.08 |
| *Melanthera nivea* | -70.60 | 19.08 |
| *Melanthera nivea* | -70.60 | 19.08 |
| *Melanthera nivea* | -70.52 | 18.65 |
| *Melanthera nivea* | -70.48 | 18.89 |
| *Melanthera nivea* | -70.47 | 18.35 |
| *Melanthera nivea* | -70.47 | 18.52 |
| *Melanthera nivea* | -70.37 | 18.42 |
| *Melanthera nivea* | -70.30 | 18.33 |
| *Melanthera nivea* | -70.25 | -3.85 |
| *Melanthera nivea* | -69.98 | -4.10 |
| *Melanthera nivea* | -69.88 | 18.65 |
| *Melanthera nivea* | -69.88 | 18.65 |
| *Melanthera nivea* | -69.78 | 18.48 |
| *Melanthera nivea* | -69.78 | 18.48 |
| *Melanthera nivea* | -69.55 | 9.47 |
| *Melanthera nivea* | -69.50 | -4.70 |
| *Melanthera nivea* | -69.50 | -4.70 |
| *Melanthera nivea* | -69.34 | -3.62 |
| *Melanthera nivea* | -69.34 | -3.62 |
| *Melanthera nivea* | -69.10 | 18.44 |
| *Melanthera nivea* | -68.88 | 18.43 |
| *Melanthera nivea* | -68.88 | 18.43 |
| *Melanthera nivea* | -68.87 | -3.38 |
| *Melanthera nivea* | -68.27 | 10.87 |
| *Melanthera nivea* | -68.25 | 10.92 |
| *Melanthera nivea* | -67.97 | 10.50 |
| *Melanthera nivea* | -67.42 | 8.93 |
| *Melanthera nivea* | -67.17 | 18.28 |
| *Melanthera nivea* | -67.11 | 18.01 |
| *Melanthera nivea* | -66.98 | 18.17 |
| *Melanthera nivea* | -66.95 | 18.09 |
| *Melanthera nivea* | -66.87 | 18.26 |
| *Melanthera nivea* | -66.84 | 18.01 |
| *Melanthera nivea* | -66.77 | 18.14 |
| *Melanthera nivea* | -66.72 | 18.17 |
| *Melanthera nivea* | -66.72 | 18.06 |
| *Melanthera nivea* | -66.52 | 17.89 |
| *Melanthera nivea* | -66.47 | 10.07 |
| *Melanthera nivea* | -66.44 | 18.47 |
| *Melanthera nivea* | -66.42 | 18.46 |
| *Melanthera nivea* | -66.27 | 18.45 |
| *Melanthera nivea* | -66.23 | 18.09 |
| *Melanthera nivea* | -66.23 | 18.09 |
| *Melanthera nivea* | -66.17 | 18.31 |
| *Melanthera nivea* | -66.17 | 18.36 |
| *Melanthera nivea* | -66.16 | 18.38 |
| *Melanthera nivea* | -66.11 | 17.98 |
| *Melanthera nivea* | -66.10 | 18.15 |
| *Melanthera nivea* | -66.10 | 18.15 |
| *Melanthera nivea* | -66.08 | 18.43 |
| *Melanthera nivea* | -66.06 | 18.39 |
| *Melanthera nivea* | -66.01 | 18.04 |
| *Melanthera nivea* | -65.95 | 10.09 |
| *Melanthera nivea* | -65.84 | 18.19 |
| *Melanthera nivea* | -65.77 | 18.38 |
| *Melanthera nivea* | -65.75 | 18.34 |
| *Melanthera nivea* | -65.72 | 18.19 |
| *Melanthera nivea* | -65.66 | 18.32 |
| *Melanthera nivea* | -65.63 | 18.34 |
| *Melanthera nivea* | -65.62 | 18.34 |
| *Melanthera nivea* | -65.57 | 18.38 |
| *Melanthera nivea* | -65.48 | 18.10 |
| *Melanthera nivea* | -65.46 | 18.10 |
| *Melanthera nivea* | -65.44 | 18.15 |
| *Melanthera nivea* | -64.71 | -3.35 |
| *Melanthera nivea* | -62.89 | 9.90 |
| *Melanthera nivea* | -62.74 | 17.34 |
| *Melanthera nivea* | -62.50 | 17.17 |
| *Melanthera nivea* | -61.30 | 15.55 |
| *Melanthera nivea* | -61.02 | 14.65 |
| *Melanthera nivea* | -61.02 | -13.54 |
| *Melanthera nivea* | -60.77 | 9.14 |
| *Melanthera nivea* | -59.51 | 13.21 |
| *Melanthera nivea* | -55.00 | -10.00 |
| *Melanthera nivea* | -52.87 | -10.83 |
| *Melanthera nivea* | -51.07 | -0.37 |
| *Melanthera nivea* | -42.80 | -5.09 |
| *Melanthera nivea* | -37.91 | -6.21 |
| *Melanthera nivea* | -37.86 | -5.72 |
| *Melanthera nivea* | -37.51 | -5.44 |
| *Melanthera nivea* | -37.51 | -6.08 |
| *Melanthera nivea* | -37.03 | -5.17 |
| *Melanthera nivea* | -37.03 | -5.17 |
