# Supplementary material for: Importance of biotic predictors in estimation of potential invasive areas: the example of the tortoise beetle Eurypedus nigrosignatus, in Hispaniola
Source: PeerJ. 2018 Dec 5;6:e6052. doi: 10.7717/peerj.6052 (PMC6286658; doi:10.7717/peerj.6052)
Supplement: Supplemental Information 3 [file peerj-06-6052-s003.docx]

**Importance of biotic predictors in estimation of potential invasive areas: the example of the tortoise beetle *Eurypedus nigrosignatus,* in Hispaniola**

Marianna V. P. Simões & A. Townsend Peterson

**Supporting information**

**Supporting information, Table S3.** Mean AUC ratios (AUC), and partial ROC analyses for different candidate models of the study on different levels of regularization parameter (*β*).
